# QoRTs: a comprehensive toolset for quality control and data processing of RNA-Seq experiments

**DOI:** 10.1186/s12859-015-0670-5

**Published:** 2015-07-19

**Authors:** Stephen W. Hartley, James C. Mullikin

**Affiliations:** Comparative Genomics Analysis Unit, Cancer Genetics and Comparative Genomics Branch, National Human Genome Research Institute, National Institutes of Health, Bethesda, MD 20892 USA

**Keywords:** Quality Control, RNA-Seq, Next-generation sequencing, Differential expression, Differential transcript regulation, Differential splicing

## Abstract

**Background:**

High-throughput next-generation RNA sequencing has matured into a viable and powerful method for detecting variations in transcript expression and regulation. Proactive quality control is of critical importance as unanticipated biases, artifacts, or errors can potentially drive false associations and lead to flawed results.

**Results:**

We have developed the Quality of RNA-Seq Toolset, or QoRTs, a comprehensive, multifunction toolset that assists in quality control and data processing of high-throughput RNA sequencing data.

**Conclusions:**

QoRTs generates an unmatched variety of quality control metrics, and can provide cross-comparisons of replicates contrasted by batch, biological sample, or experimental condition, revealing any outliers and/or systematic issues that could drive false associations or otherwise compromise downstream analyses. In addition, QoRTs simultaneously replaces the functionality of numerous other data-processing tools, and can quickly and efficiently generate quality control metrics, coverage counts (for genes, exons, and known/novel splice-junctions), and browser tracks. These functions can all be carried out as part of a single unified data-processing/quality control run, greatly reducing both the complexity and the total runtime of the analysis pipeline. The software, source code, and documentation are available online at http://hartleys.github.io/QoRTs.

**Electronic supplementary material:**

The online version of this article (doi:10.1186/s12859-015-0670-5) contains supplementary material, which is available to authorized users.

## Background

High throughput next-generation sequencing of RNA (RNA-Seq) provides an unprecedented volume of transcriptomic information [1]. However, like all sequencing technologies, RNA-Seq is prone to certain biases, errors, and artifacts, necessitating robust and comprehensive quality control (QC).

In most cases, major biases will be predictable and can be accounted for in downstream analyses. Many inherent biases will uniformly affect all replicates, and thus may not invalidate cross-sample or cross-condition comparisons, depending on the analysis methodology used [2–4]. In other cases, it may be possible to correct or adjust for such biases [5, 6].

However, RNA-Seq is a complex multi-stage process with numerous potential modes of failure, both known and unknown. Mistakes or inconsistencies in sample prep, library creation, or in sequencing itself could potentially introduce unanticipated artifacts, biases, or errors that could lead to flawed results. In some cases such anomalies will be obvious, but in many cases major artifacts can be obfuscated by the sheer quantity of data involved. In these (presumably rare) instances, it is vital that such issues be detected so that they can be dealt with properly. However, as the full set of all possible problems that could ever arise with this technology is unknown, there is no comprehensive way to automatically test for data quality.

Two existing tools, RSeQC and RNA-SeQC, can be used to perform some quality control on RNA-Seq datasets [7, 8]. Other general-purpose tools can perform limited quality control on next-gen sequencing data, including RNA-Seq [9, 10]. While these tools can provide some of the functionality necessary to validate the quality of RNA-Seq data, they all have significant shortcomings that limit their utility.

Here we introduce QoRTs, the Quality of RNA-Seq ToolSet: a comprehensive, multifunction software package that generates a broad array of quality control metrics and allows bioinformaticians to view and compare RNA-Seq data across numerous replicates, organized and differentiated by batch, biological condition, library, read-group, and/or sample [11].

## Implementation

**Fig. 1:**
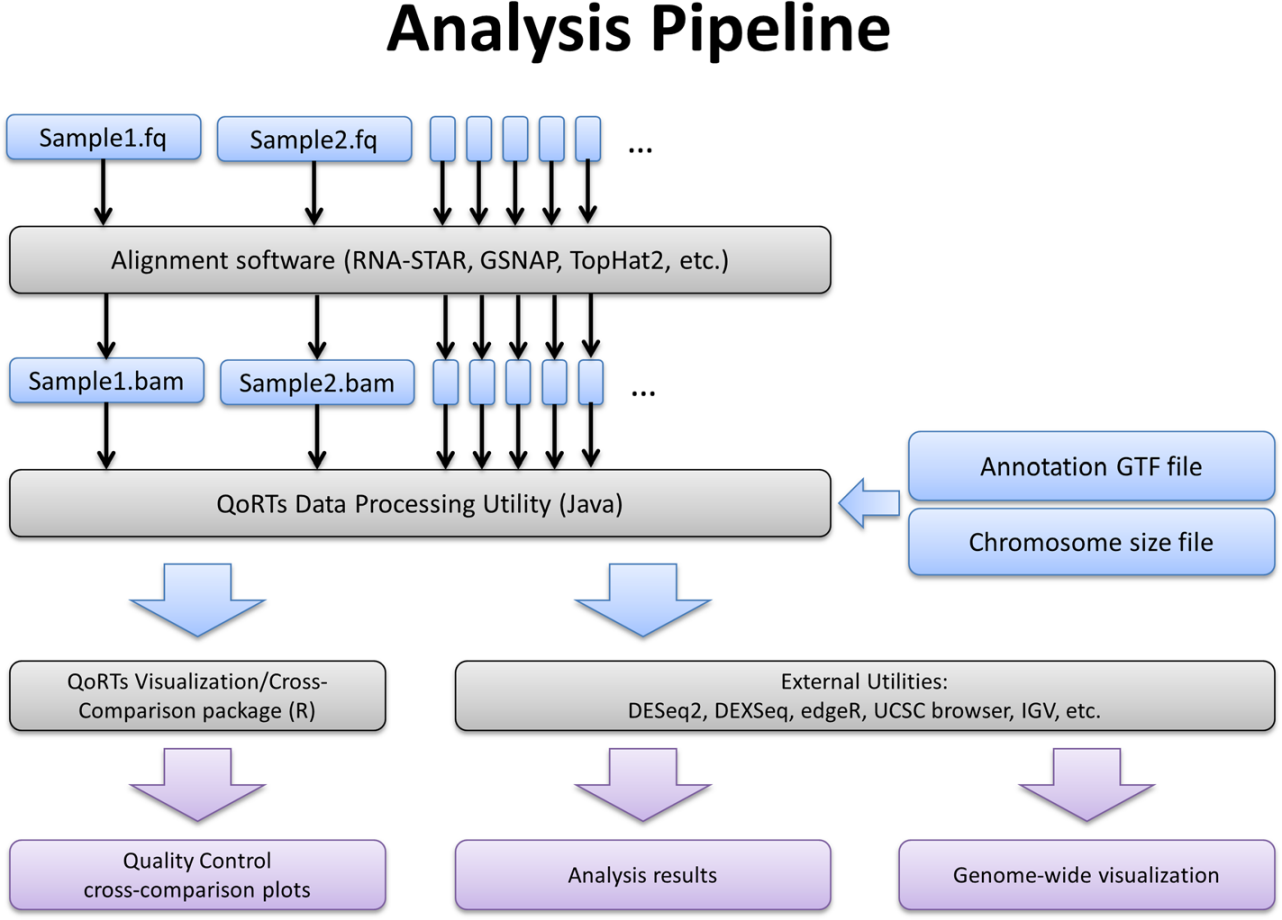
An example analysis pipeline with QoRTs. This flowchart illustrates the recommended analysis pipeline for conventional RNA-Seq analysis using QoRTs. Input and intermediary files are shown in blue, output files and results are shown in purple

The QoRTs software package consists of two distinct modules: a java package which performs most of the data processing and a companion R package for visualization and cross-replicate comparison. A recommended analysis pipeline is illustrated in Fig. 1.

All count files, QC statistics, and browser tracks for a given replicate can be generated using a single command and over a single pass through the alignment file, greatly streamlining the analysis pipeline. If desired, individual sub-functions can be deactivated to reduce runtime.

QoRTs is both fast and efficient: it can generate a comprehensive array of quality control metrics, browser tracks, summary plots, and read counts in 3–6 min per million read-pairs. For typical genomes and annotations the QoRTs data processing utility requires less than 4 gigabytes of free memory. The companion R-package (used for generating plots and pdf reports) has much lower resource requirements and can generally run on any desktop computer that can support R.

The java package was written in the Scala programming language and uses the Picard sam-jdk API [12]. However, since all necessary libraries are compiled to java bytecode and packaged in the distribution jar file, neither Scala nor Picard is required for use. QoRTs is designed to run on any machine that has both java (version 6 or higher, 64-bit) and R (3.0.2 or higher), without any additional dependencies.

### The importance of quality control

Quality control in bioinformatics is a contentious issue, and the necessity and utility of quality control metrics is often called into question. However, across the field of bioinformatics there are numerous cases where biases, artifacts, and other data quality issues have called results into question, sometimes resulting in retractions [13–19]. In many of these cases the problems were only identified when the study came under intense external scrutiny, and the specific issues at fault were not well-characterized up to that point. Such data-quality issues can sometimes be corrected, but only after they have been identified [20]. Thus: it is not sufficient to check for issues that are already well-known: quality control must be proactive and comprehensive.

RNA-Seq data in particular has numerous inherent sources of bias including hexamer bias, 3’ bias, GC bias, amplification bias, mapping bias, sequence-specific bias, and fragment-size bias [5, 6, 21]. While most advanced RNA-Seq analysis tools are designed with (at least some of) these effects in mind, they often still rely on the assumption these effects are consistent between samples and uniform between experimental conditions [2, 22–24]. Outliers, batch effects, and/or effects that vary disproportionately between the experimental conditions can still have the potential to drive false associations.

Without proactive and comprehensive quality control it is not possible to be certain that unobserved errors, biases, or artifacts do not violate the assumptions of downstream analyses.

### Quality control with QoRTs

Performing quality control with QoRTs requires two steps. First the (java-based) data-processing module is run on each replicate, and then the companion R package is used for visualization and cross-comparison of replicates.

**Fig. 2:**
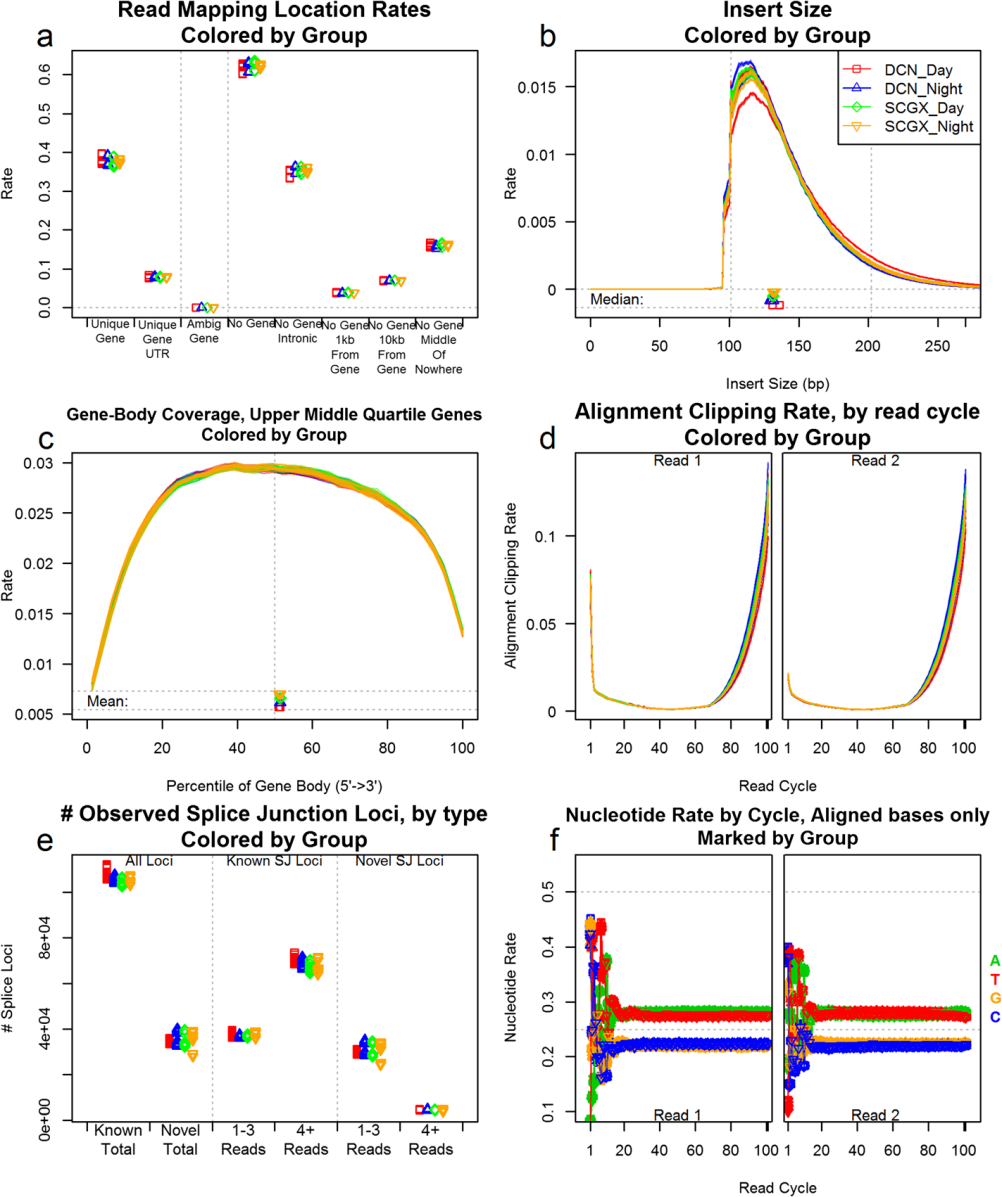
A small selection of the QC plots offered by QoRTs. This series includes 12 samples, each consisting of 6 technical replicates (for a total of 72 bam files), with 4 different biological conditions (3 samples per condition). In all nine plots, replicates are colored and differentiated by biological group. In the line plots (c,d,e, and f) the samples are simply colored by biological group. In other plots (a and g), replicates are differentiated by character, color, and horizontal offset. This differentiation allows easy identification of both outliers and systematic biases or errors associated with the biological condition. Such systematic errors are of particular importance as they could potentially drive false associations. A full description of each plot and its interpretation can be found in the supplementary materials

Simple multi-replicate plots that differentiate each replicate individually (as offered in a limited capacity by RSeQC and RNA-SeQC) may be adequate for small sample sizes; however, with larger or more complex studies these plots may be unreadable due to multi-plotting and insufficiently distinct coloration. QoRTs offers the ability to organize and differentiate replicate groups by sample, sequencer-lane/run, or any arbitrary grouping assigned by the user (such as biological condition). This allows easier identification of systematic biases and artifacts in large-scale datasets. By default QoRTs produces a battery of 34 plots, which are each described at length in the package user manual (Additional file 1) [25]. Fig. 2 includes a subset of these plots generated for a small example dataset of 72 replicates (12 samples, 6 technical replicates each). In this example, replicates are colored and differentiated by biological condition. The standard battery of QC plots can be automatically compiled into a single multi-frame image or as a printable pdf report.

The purpose of these various plots is to characterize the data in numerous ways, hopefully revealing any artifacts, outliers, batch effects, or phenodata-associated effects. In most cases any abnormalities should be revealed by multiple plots, and the various metrics can assist in identifying the underlying causes and assessing whether downstream analyses are likely to be adversely affected. The QoRTs user manual includes descriptions of various potential issues and how they could be recognized and differentiated using the available QC plots [25]. The user manual also includes an in-depth walkthrough of two examples in which QoRTs was used to identify actionable quality control issues in a real-world dataset.

**Fig. 3:**
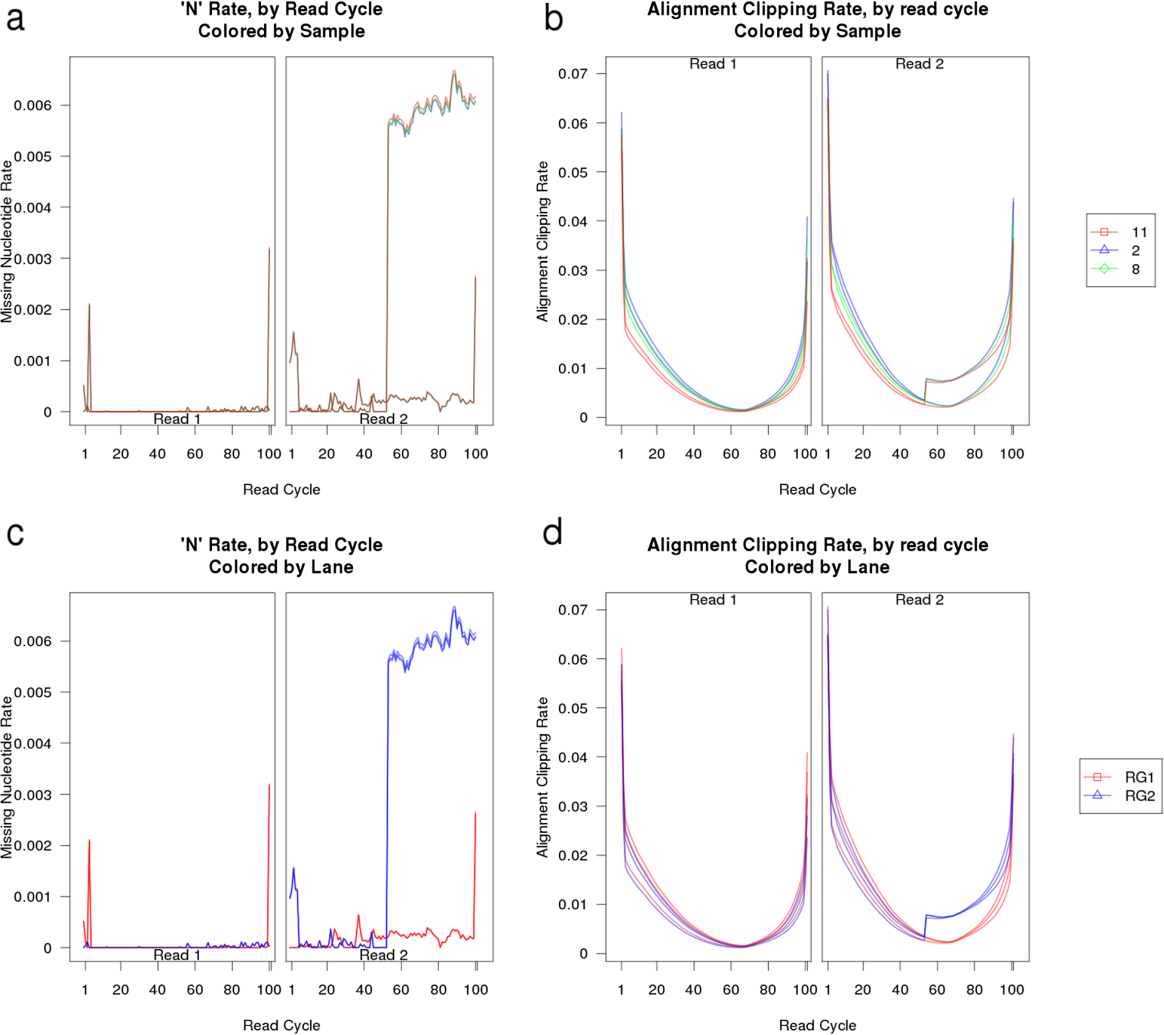
Example issue detected via QoRTs. A subset of the output plots from a dataset in which a rare hardware-level fault produced an actionable QC issue that can be easily identified via QoRTs. In (**a**) and (**b**) the replicates are colored by biological sample; in (**c**) and (**d**) replicates are colored by sequencer lane. See the QoRTs vignette for more information (Additional file 1)

In one such example, a shift in the sequencer scanner at cycle 53 of read 2 resulted in a small number of reads (less than 1 %) being truncated (Fig. 3). Using the array of information provided by QoRTs we can not only identify the presence of a QC issue, but also narrow down the root cause of the issue and predict its impact on downstream analyses. In this example, the issue manifested as a large increase in the rate of ‘N’ bases beginning at this cycle and continuing to the end of the read. Similarly, an abrupt increase in the alignment clipping rate was observed beginning at this cycle. The fact that the issue was specific to one lane (see Fig. 3c and d), rather than being specific to any particular sample (see Fig. 3a and b) implied that the issue likely originated at the sequencing step rather than at sample or library preparation. The fact that the alignment clipping rate jumped so dramatically at cycle 53 indicated that the root cause was a massive increase in the ‘N’ rate in a small subset of the reads, rather than being a more subtle increase distributed across all reads.

For most datasets these plots should not reveal anything of interest: RNA-Seq is a relatively mature technology and large-scale systematic errors should (theoretically) be rare. However, when such errors do occur it is critical that they be caught before the flawed data is analyzed and the results reported.

### Data processing for downstream analysis

In addition to its primary function as a quality control tool, QoRTs automatically generates all input read-count files needed for use with a number of differential expression/regulation analysis tools. Gene-level read counts are generated using the same methodology specified by HTSeq and reproduced in the Bioconductor GenomicRanges package (using the default “union” rule) [26, 27]. QoRTs also generates the exon-level counts and related annotation files required by DEXSeq [22].

QoRTs can also (optionally) produce a number of browser track files designed for use with the UCSC genome browser or the IGV viewer [28–30]. QoRTs produces “wiggle” files which can be used to view simple coverage depth across evenly-spaced windows across the genome (similar to those produced by the samtools “bam2wig” utility) and specialized “bed” files which display coverage depth bridging any known or novel splice junctions, providing functionality similar to the “sashimi” plots generated by IGV [30, 31]. QoRTs also provides tools for generating summary tracks that display mean normalized coverages across multiple samples.

### Comparison with existing tools

QoRTs offers and improves upon many of the features offered by the two other major RNA quality control tools: RSeQC and RNA-SeQC (see Table 1).

**Table 1:** Features and capabilities of QoRTs compared with those offered by other tools

	QoRTs	RSeQC	RNA-SeQC
Sequence Metrics:			
Quality score (by cycle)	Yes	Yes^1,*^	Yes
G/C content	Yes	Yes	Yes
Nucleotide vs cycle (NVC)	Yes	Yes^1^	No
N-rate by cycle	Yes	No	No
Unclipped NVC	Yes	No	No
Clipped Sequences NVC	Yes	No	No
Alignment Metrics:			
Strandedness	Yes	Yes^2^	Yes
Clipping Profile	Yes	Yes^1,*^	No
Insert Size	Yes	Yes^2,*^	Partial^3^
Cigar Op Profile	Yes	Partial^1,2,4,*^	No
Cigar Op Length Distribution	Yes	No	No
Gene / Exon Coverage			
Gene-Body Coverage	Yes	Yes^5,*^	Yes
Gene-Body Coverage, Low-/Medium-/High-expression genes	Yes	No	Yes
Mapping Location rates (intron, exon, UTR, etc.)	Yes	Yes	Partial
Gene Diversity	Yes	No	No
RPKM/FPKM	Yes	Yes^*^	Yes
“Wiggle” browser tracks	Yes	Yes^5^	No
Gene-level read counts for DESeq, edgeR	Yes	Partial	No
Exon-level read counts for DEXSeq	Yes	No	No
Splice Junction Metrics			
# Distinct Junction Loci, Known/Novel, High/Low coverage	Yes	Partial^5^	No
# Splice Junction Events, Known/Novel, High/Low coverage loci	Yes	Partial^5^	No
Splice junction coverage “.bed” browser tracks	Yes	No	No
Coverage read-pair counts for all Junction Loci	Yes	No	No
Visualization and Cross-Comparison			
Cross-Comparison between replicates	Yes	Partial^6^	Partial^6^
Contrast by lane/run, biological group, etc.	Yes	No	No
Generate Multiplots (png, svg, etc.)	Yes	No	No
Generate QC reports (pdf)	Yes	No	No

RSeQC functions with documented flaws are marked with an asterisk (^*^); see the Additional file 2 for more information. (Note: ^1^Does not separately track read-pairs for paired-end data. ^2^Performs analysis on a subsample of input reads. ^3^Only calculates mean and standard deviation. ^4^Only profiles some cigar operations. ^5^No paired-end mode, may double-count overlapping paired reads. ^6^Generates comparison plots only for some metrics.)

The RNA-SeQC software package lacks many vital quality control metrics [8]. It does not calculate nucleotide-by-cycle, “N”-rate by cycle, insert size distribution, clipping profile, cigar profile, or any splice-junction-related statistics. While it may be sufficient for some purposes, the absence of these critical QC statistics may allow biases, artifacts, or errors to go undetected.

The RSeQC software package, which ostensibly features a number of the functions implemented in QoRTs, possesses numerous systematic bugs and flaws that cause it to consistently produce erroneous and/or misleading results across several critical QC metrics [7]. For the purposes of internal testing we generated a variety of simple simulated SAM alignment files, each containing up to a dozen ten-base-pair reads. Both QoRTs (version 0.2.5, released March 5^th^, 2015) and RSeQC (version 2.6.1, current as of March 5^th^, 2015) were run on these example reads. Much of the resultant QC data generated by RSeQC was found to be inaccurate. Documentation of a subset of these inconsistencies is provided in the supplementary materials (see Additional file 2). Many of these inaccuracies could potentially serve to obfuscate real quality control issues or falsely suggest the presence of nonexistent issues. The fact that such numerous and fundamental errors remain present in a fully mature two-year-old software tool demonstrates that RSeQC has not been subject to sufficient testing.

In addition, both RSeQC and RNA-SeQC only provide very limited tools for visual cross-comparison between replicates. The few cross-comparison plots that are available simply plot all replicates over the same plotting area, each in a different color. QoRTs can generate plots that contrast and differentiate groups of replicates, allowing easy identification of systematic biases or errors.

## Conclusions

The QoRTs software package is a powerful, efficient, and convenient multifunction toolkit capable of facilitating quality control, data visualization, and data processing. It quickly and efficiently generates numerous QC metrics and provides tools for cross-comparison of samples by batch or group, greatly simplifying the identification of outliers and of phenodata-associated patterns.

In addition, QoRTs reproduces and/or improves upon the data processing functionality provided by numerous other disparate tools such as the samtools bam2wig tool, the DEXSeq count tool, and the HTSeq-count tool [22, 26, 27, 31]. These functions, along with the generation of the QC metrics, can be executed as part of a single unified data-processing/quality-control run, greatly reducing both the complexity and the total runtime of the analysis pipeline.

## Availability and requirements

- **Project name**: QoRTs
- **Project home page**: http://hartleys.github.io/QoRTs/index.html
- **Operating system(s)**: Platform independent
- **Programming language**: R, Java/Scala
- **Other requirements**: Java 1.6 or higher (64-bit), R 3.0.2 or higher.
- **License**: This software is “United States Government Work” under the terms of the United States Copyright Act. It was written as part of the authors’ official duties for the United States Government and thus cannot be copyrighted. This software is freely available to the public for use without a copyright notice. Restrictions cannot be placed on its present or future use.

## Additional files

Additional file 1:
**The QoRTs package vignette.**
Additional file 2:
**Documentation of some of the errors and flaws found with the RSeQC package.**

## Abbreviations

QC: Quality control
QoRTs: Quality of RNA-Seq Toolset
RNA-Seq: Next-generation RNA sequencing
