# Supplementary material for: QoRTs: a comprehensive toolset for quality control and data processing of RNA-Seq experiments
Source: BMC Bioinformatics. 2015 Jul 19;16(1):224. doi: 10.1186/s12859-015-0670-5 (PMC4506620; doi:10.1186/s12859-015-0670-5)
Supplement: Additional file 1: — The QoRTs package vignette. [file 12859_2015_670_MOESM1_ESM.pdf]

QoRTs Package User Manual

Stephen Hartley
National Human Genome Research Institute
National Institutes of Health
v0.3.5

May 22, 2015

Contents

1 Overview **3**

2 Requirements **4**
2.1 Recommendations 4

3 Preparations **5**
3.1 Alignment 5
3.2 Sorting 5

4 Quick Start **6**

5 Dataset Organization **6**
5.1 Example data 7

6 Processing of aligned RNA-Seq data **8**
6.1 Memory Usage 10

7 Visualization **10**
7.1 Reading the QC data into R 11
7.2 Generating all default plots 11
7.3 Plotting by sample, lane, or group 12
7.3.1 Summary Plots 12
7.3.2 Colored by Sample 15
7.3.3 Colored by Lane/Batch 16
7.3.4 Colored by Group/Phenotype 17
7.3.5 Basic Sample Highlight 18
7.3.6 Sample Highlight, Colored by Lane 19
7.4 Description of Individual Plots 20

7.4.1 Phred Quality Score . . . 21
7.4.2 GC Content . . . 22
7.4.3 Clipping Profile . . . 23
7.4.4 Cigar Op Profile . . . 24
7.4.5 Cigar Length Distribution . . . 25
7.4.6 Insert Size . . . 26
7.4.7 N-Rate . . . 27
7.4.8 Gene-Body Coverage . . . 28
7.4.9 Cumulative Gene Diversity . . . 29
7.4.10 Nucleotide Rates, by Cycle . . . 30
7.4.11 Aligned Nucleotide Rates, by Cycle . . . 31
7.4.12 Leading Clipped Nucleotide Rates . . . 32
7.4.13 Trailing Clipped Nucleotide Rates . . . 33
7.4.14 Mapping location rates . . . 34
7.4.15 Splice Junction Loci . . . 35
7.4.16 Number of Splice Junction Events . . . 36
7.4.17 Splice Junction Event Rates per Read-Pair . . . 37
7.4.18 Breakdown of Splice Junction Events . . . 38
7.4.19 Breakdown of Splice Junction Events, by locus type . . . 39
7.4.20 Strandedness test . . . 40
7.4.21 Mapping stats . . . 41
7.4.22 Chromosome counts . . . 42
7.4.23 Normalization Factors . . . 43
7.4.24 Normalization Factor Ratio . . . 44
7.4.25 Read drop rate . . . 45

8 Identifying Problems 46
8.1 Example 1: Sequencer Hiccup . . . 47
8.2 Example 2: Badly Degraded RNA . . . 48

9 Secondary Utilities 50
9.1 Generating a flattened annotation file . . . 50
9.2 Merging Count Data . . . 50
9.3 Generating genome browser tracks . . . 51
9.3.1 Generating wiggle tracks . . . 51
9.3.2 Merging wiggle tracks . . . 53
9.3.3 Generating splice-junction tracks . . . 53
9.3.4 Merging splice-junction tracks . . . 54
9.4 Importing data into other tools . . . 55
9.4.1 DEXSeq compatibility . . . 55

10 Session Information 56

11 Legal 57

1 Overview

The QoRTs software package is a fast, efficient, and portable multifunction toolkit designed to assist in the analysis, quality control, and data management of RNA-Seq datasets. Its primary function is to aid in the detection and identification of errors, biases, and artifacts produced by paired-end high-throughput RNA-Seq technology. In addition, it can produce count data designed for use with differential expression [1] and differential exon usage tools [2], as well as individual-sample and/or group-summary genome track files suitable for use with the UCSC genome browser (or any compatible browser).

In its primary role as a QC tool it can produce a wide variety of graphs, plots, and tables that allow the data to be visualized in various ways. Data can be compiled and contrasted in multiple ways to allow systematic errors or artifacts to reveal themselves more easily. While it will not directly assign pass/fail status, it is a powerful tool for bioinformaticians to detect and identify features in the data. In (hopefully) most cases, these plots and graphs will not reveal anything other than mixed statistical noise. Next-Gen sequencing technologies have matured to the point where gross systematic errors and batch-specific biases are relatively modest and rare. However: mistakes can still occur, and basing conclusions on flawed data can be disastrous.

Across the field of bioinformatics there are numerous cases where biases, artifacts, and other data quality or bioinformatic issues have called results into question, sometimes resulting in retractions. In many of these cases the problems were only identified after the study came under intense scrutiny when the results were interesting and/or contentious, and the specific issues at fault were generally not well-characterized until afterwards. The primary purpose of QoRTs is to cast a wide net, characterizing the data in as many ways as is feasible so that quality issues that would otherwise be obscured can be recognized and dealt with, even if these issues have not been previously encountered.

The QoRTs package is composed of two parts: a java jar-file (for data processing) and a companion R package (for generating tables, figures, and plots). The java utility is written in the Scala programming language (v2.11.1), however, it has been compiled to java byte-code and does not require an installation of Scala (or any other external libraries) in order to function. The entire QoRTs toolkit can be used on almost any operating system that supports java and R. While not explicitly required, the use of a 64-bit version of java is recommended.

This vignette primarily covers the quality control functionality of QoRTs, and briefly covers the other functions and capabilities.

The most recent release of QoRTs is available on the QoRTs github page (`http://github.com/hartleys/QoRTs`). Additional help and documentation is available online (`http://hartleys.github.io/QoRTs/index.html`). A comprehensive walkthrough covering the entire process from post-alignment all the way to differential expression analysis, along with a full example dataset and example output can be found online (`https://dl.dropboxusercontent.com/u/103621176/qorts/exData/QoRTsFullExampleData.zip`).

[1] Such as *DESeq*, *DESeq2* [1] or *edgeR* [2]
[2] Such as *DEXSeq* [3]

2 Requirements

Hardware: The java utility performs the bulk of the data processing, and will generally require at least 4gb of RAM. In general at least 8gb is recommended if available. The R package is only responsible for some light data processing and for plotting/visualization, and thus has much lower resource requirements. It should run adequately on any reasonably-powerful workstation.

Software: The QoRTs software package requires R version 3.0.2 or higher, as well as java 6 or higher. It is strongly recommended that a 64-bit version of java be used, as the 32-bit versions generally cannot allocate sufficient RAM.

Annotation: QoRTs requires transcript annotations in the form of a gtf file. If you are using a annotation-guided aligner (which is STRONGLY recommended) it is likely you already have a transcript gtf file for your reference genome. We recommend you use the same annotation gtf for alignment, QC, and downstream analysis. We have found the Ensembl "Gene Sets" gtf[3] suitable for these purposes. However, any format that adheres to the gtf file specification[4] will work.

Dataset: QoRTs requires aligned RNA-Seq data. Data can be paired-end or single-end, unstranded or stranded (using either strandedness rule, see Section 6). It is strongly recommended, but not explicitly required, that the SAM/BAM files be sorted (either by name or position). QoRTs can use additional metadata (such as technical replicate status, case/control status, batch id, etc) to produce comparisons between these replicate groups, but this information is optional.

2.1 Recommendations

Clipping: For the purposes of Quality Control, it is generally best if reads are NOT hard-clipped prior to alignment. This is because hard clipping, espeically variable hard-clipping from both the start and end of reads, makes it impossible to determine sequencer cycle from the aligned bam files, which in turn can obfuscate cycle specific artifacts, biases, errors, and effects. If undesired sequence must be removed, it is generally preferred to replace such nucleotides with N's, as this preserves cycle information. Note that many advanced RNA-Seq aligners will "soft clip" nonmatching sequence that occurs on the read ends, so hard-clipping low quality sequence is generally unnessessary and may reduce mapping rate and accuracy.

Replicates: Using barcoding, it is possible to build a combined library of multiple distinct samples which can be run together on the sequencing machine and then demultiplexed afterward. In general, it is recommended that samples for a particular study be multiplexed and merged into "balanced" combined libraries, each containing equal numbers of each biological condition. If necessary, these combined libraries can be run across multiple sequencer lanes or runs to achieve the desired read depth on each sample.

[3]Which can be acquired from the Ensembl website at http://www.ensembl.org

[4]See the gtf file specification at http://genome.ucsc.edu/FAQ/FAQformat.html

3 Preparations

There are a number of processing steps that must occur prior to the creation of usable bam files. We will briefly go over the required steps here:

3.1 Alignment

QoRTs is designed to run on paired-ended or single-ended next-gen RNA-Seq data. The data must be aligned (or "mapped") to a reference genome before QoRTs can be run. RNA-Star [4], GSNAP [5], and TopHat2 [6] are all popular and effective aligners for use with RNA-Seq data. The use of short-read or unspliced aligners such as BowTie, ELAND, BWA, or Novoalign is NOT recommended.

3.2 Sorting

For single-end data, the reads can be in any order, and sorting is unnecessary.

For paired-end data, QoRTs is designed to automatically accept files sorted by either read-name or position. Sorting can be accomplished via the samtools or novosort tools (which are NOT included with QoRTs). Sorting is unnecessary for single-end data.

To sort by coordinate:

```
samtools sort unsorted.bam sorted
OR
novosort unsorted.bam > sorted.bam
```

Or, to sort by read name:

```
samtools sort -n unsorted.bam sortedByName
OR
novosort -n unsorted.bam > sortedByName.bam
```

Running in the default mode, QoRTs will accept both name-sorted and position-sorted BAM files. Technically QoRTs can accept any BAM files regardless of ordering; however, if a large number of paired mates are not located near one another in the file then memory usage may be too high as QoRTs stores unmatched mates in memory.

QoRTs also has a separate mode designed only for name-sorted samples, which can be activated using the "`--nameSorted`" option. Under certain conditions this may improve speed and reduce memory usage. Under typical conditions any improvement is trivial.

4 Quick Start

In order to produce quality control metrics, plots, and pdf reports on a single replicate, simply use the command:

```
java -Xmx4G -jar /path/to/jarfile/QoRTs.jar QC \
                   --generatePlots  \
                   mybamfile.bam  \
                   transcriptAnnotationFile.gtf.gz  \
                   /qc/data/dir/path/
```

Note: This command must be executed as a single line. Additional options may be required depending on the nature of the dataset. For single-ended data, the `--singleEnded` option should be included. For strand-specific data, the `--stranded` option should be included, and possibly also the `--fr_secondStrand` option depending on the stranded library type. For position-sorted data, the `--coordSorted` option should be used. See 6, or the QC utility documentation[5] for more information on the available options.

5 Dataset Organization

Several QoRTs functions will require "decoder" information in some form, which describes each sample and all of its technical replicates (if any). The simplest method is to provide a decoder file. All of the columns are optional except for unique.ID, however if group, lane, and/or technical replicate information is not supplied then QoRTs obviously will not be able to produce plots that organized and/or grouped by these factors.

Fields:

- *unique.ID*: A unique identifier for the row. QoRTs will also accept the synonym "lanebam.ID". THIS IS THE ONLY MANDATORY FIELD.
- *lane.ID*: The ID of the lane or batch. By default this will be set to "UNKNOWN".
- *group.ID*: The ID of the "group". For example: "Case" or "Control". By default this will be set to "UNKNOWN".
- *sample.ID*: The ID of the biological sample from which the data originated. Each sample can have multiple rows, representing technical replicates (in which the same sample is sequenced on multiple lanes or runs). By default QoRTs will assume that every row comes from a separate sample, and will thus set the sample.ID to equal the unique.ID.
- *qc.data.dir*: The directory in which the java utility saved all the output data. If this column does not exist, by default it will be set to be unique.ID.
- *input.read.pair.count*: (Optional) The number of reads in the original fastq file, prior to alignment. If this field is left out, then QoRTs will skip comparisons and plotting of mapping rates. There are a number of other ways to input this value. See Section 7.4.21.

[5] Found here.

- *multi.mapped.read.pair.count*: (Optional) The number of reads that were multi-mapped by the aligner. This field should only be used if filtering for multi-mapped reads is performed prior to analysis with QoRTs (which is not recommended). Even in this case, this field can simply be left out and QoRTs will skip plotting and comparisons of multi-mapping rates. See Section 7.4.21.

In addition, the decoder can contain any other additional columns as desired, as long as all of the column names are distinct.

While QoRTs is primarily designed to allow comparisons between biological groups, lanes, sequencing-runs, etc, it can also be used on simpler datasets, or even individual samples. Thus, only the unique.ID variable is actually required. For testing purposes, you can produce a completed decoder (with all default values filled in) using the `completeAndCheckDecoder` function.

The simplest example would just be a character vector of unique.ID's:

```
completeAndCheckDecoder(c("SAMPLE1","SAMPLE2","SAMPLE3"));
```

```
##   unique.ID sample.ID lane.ID group.ID qc.data.dir
## 1   SAMPLE1   SAMPLE1 UNKNOWN  UNKNOWN     SAMPLE1
## 2   SAMPLE2   SAMPLE2 UNKNOWN  UNKNOWN     SAMPLE2
## 3   SAMPLE3   SAMPLE3 UNKNOWN  UNKNOWN     SAMPLE3
```

Alternatively, any of the optional fields can be included or left out, as desired:

```
incompleteDecoder <- data.frame(unique.ID = c("SAMPLE1", "SAMPLE2"),
                                group.ID = c("CASE","CONTROL"));
completeAndCheckDecoder(incompleteDecoder);
```

```
##   unique.ID group.ID sample.ID lane.ID qc.data.dir
## 1   SAMPLE1     CASE   SAMPLE1 UNKNOWN     SAMPLE1
## 2   SAMPLE2  CONTROL   SAMPLE2 UNKNOWN     SAMPLE2
```

5.1 Example data

The separate R package *QoRTsExampleData* contains an example dataset with an example decoder:

```
directory <- system.file("extdata/", package="QoRTsExampleData",
                         mustWork=TRUE);
decoder.file <- system.file("extdata/decoder.txt",
                            package="QoRTsExampleData",
                            mustWork=TRUE);
decoder.data <- read.table(decoder.file,
                           header=T,
                           stringsAsFactors=F);
print(decoder.data);
```

```
##    sample.ID lane.ID unique.ID  qc.data.dir group.ID input.read.pair.count
## 1      SAMP1      L1 SAMP1_RG1 ex/SAMP1_RG1     CASE                465298
## 2      SAMP1      L2 SAMP1_RG2 ex/SAMP1_RG2     CASE                472241
```

```
## 3      SAMP1      L3 SAMP1_RG3 ex/SAMP1_RG3     CASE                  500691
## 4      SAMP2      L1 SAMP2_RG1 ex/SAMP2_RG1     CASE                  461405
## 5      SAMP2      L2 SAMP2_RG2 ex/SAMP2_RG2     CASE                  467713
## 6      SAMP2      L3 SAMP2_RG3 ex/SAMP2_RG3     CASE                  492322
## 7      SAMP3      L1 SAMP3_RG1 ex/SAMP3_RG1     CASE                  485397
## 8      SAMP3      L2 SAMP3_RG2 ex/SAMP3_RG2     CASE                  489859
## 9      SAMP3      L3 SAMP3_RG3 ex/SAMP3_RG3     CASE                  516906
## 10     SAMP4      L1 SAMP4_RG1 ex/SAMP4_RG1     CTRL                  460968
## 11     SAMP4      L2 SAMP4_RG2 ex/SAMP4_RG2     CTRL                  468391
## 12     SAMP4      L3 SAMP4_RG3 ex/SAMP4_RG3     CTRL                  484530
## 13     SAMP5      L1 SAMP5_RG1 ex/SAMP5_RG1     CTRL                  469884
## 14     SAMP5      L2 SAMP5_RG2 ex/SAMP5_RG2     CTRL                  475001
## 15     SAMP5      L3 SAMP5_RG3 ex/SAMP5_RG3     CTRL                  494213
## 16     SAMP6      L1 SAMP6_RG1 ex/SAMP6_RG1     CTRL                  452429
## 17     SAMP6      L2 SAMP6_RG2 ex/SAMP6_RG2     CTRL                  458810
## 18     SAMP6      L3 SAMP6_RG3 ex/SAMP6_RG3     CTRL                  477751
```

Due to size constraints the example dataset contained in this R package includes only the QC output data, not the raw bam-files themselves. The actual bamfiles, along with a step-by-step example walkthrough that covers the entire analysis pipeline, are linked to from the QoRTs github website (https://github.com/hartleys/QoRTs).

The example dataset is derived from a set of rat pineal gland samples, which were multiplexed and sequenced across six sequencer lanes. For the sake of simplicity, the example dataset was limited to only six samples and three lanes. However, the bam files alone would still occupy 18 gigabytes of disk space, which would make it unsuitable for distribution as an example dataset. To further reduce the example bamfile sizes, only reads that mapped to chromosomes chr14, chr15, chrX, and chrM were included. Additionally, all the selected chromosomes EXCEPT for chromosome 14 were randomly downsampled to 30 percent of their original read counts.

THIS DATASET IS INTENDED FOR DEMONSTRATION AND TESTING PURPOSES ONLY. Due to the various alterations that have been made to reduce file sizes and improve portability, it is not representitive of the original data and as such is really not suitable for any actual analyses.

6 Processing of aligned RNA-Seq data

The first step is to process the aligned RNA-Seq data. The bulk of the data-processing is performed by the QoRTs.jar java utility. This tool produces an array of output files, analyzing and tabulating the data in various ways. This utility requires about 10-20gb of RAM for most genomes, and takes roughly 4-7 minutes to process 1 million read-pairs.

```
java -jar /path/to/jarfile/QoRTs.jar QC \
                   mybamfile.bam \
                   transcriptAnnotationFile.gtf.gz \
```

```
                    /qc/data/dir/path/
```

In the above command (which must be entered as a single line), you must replace /path/to/jarfile/ with the file-path to the directory in which the jar file is kept. The path /qc/data/dir/path/ should be replaced with the path you want the QC data to be written. This should match the path located in the decoder in the qc.data.dir column for this sample-run.

The bam processing tool includes numerous options. A full description of these options can be found in the online documentation of the jar utility[6], or by entering the command:

```
java -jar /path/to/jarfile/QoRTs.jar QC --man
```

There are a number of crucial points that require attention when using the QoRTs.jar QC command.

- *Stranded Data:* By default, QoRTs assumes that the data is *NOT* strand-specific. For strand-specific data, the --stranded option must be used.
- *Stranded Library Type:* The --fr_secondStrand option may be required depending on the stranded library type. QoRTs does not attempt to automatically detect the platform and protocol used for stranded data. There are two types of strand-specific protocols, which are described by the TopHat/CuffLinks documentation at http://cufflinks.cbcb.umd.edu/manual.html#library as fr-firststrand and fr-secondstrand. In HTSeq, these same library type options are defined as -s reverse and -s yes respectively. According to the CuffLinks manual, fr-firststrand (the default used by QoRTs for stranded data) applies to dUTP, NSR, and NNSR protocols, whereas fr-secondstrand applies to "Directional Illumina (ligation)" and "Standard SOLiD" protocols. If you are unsure which library type applies to your dataset, don't worry: one of the tests will report stranded library type. If you use this test to determine library type, be aware that you may have to re-run QoRTs with the correct library type set.
- *Read Groups:* Depending on the production pipeline, each biological sample may be run across multiple sequencer lanes. These seperate files can be merged together either before or after analysis with QoRTs (and maybe even before alignment). However, if the merger occurs before analysis with QoRTs, then each bam file will consist of multiple seperate lanes or runs. In this case, it is STRONGLY recommended that seperate QC runs be performed on each "read group", using the --readGroup option. This will prevent run- or lane-specific biases, artifacts, or errors from being obfuscated.
- *Read Sorting:* For paired-end data reads must be sorted. By default, QoRTs can accept files sorted by name OR by position.
- *Single-end vs paired-end:* By default, QoRTs assumes the input bam file consists of paired-end data. For single-end data, the --isSingleEnd option must be used.

For example, to read the first read group bam-file for SAMP1 from the example dataset (which is stranded, coordinate-sorted, and uses the fr_firstStrand stranded library type), one would use the following command:

```
java -jar /path/to/jarfile/QoRTs.jar QC \
                    --stranded \
                    inputData/bamFiles/SAMP1_RG1.bam \
                    inputData/annoFiles/anno.gtf.gz \
```

[6] Found here: http://hartleys.github.io/QoRTs/jarHtml/index.html

```
                    outputData/qortsData/SAMP1_RG1/
```

This command must be run on each bam file (and possibly more than once on each, if each bam file consists of multiple separate read-groups).

6.1 Memory Usage

Memory usage: The QoRTs QC utility requires at least 4gb or RAM for most genomes / datasets. Larger genomes, genomes with more annotated genes/transcripts, or larger bam files may require more RAM. You can set the maximum amount of RAM allocated to the JVM using the options -Xmx4000M. This should be included *before* the -jar in the command line. For example:

```
#Set the maximum to the minimum recommended 4 gigabytes:
java -Xmx4000M -jar /path/to/jarfile/QoRTs.jar QC \
                   --stranded \
                   inputData/bamFiles/SAMP1_RG1.bam \
                   inputData/annoFiles/anno.gtf.gz \
                   outputData/qortsData/SAMP1_RG1/

#Or Set the maximum to 16 gigabytes:
java -Xmx16G -jar /path/to/jarfile/QoRTs.jar QC \
                   --stranded \
                   inputData/bamFiles/SAMP1_RG1.bam \
                   inputData/annoFiles/anno.gtf.gz \
                   outputData/qortsData/SAMP1_RG1/
```

This option can be used with any and all of the QoRTs java utilities.

7 Visualization

All visualization is performed the the QoRTs companion R package.

For basic QC analyses it is often not necessary to write any R code, as QoRTs comes with a simple R script that generates a standard set of png multiplots, pdf reports, and a large tab-delimited summary table. The qortsGenMultiQC.R script should be included in the scripts directory of the main package archive. This script can be run using the command:

```
Rscript qortsGenMultiQC.R infile/dir/ decoderFile.txt outfile/dir/
```

infile/dir/ should be the parent directory within which all the QC output data is contained. decoderFile.txt should be the decoder file, as described in Section 5. outfile/dir/ should be the directory where all output plots will be placed.

Alternatively, custom R code can be used to generate non-standard plots or multiplots, alter plotting parameters, or to generate plots interactively. In addition to the documentation provided in the rest

of this section, the full R docs can be found online. See the github page for a link to the complete documentation.

7.1 Reading the QC data into R

First you must read in all the QC output from the java utility, using the command below. This command requires 2 arguments: a root directory and a decoder (which can be either a data frame or a file). We will be using the example data found in package *QoRTexampleData*, which is described in Section 5.1.

```
res <- read.qc.results.data(directory, decoder = decoder.data,
                            calc.DESeq2 = TRUE, calc.edgeR = TRUE);
```

Note that the `calc.DESeq2` and `calc.edgeR` options are optional, and tell QoRTs to attempt to load the *DESeq2* and *edgeR* packages (respectively) and use the packages to calculate additional normalization size factors. This is not strictly needed for most purposes, but allows QoRTs to plot the normalization factors against one another. See section 7.4.23 for more information.

7.2 Generating all default plots

To generate all the default compiled plots all at once, use the command:

```
makeMultiPlot.all(res, outfile.dir = "./");
```

This will usually take some time to run, but will produce all the compiled summary plots described in the rest of this section, including separate highlight plots for every sample in the dataset. By default all images will saved to file as pngs. There are a number of alternatives, which can be selected using the `plot.device.name` parameter. For example:

```
#Generate multi-page pdf reports:
makeMultiPlot.all(res, outfile.dir = "./", plot.device.name = "pdf");
#Generate svg vector drawings:
makeMultiPlot.all(res, outfile.dir = "./", plot.device.name = "svg");
```

Note: The R PDF device primarily uses vector drawings, however, some of the plots are too large to be efficiently stored as vectors. If pdf reports are desired, we recommend installing the *png* package. If this package is installed, then QoRTs will automatically rasterize the plotting areas of certain large plots (in particular: the gene diversity plots and the various NVC plots). Setting the `rasterize.large.plots` parameter to `FALSE` will deactivate this behavior. The `raster.height` and `raster.width` parameters can be used to increase the pixel resolution of the rasterized plotting regions, if desired.

The *png* package can be installed with the R command:

```
install.packages("png");
```

7.3 Plotting by sample, lane, or group

QoRTs includes automated methods for organizing and plotting the results in numerous different ways. The intent of these tools is to make any patterns and biases more visible to the user.

All plotting functions in QoRTs require a `QoRTs_Plotter` object. A `QoRTs_Plotter` is a `RefClass` object that contains all the QC data along with a set of parameters that determine how to color and draw each replicate's data. A full accounting of all possible options available in the is beyond the scope of this manual, but can be found in the help docs for the `QoRTs_Plotter` class.

7.3.1 Summary Plots

The most basic `QoRTs_Plotter` can be created using the command:

```
basic.plotter <- build.plotter.basic(res);
```

This `QoRTs_Plotter` object can be used to plot all replicates on top of one another in semi-transparent blue. For example:

```
makePlot.insert.size(basic.plotter);
```

Which produces Figure 1:

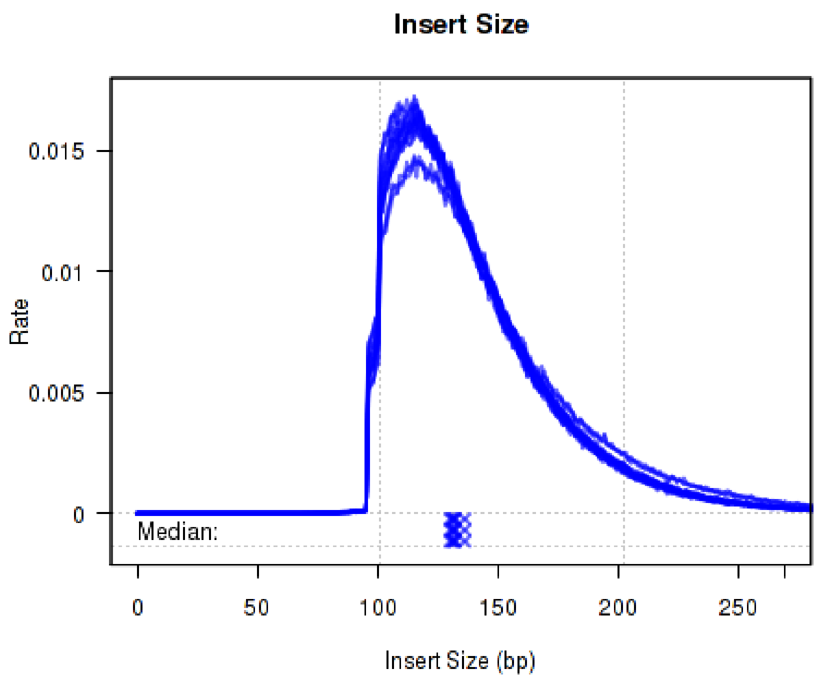

Figure 1: Phred Quality Score Plots

The above example plot displays the "Insert Size" of each replicate, as described in Section 7.4.6.

In addition, a compiled multi-plot in this style, containing all the standard QC plots, can be generated with the command:

```
makeMultiPlot.basic(res);
```

Which produces Figure 2:

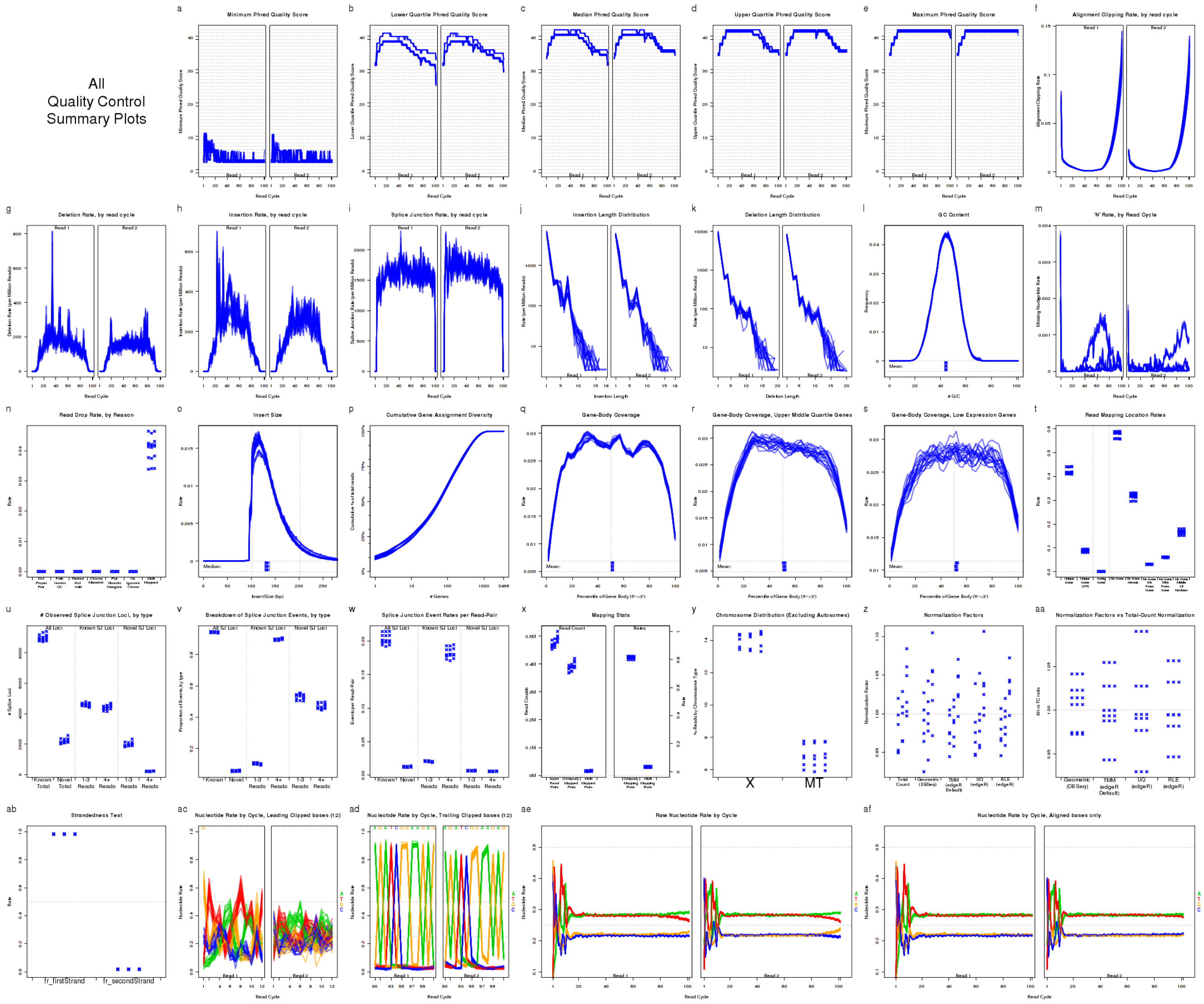

Figure 2: Compiled summary multi-plot

This plot includes many sub-plots, all in a single frame. The sub-plots are:

- *(a)*: Minimum phred quality score, by read position. Described in section 7.4.1
- *(b)*: Lower-quartile phred quality score, by read position. Described in section 7.4.1
- *(c)*: Median phred quality score, by read position. Described in section 7.4.1
- *(d)*: Upper-quartile phred quality score, by read position. Described in section 7.4.1
- *(e)*: Maximum phred quality score, by read position. Described in section 7.4.1
- *(f)*: Clipping profile. Described in section 7.4.3

- *(g)*: Deletion profile. Described in section 7.4.4
- *(h)*: Insertion profile. Described in section 7.4.4
- *(i)*: Splicing profile. Described in section 7.4.4
- *(j)*: Insertion length distribution. Described in section 7.4.5
- *(k)*: Deletion length distribution. Described in section 7.4.5
- *(l)*: GC content distribution. Described in section 7.4.2
- *(m)*: N-rate, by read position. Described in section 7.4.7
- *(n)*: Read drop rate. Described in section 7.4.25
- *(o)*: Insert size distribution. Described in section 7.4.6
- *(p)*: Cumulative gene assignment diversity. Described in section 7.4.9
- *(q)*: Gene body coverage, overall. Described in section 7.4.8
- *(r)*: Gene body coverage, upper-middle quartile genes. Described in section 7.4.8
- *(s)*: Gene body coverage, low expression genes. Described in section 7.4.8
- *(t)*: Read mapping location rates. Described in section 7.4.14
- *(u)*: Observed splice junction loci counts. Described in section 7.4.15
- *(v)*: Splice junction event distribution. Described in section 7.4.17
- *(w)*: Splice junction events per read-pair. Described in section 7.4.19
- *(x)*: Read-mapping statistics. Described in section 7.4.21
- *(y)*: Chromosome counts. Described in section 7.4.22
- *(z)*: Comparison of normalization factors. Described in section 7.4.23
- *(aa)*: Comparison of normalization factors relative to TC normalization. Described in section 7.4.24
- *(ab)*: Strandedness test. Described in section 7.4.20
- *(ac)*: Leading-clipped nucleotide rates. Described in section 7.4.12
- *(ad)*: Trailing-clipped nucleotide rates. Described in section 7.4.13
- *(ae)*: Raw nucleotide rate by read position. Described in section 7.4.10
- *(af)*: Aligned nucleotide rate by read position. Described in section 7.4.11

A printable pdf version of this multi-plot, with 6 plots on each page, can be generated with using the options:

```
makeMultiPlot.basic(res, plot.device.name = "pdf");
```

7.3.2 Colored by Sample

For small datasets, it can be useful to simply color each sample a distinct color, so that outliers can be easily identified. For this, you first generate a `QoRTs_Plotter` using the command:

```
bySample.plotter <- build.plotter.colorBySample(res);
```

This `QoRTs_Plotter` can be used to draw all the replicate on top of one another, but color them based on their sample.ID. The plotter can then be used to create various QC plots, for example:

```
makePlot.insert.size(bySample.plotter);
makePlot.legend.over("topright",bySample.plotter);
```

Which produces Figure 3:

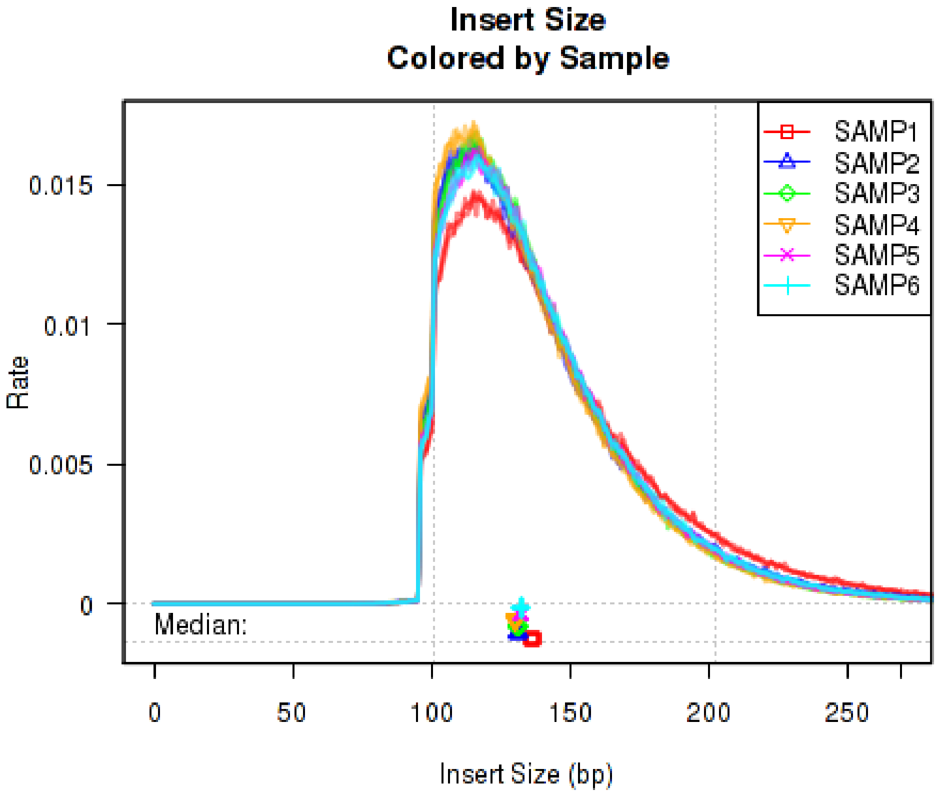

Figure 3: Phred Quality Score Plots

The above example plot displays the "Insert Size" of each replicate, as described in Section 7.4.6.

In addition, a compiled multi-plot in this style, containing all the standard QC plots, can be generated with the command:

```
makeMultiPlot.colorBySample(res);
```

7.3.3 Colored by Lane/Batch

In order to more easily detect batch effects, it is possible to color each replicate by lane/batch. For this, you can generate a QoRTs_Plotter with the command:

```
byLane.plotter <- build.plotter.colorByLane(res);
```

This QoRTs_Plotter can be used to color replicates based on lane.ID. The QoRTs_Plotter can then be used to create various QC plots, for example:

```
makePlot.insert.size(byLane.plotter);
makePlot.legend.over("topright",byLane.plotter);
```

Which produces Figure 4:

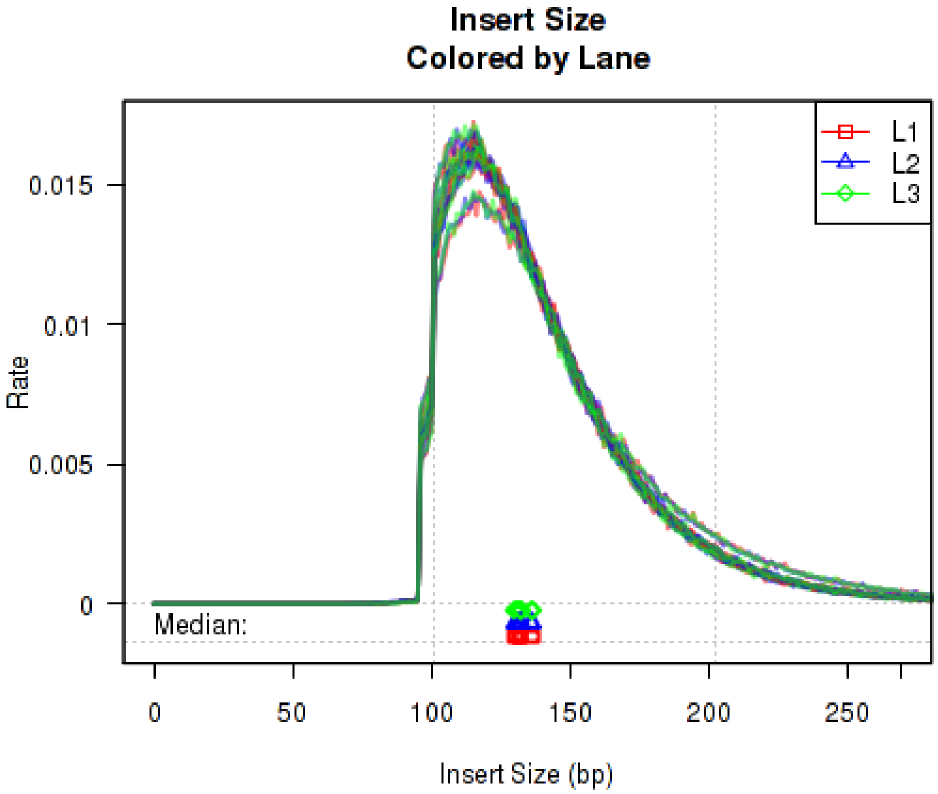

Figure 4: Phred Quality Score Plots

The above example plot displays the "Insert Size" of each replicate, as described in Section 7.4.6.

In addition, a compiled multi-plot in this style, containing all the standard QC plots, can be generated with the command:

```
makeMultiPlot.colorByLane(res);
```

7.3.4 Colored by Group/Phenotype

To detect variations caused by biological conditions (or artifacts and errors that occur disproportionately in certain biological conditions), it is sometimes useful to color samples by group.ID.

```
byGroup.plotter <- build.plotter.colorByGroup(res);
```

This `QoRTs_Plotter` can then be used to create various QC plots, for example:

```
makePlot.insert.size(byGroup.plotter);
makePlot.legend.over("topright",byGroup.plotter);
```

Which produces Figure 5:

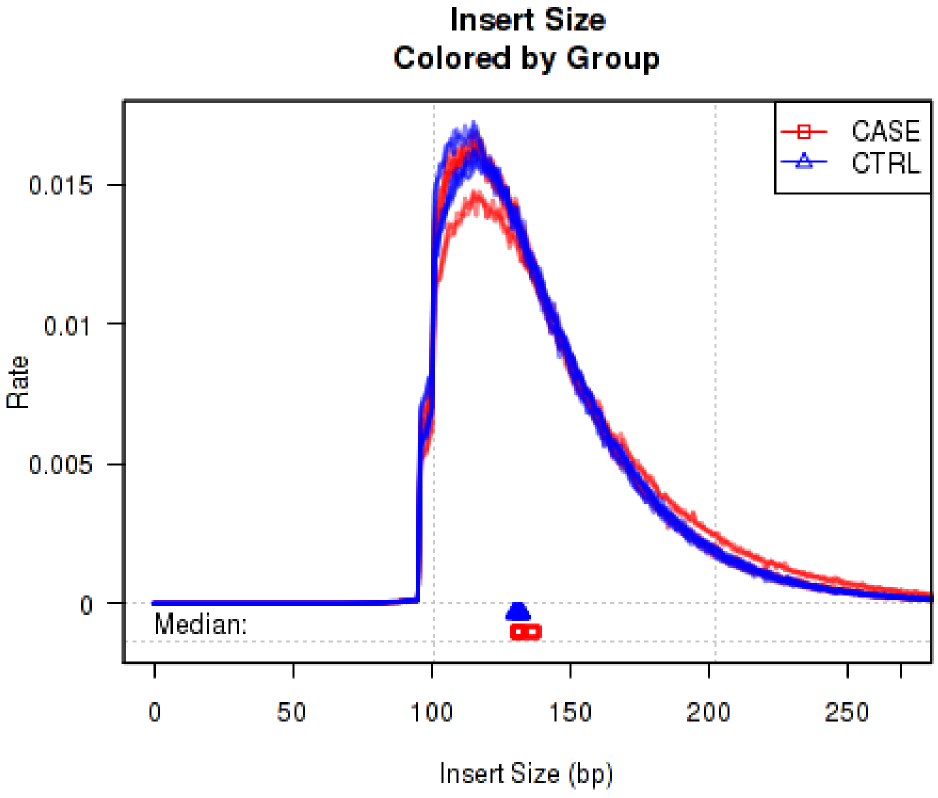

Figure 5: Phred Quality Score Plots

The above example plot displays the "Insert Size" of each replicate, as described in Section 7.4.6.

In addition, a compiled multi-plot in this style, containing all the standard QC plots, can be generated with the command:

```
makeMultiPlot.colorByGroup(res);
```

7.3.5 Basic Sample Highlight

Sometimes it is useful to "highlight" an individual sample.

```
sample.SAMP1.plotter <- build.plotter.highlightSample("SAMP1",res);
```

This QoRTs_Plotter can then be used to create various QC plots, for example:

```
makePlot.insert.size(sample.SAMP1.plotter);
makePlot.legend.over("topright",sample.SAMP1.plotter);
```

Which produces Figure 6:

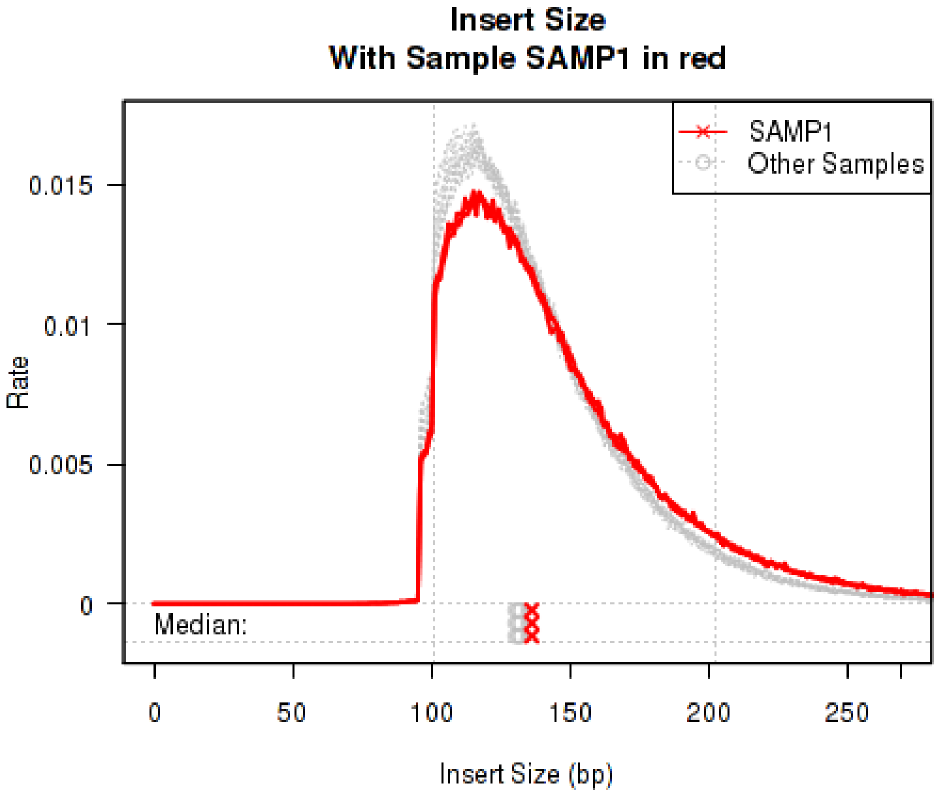

Figure 6: Phred Quality Score Plots

The above example plot displays the "Insert Size" of each replicate, as described in Section 7.4.6.

In addition, a compiled multi-plot in this style, containing all the standard QC plots, can be generated with the command:

```
makeMultiPlot.highlightSample(res,
                    curr.sample = "SAMP1");
```

7.3.6 Sample Highlight, Colored by Lane

Sometimes it can be useful to highlight an individual sample. However, if that sample has multiple "technical replicates" (derived from multiple separate lanes/runs on the same library), it can be useful to color the different runs with different distinct colors. With this plotter, only the "highlighted" sample is colored, all other samples are colored Gray.

```
sample.SAMP1.colorByLane.plotter <-
          build.plotter.highlightSample.colorByLane("SAMP1",res);
```

This `QoRTs_Plotter` can then be used to create various QC plots, for example:

```
makePlot.insert.size(sample.SAMP1.colorByLane.plotter);
makePlot.legend.over("topright",sample.SAMP1.colorByLane.plotter);
```

Which produces Figure 7:

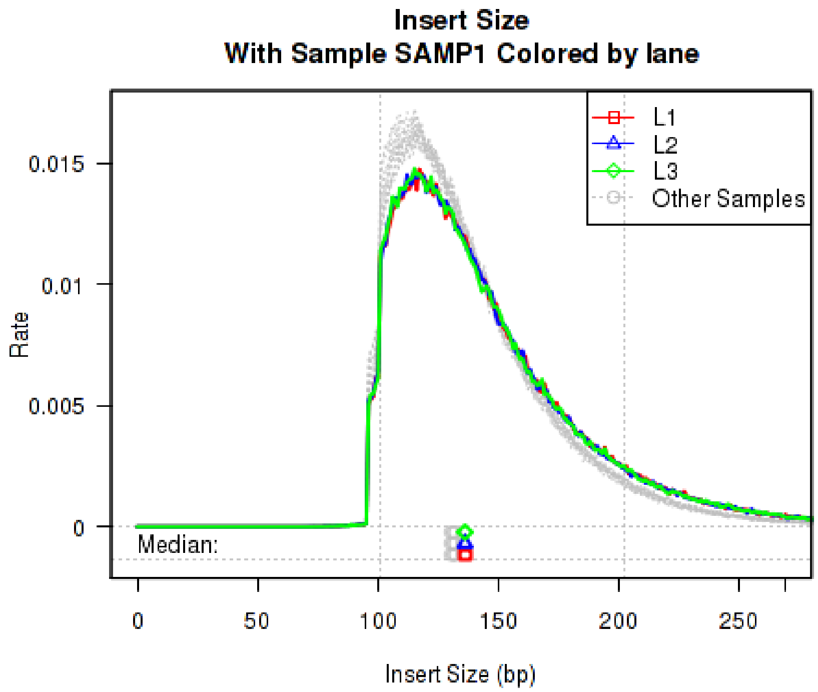

Figure 7: Phred Quality Score Plots

The above example plot displays the "Insert Size" of each replicate, as described in Section 7.4.6.

In addition, a compiled multi-plot in this style, containing all the standard QC plots, can be generated with the command:

```
makeMultiPlot.highlightSample.colorByLane(res,
                curr.sample = "SAMP1");
```

7.4 Description of Individual Plots

QoRTs is capable of producing a wide variety of different plots and graphs. While most of these plots will not be particularly interesting or informative in the majority of cases, they may reveal artifacts or errors if and when they occur.

The example plots in the following section all use the `byLane.plotter` `QoRTs_Plotter` (from Section 7.3.3), which colors each replicate by its lane ID.

What it means and what to look for:

In general, when examining these plots, users should scan for a number of potential anomalies:

- **"Spikes"**: In which one of the metrics jumps up or down abruptly, then returns to baseline.
- **"Shelves"**: In which one of the metrics jumps up or down abruptly, then continues at an increased or decreased level.
- **Outliers**: In which one or more particular samples or replicates are very different from all or most of the others. The presence of outliers may be an indicator for sample-collection, library-prep, or sequencing errors or artifacts.
- **Systematic Biases**: In which consistent differences appear between subsets of the data (eg. "lane.ID" or "group.ID"). Many of the biases measured by QoRTs are well-characterized, and many downstream analysis tools are robust against them when they are consistent and uniform. However: biases that vary disproportionately between sample groups may still drive false associations downstream.
- **Inconsistent samples**: In which the technical replicates of a specific biological sample shows substantial variation. In most studies, technical variation is very small relative to biological variation. In the example dataset (for example) the technical replicates are plotted almost on top of one another across many of the plots. If technical replicates do not cluster tightly, or if they cluster with the wrong replicates, then this may be an indicator of a sample swap.

Some "anomalous" metrics may be fundamental to the dataset and may not be indicative of any quality issues. For example, when profiling two different cell types one would expect the two groups to have very different profiles across a number of metrics. However: a single sample that is wildly different from the others within the same group may be cause for concern. In many cases variations may be observed across multiple metrics, all driven by the same underlying phenomenon. The breadth and depth of the metrics provided are intended to provide the tools necessary to identify the most likely underlying source(s) of the aberration(s).

Some aberrations may not be relevant to the study analysis even when they are representative of a real data quality issue. A moderate increase in the deletion rate may not have a noticable impact on expression quantification in a simple differential expression study. The same issue, however, might be catastrophic in a study focused on quantifying the rate of RNA transcription errors or RNA editing events. The number of combinations of study design, sample set structure, sequencing technology, and observed quality control issues are myriad, and all of these factors would inform decisionmaking.

Ultimatiely, bioinformaticians must use their own judgement in deciding how to proceed when unexplained abnormalities are discovered in their dataset.

7.4.1 Phred Quality Score

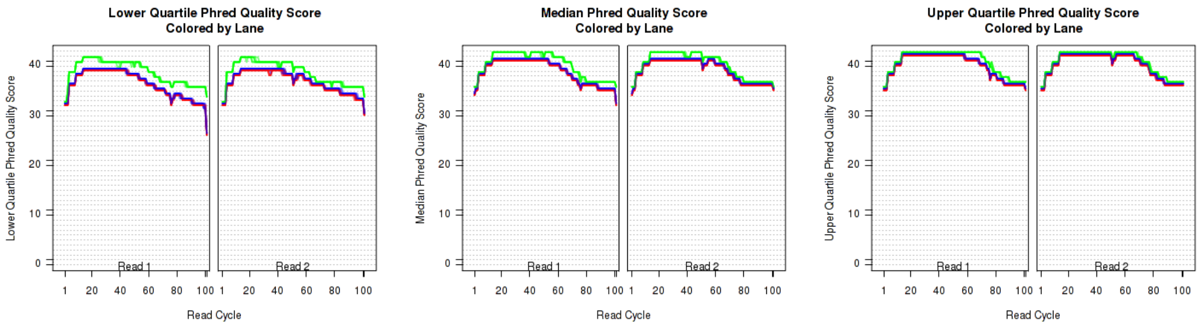

Figure 8: Phred Quality Score Plots

The plots shown in Figure 8 displays information about the phred quality score (y-axis) as a function of the position in the read (x-axis). Five statistics can be plotted: minimum, maximum, upper and lower quartiles, and median. These statistics are calculated individually for each replicate and each read position (ie, each plotted line corresponds to a replicate).

Note that the Phred score is always an integer, and as such these plots would normally be very difficult to read because lines would be plotted directly on top of one another. To reduce this problem, the plots are vertically offset from one another.

These plots can be generated individually with the commands:

```
makePlot.qual.pair(byLane.plotter,"lowerQuartile");
makePlot.qual.pair(byLane.plotter,"median");
makePlot.qual.pair(byLane.plotter,"upperQuartile");
```

Additional options (Not shown):

```
makePlot.qual.pair(byLane.plotter,"min");
makePlot.qual.pair(byLane.plotter,"max");
```

What it means and what to look for: These plots can be used to detect sequencer problems, bad lanes, or similar hardware-level artifacts and errors. Look for spikes or shelves, and ensure that the quality score is relatively consistent across samples and lanes, and that any differences that do exist are not disproportionate with respect to the study condition (or "group.ID").

7.4.2 GC Content

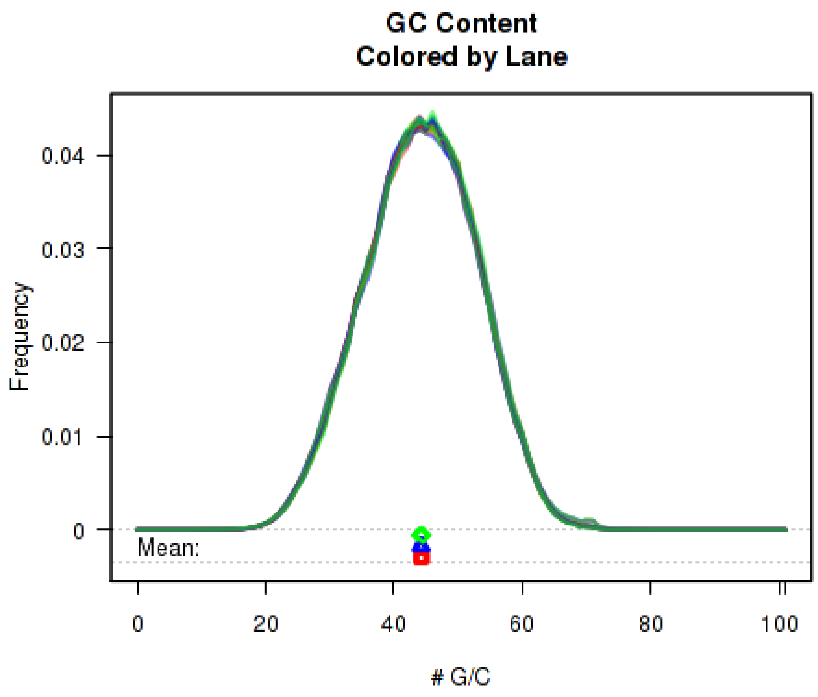

Figure 9: GC Bias

For each replicate, Figure 9 displays a histogram showing the frequency that different proportions of G and C (versus A, T, and N) appear in the replicate's reads. Each plotted line corresponds to a replicate. At the bottom of the plot the mean average G/C content is also plotted. Once again, the means are offset from one another by lane, to allow for easy detection of batch effects.

This plot can be generated individually with the command:

```
makePlot.gc(byLane.plotter);
```

The `byPair` option can be used to calculate the GC-distribution for read-pairs rather than for all reads individually. This is disabled by default because it often results in a jagged distribution when a appreciable proportion of the reads have an insert size equal to or smaller than the read length. When this occurs, the read-pair will almost always have an even number of G/C nucleotides.

> *What it means and what to look for:* GC bias has been indicated as a potential driver of false discoveries. Under certain circumstances the GC bias may vary by batch or by sample. If this is apparent in your dataset, particularly if it is associated with study conditions, one may need to apply a GC bias correction method (such as CQN).

7.4.3 Clipping Profile

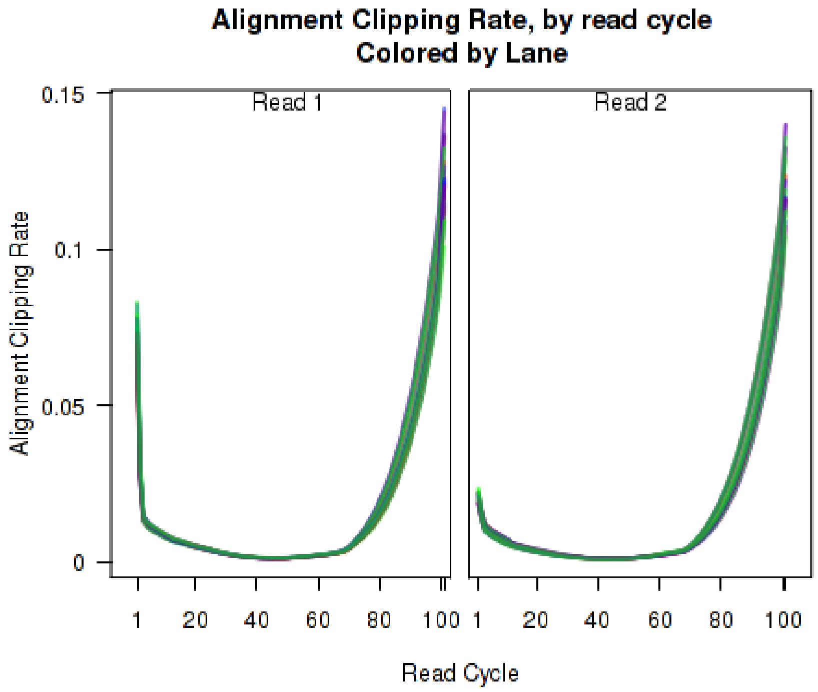

Figure 10: Clipping Profile

For each replicate, Figure 10 displays the rate (y-axis) at which the aligner soft-clips the reads as a function of read position (x-axis). Note that this will only be informative when using aligners that are capable of soft-clipped alignment (such as RNA-Star or GSNAP, but *not* TopHat).

This plot can be generated individually with the command:

```
makePlot.clipping(byLane.plotter);
```

What it means and what to look for: Abnormalities ("spikes", "shelves", or cases where sample groups are visibly different) in these plots can be caused by adaptor sequencing, gene fusions, mutations, population stratification, or differences in insert size.

7.4.4 Cigar Op Profile

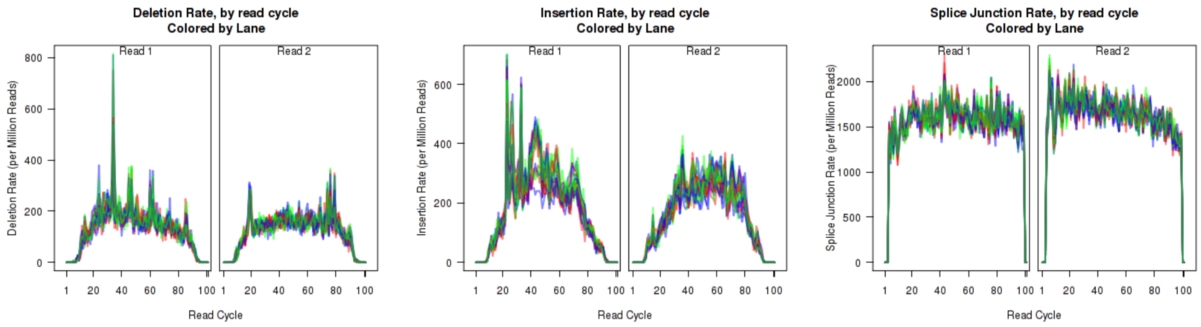

Figure 11: Cigar Operation Profiles

For each replicate, Figure 11 displays the rate (y-axis) of various cigar operations as a function of read position (x-axis). All 9 legal cigar operations can be plotted, but for most purposes only Deletions, Insertions, and Splice junctions will be informative.

This plot can be generated with the command:

```
makePlot.cigarOp.byCycle(byLane.plotter, "Del");
makePlot.cigarOp.byCycle(byLane.plotter, "Ins");
makePlot.cigarOp.byCycle(byLane.plotter, "Splice");
```

What it means and what to look for: These plots are most often used in conjuction with the plots in Section 7.4.5. Among other things these plots can reveal sequencer errors: a sequencer cycle-skip might result in a spike in the deletion rate at a particular cycle, whereas an incomplete wash may result in a spike in the insertion rate. These plots may also reveal biological differences like population stratification (eg: a sub-population disproportionately mismatches the reference genome), or broad genome rearrangements/editing (eg: in cancer cells).

In the example dataset, apparent spikes are plainly visible, stemming from deletions in one short and highly-expressed mitochondrial gene. The plots are also fairly noisy due to the small number of reads used in the example dataset and the extremely low frequency of deletions/insertions.

7.4.5 Cigar Length Distribution

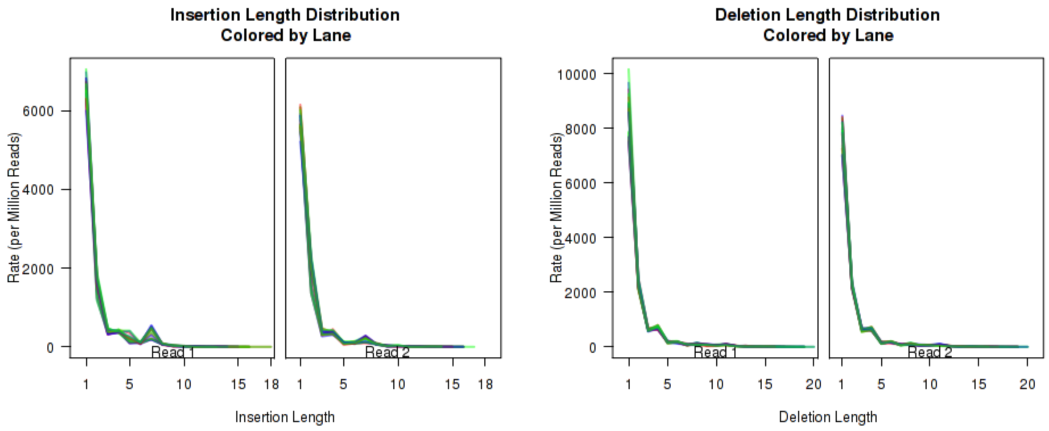

Figure 12: Cigar Length Distribution

The plots in Figure 12 display histograms of cigar operation lengths for each replicate.

These plots can be generated individually with the commands:

```
makePlot.cigarLength.distribution(byLane.plotter, "Ins");
makePlot.cigarLength.distribution(byLane.plotter, "Del")
```

What it means and what to look for: These plots are most often used in conjuction with the plots in Section 7.4.4. They can elucidate the nature of any oddities observed in the previous plots. For example: a large spike at one particular length may suggest that an apparent spike may be due simply to an unannotated variant in one particular high-expression gene, although further investigation is likely merited, to confirm that this is the case.

7.4.6 Insert Size

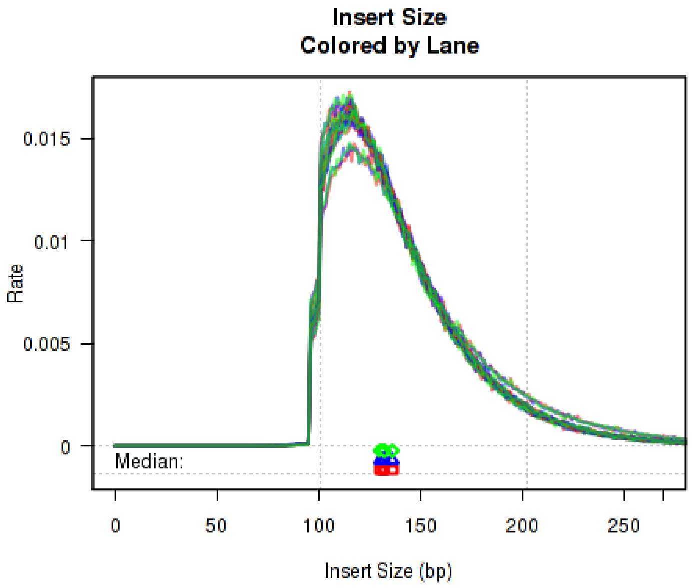

Figure 13: Insert Size

For each replicate, Figure 13 displays a histogram of the "insert size". Each line corresponds to one replicate, and displays the rate (y-axis) at which that replicate's reads possess a given insert size (x-axis).

Definition: "Insert Size": The "insert size" is the length (in base-pairs) between the two sequencing adapters for a pair of paired-end reads. In other words, it is the size of the original RNA fragment.

Insert Size Estimation: The Insert size is calculated using the alignment of the paired reads. When the two paired reads are aligned such that they overlap with one another the insert size can be calculated exactly. In such cases, the calculation of the insert size does *not* depend on the transcript annotation. However, when there is no overlap the exact insert size can be uncertain. Multiple splice junctions may lie in the region between the endpoints of the two paired reads, and there is no real way to determine which junctions the fragment used, if any. QoRTs uses the set of all splice junctions found between the endpoints of the two reads, and uses the shortest possible path from endpoint to endpoint. In some cases this may *under-estimate* the insert size, as the actual path may not be the shortest possible path. In other cases this may also *over-estimate* the insert size, if the RNA fragment includes novel splice junctions not found in the transcript annotation. However, in most cases this method appears to produce a reasonably good approximation of the insert size.

Note that the median average insert sizes for each replicate are plotted below the main plot. Each point corresponds to one replicate.

This plot can be generated individually with the command:

```
makePlot.insert.size(byLane.plotter);
```

Note: If the dataset is single-ended, this will generate a placeholder plot.

What it means and what to look for: Spikes in the insert size are common, generally the result of short, highly-expressed (often mitochondrial) transcripts. Because the size-selection of many RNA-Seq protocols is somewhat random, it is important to ensure that the resultant size selection is (relatively) consistent, and that variations are not associated with study condition status. If one study group has disproportionately high or low insert size, this could cause fragment bias that could drive the false discovery of differential effects.

7.4.7 N-Rate

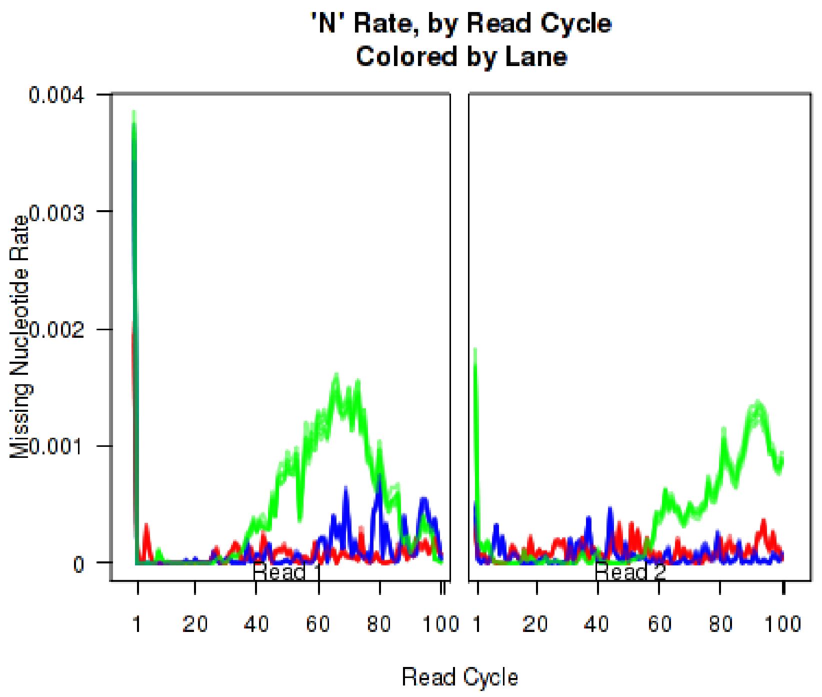

Figure 14: N-Rate plot

Figure 14 displays the rate (y-axis) at which the read sequence is "N" (or "missing"), as a function of the read position (x-axis). Each line corresponds to one replicate.

This plot can be generated individually with the command:

```
makePlot.missingness.rate(byLane.plotter);
```

What it means and what to look for: A number of potential sequencer issues can cause an abrupt "spike" or "shelf" in this plot. In one real sample assessed by QoRTs by the software author, it was determined that the sequencer camera was slightly offset on one specific cycle of one specific run. All the reads at the bottom or right edges were lost from that cycle forward, causing the rate of "N" calls to increase more than a hundred-fold. Once the problem was recognized the affected reads were identified and removed.

7.4.8 Gene-Body Coverage

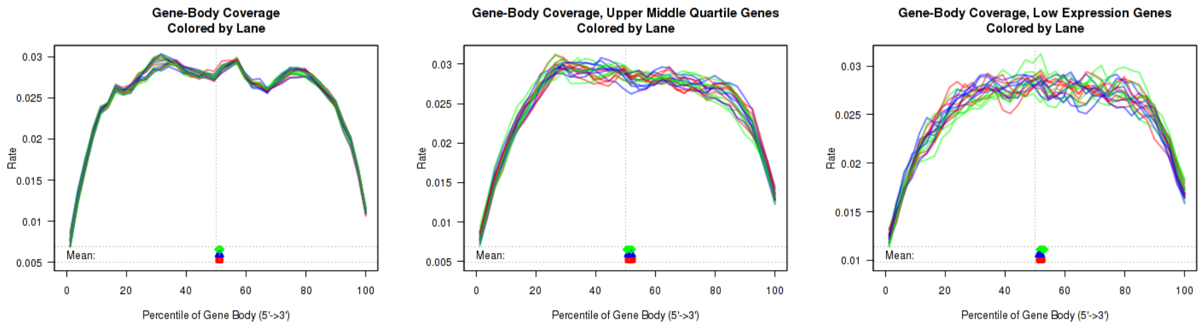

Figure 15: Gene-Body Coverage

For each replicate, the leftmost plot of Figure 15 displays the coverage profile across quantiles of all genes' lengths, from 5' to 3'. The middle plot displays the coverage profile for only the genes that are in the upper-middle quartile by read-count. The leftmost plot displays the coverage profile for the genes that are in the two lower quartiles.

Minor notes: To calculate the coverage profile, all the transcripts for each gene are merged together into a single "flat" pseudo-transcript which contains all exonic regions belonging to the gene. For each gene, the pseudo-transcript is broken up into 40 equal-length counting bins, so that each bin contains 2.5% of the total gene length. Each read-pair is counted once for every counting bin with which it overlaps. Genes are excluded from this analysis if they overlap with other genes or if they have zero reads for a given replicate. Additionally, any reads that overlap with more than one gene are automatically excluded.

This plot can be generated individually with the command:

```
makePlot.genebody.coverage(byLane.plotter);
makePlot.genebody.coverage.UMQuartile(byLane.plotter);
makePlot.genebody.coverage.lowExpress(byLane.plotter);
```

What it means and what to look for: When run on degraded RNA and/or when using poly-A selection, RNA-Seq often tends to have "3' bias", in which read coverage is higher on the 3' end of transcripts. The degree of this 3' bias tends to be dependent on the degree of degradation. Many analysis tools are robust against this issue when it occurs uniformly across the dataset; However, if some samples are substantially more degraded than others then this may cause problems downstream, particularly if RNA degradation is associated to the experimental condition(s). When studying this plot, check to make sure the gene-body coverage is consistant and/or matches your expectations.

Note that the overall gene-body coverage may be strongly influenced by extreme high-coverage genes (and potentially sequence-specific biases on those specific transcripts). Therefore the upper-middle-quartile plot is generally the preferred general metric for assessing overall gene-body coverage.

7.4.9 Cumulative Gene Diversity

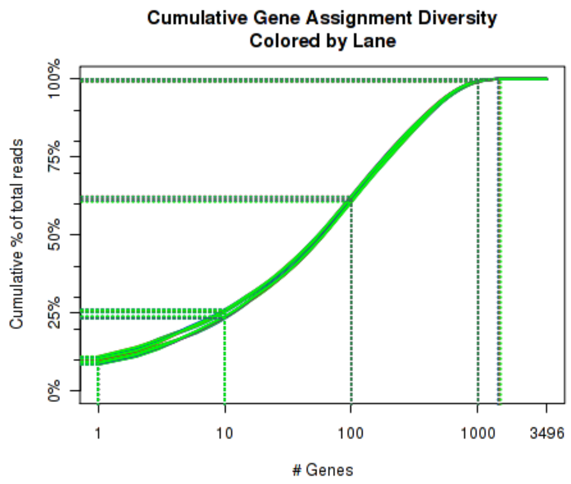

Figure 16: Cumulative Gene Diversity

For each replicate, Figure 16 displays the cumulative gene diversity. For each replicate, the genes are sorted by read-count. Then, a cumulative function is calculated for the percent of the total proportion of reads as a function of the number of genes. Intercepts are plotted as well, showing the cumulative percent for 1 gene, 10 genes, 100 genes, 1000 genes, and 10000 genes.

So, for example, across all the replicate, around 50 to 55 percent of the read-pairs were found to map to the top 1000 genes. Around 20 percent of the reads were found in the top 100 genes. And so on. This can be used as an indicator of whether a large proportion of the reads stem from of a small number of genes. Note that this is restricted to only the reads that map to a single unique gene. Reads that map to more than one gene, or that map to intronic or intergenic areas are ignored.

This plot can be generated individually with the command:

```
makePlot.gene.cdf(byLane.plotter);
```

What it means and what to look for: This plot can reveal a number of phenomena. First of all: if the top few genes dominate a sample (representing a large percentage of the total reads), oddities may appear in many of the other plots produced by QoRTs, as traits specific to these particular genes are dominant over the variation found across the genome.

It can also reveal biological variations: Different cell types or cells that are healthy, dying, or under stress often have very different diversity profiles from one another. If one sample of within a group is an extreme outlier it may suggest that something is wrong with that sample.

Or technical issues: These plots will often reveal inefficiency of hemoglobin or ribosome depletion protocols, and can also clearly reveal low library complexity (indicated by a very small number of genes being represented).

7.4.10 Nucleotide Rates, by Cycle

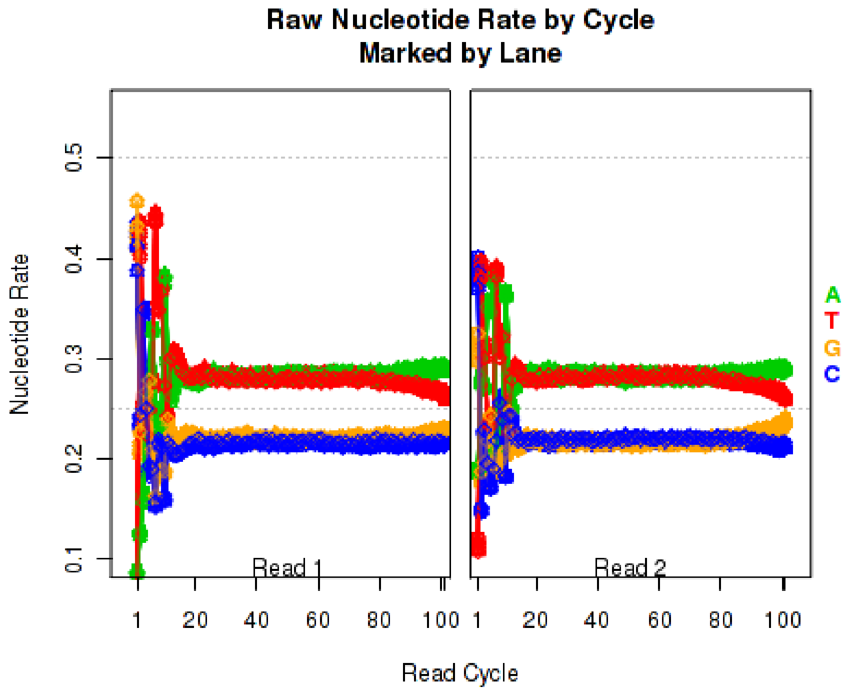

Figure 17: Nucleotide rates, by cycle

For each replicate, Figure 17 displays the rate at which each nucleotide appears (y-axis), as a function of the position in the read (x-axis). The color scheme for NVC plots is different from the other plots. Rather than being used for emphasis or to allow cross-comparisons by sample, biological-condition, or lane, the colors are used to indicate the four nucleotides: A (green), T (red), G (orange), or C (blue). Depending on the type of plotter being used, sample-runs will be marked and differentiated by marking the lines with shapes (R points). In many cases the points will be unreadable due to overplotting, but clear outliers that stray from the general trends can be readily identified.

When used with a "sample.highlight" type plotter (see 7.3.5), "highlighted" samples will be drawn with a deeper shade of the given color.

This plot displays the "raw" nucleotide rates, including bases that are soft-clipped by the aligner.

This plot can be generated individually with the command:

```
makePlot.raw.NVC(byLane.plotter);
```

What it means and what to look for: This can reveal sequence-specific biases such as hexamer or primer bias. Additionally, it can reveal adaptor sequencing. Such issues are generally not a problem as long as they are consistent across samples and groups.

7.4.11 Aligned Nucleotide Rates, by Cycle

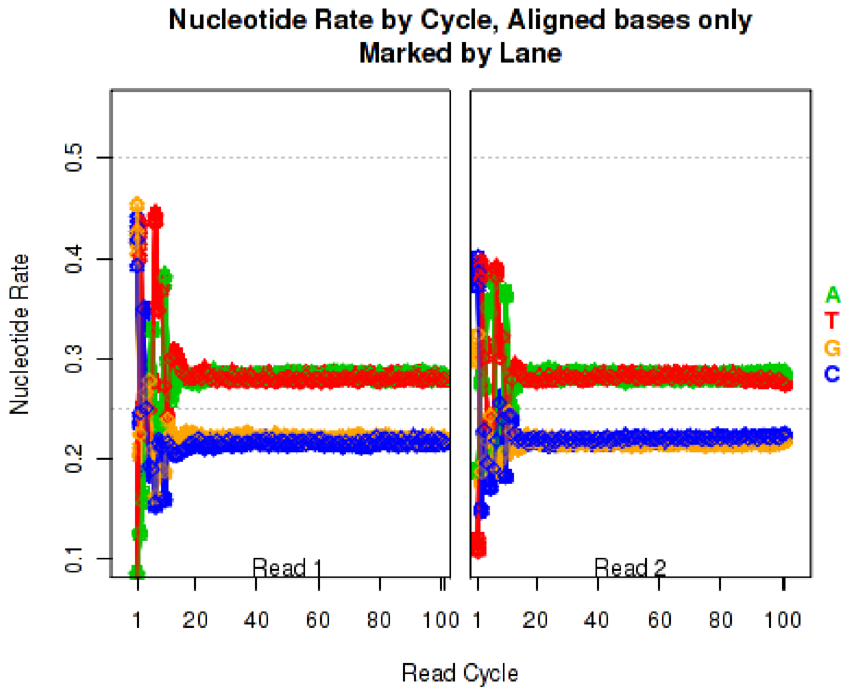

Figure 18: Aligned nucleotide rates, by cycle

Figure 18 is identical to Figure 17 (described in section 7.4.10), except that it only counts bases that are *not* soft clipped off by the aligner.

This plot can be generated individually with the command:

```
makePlot.minus.clipping.NVC(byLane.plotter);
```

What it means and what to look for: This can reveal sequence-specific biases such as hexamer or primer bias. Such issues are generally not a problem as long as they are consistent across samples and groups. Unlike the raw NVC plot, adaptor sequence will generally be absent from this plot as it usually will not align to the reference.

7.4.12 Leading Clipped Nucleotide Rates

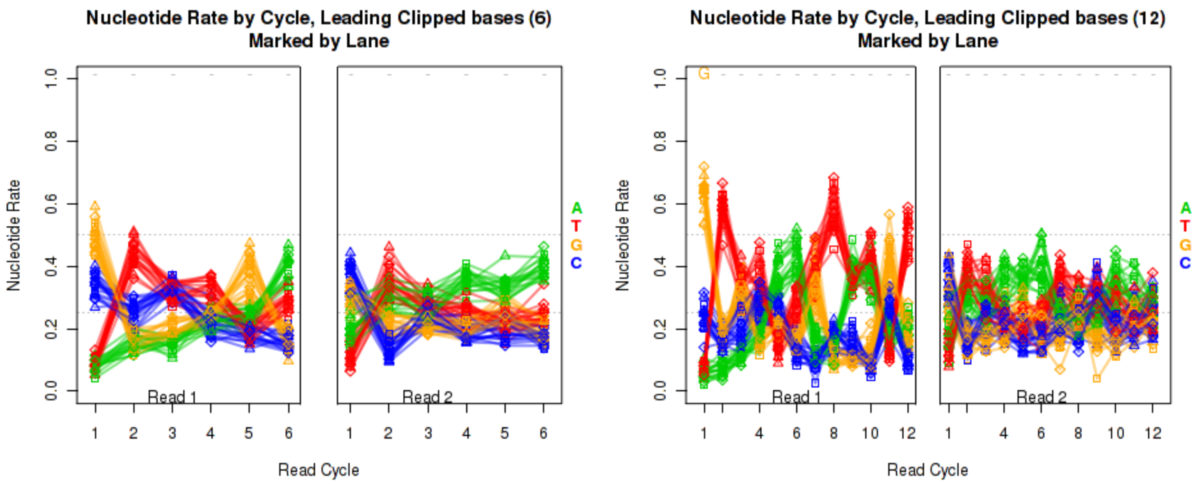

Figure 19: Leading-clipped nucleotide rates

The left plot in Figure 19 displays the nucleotide rate (y-axis) as a function of read position (x-axis), for the first 6 bases of reads in which *exactly* 6 bases were clipped off the 5' end. The right plot displays the nucleotide rate (y-axis) as a function of read position (x-axis), for the first 12 bases of reads in which *exactly* 12 bases were clipped off the 5' end.

This plot can be generated individually with the command:

```
makePlot.NVC.lead.clip(byLane.plotter, clip.amt = 6);
makePlot.NVC.lead.clip(byLane.plotter, clip.amt = 12);
```

Any integer can be used as the `clip.amt` value.

What it means and what to look for: If a large proportion of the reads are shorter than the read length then this can reveal the adaptor sequence.

7.4.13 Trailing Clipped Nucleotide Rates

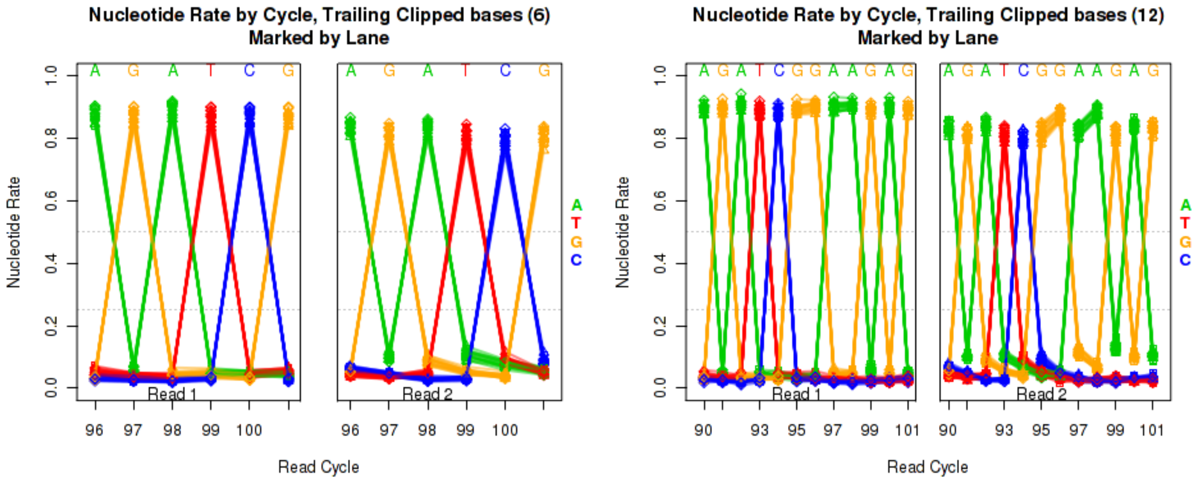

Figure 20: Trailing-clipped nucleotide rates

The left plot in Figure 20 displays the nucleotide rate (y-axis) as a function of read position (x-axis), for the last 6 bases of reads in which *exactly* 6 bases were clipped off the 3' end. The right plot displays the nucleotide rate (y-axis) as a function of read position (x-axis), for the last 12 bases of reads in which *exactly* 12 bases were clipped off the 3' end.

Note concerning the example data: In the example dataset an extremely strong trend is easily visible. The specific sequence observed matches that of the sequencing adapter used. The pattern appears in reads coming from fragments that are smaller than the read length. In these cases, the 3' end of each read will continue into the adapter sequence after sequencing the entire template fragment. Thus: for the left and right plots the sequence comes from reads with an insert size of exactly 95 and 89, respectively (ie 101 base pairs minus 6 or 12).

These plots can be generated individually with the command:

```
makePlot.NVC.tail.clip(byLane.plotter, clip.amt = 6);
makePlot.NVC.tail.clip(byLane.plotter, clip.amt = 12);
```

Any integer can be used as the `clip.amt` value.

What it means and what to look for: If a large proportion of the reads are shorter than the read length then this can reveal the adaptor sequence.

7.4.14 Mapping location rates

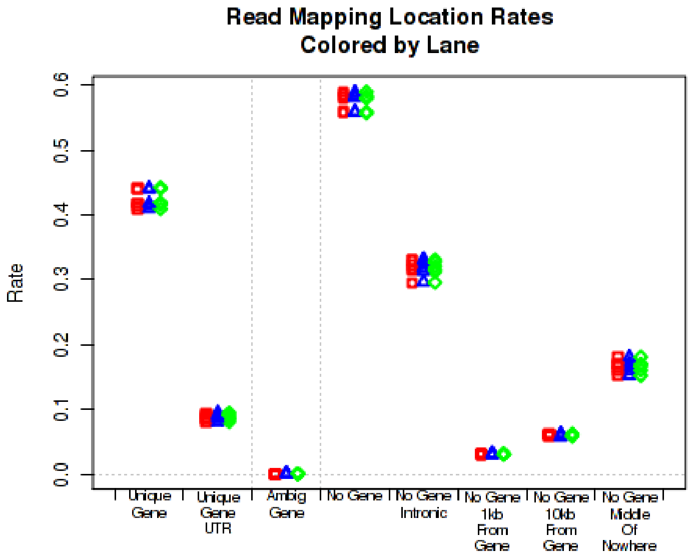

Figure 21: Gene assignment rates

For each replicate, Figure 21 displays the rate (y-axis) for which the replicate's read-pairs are assigned to the given categories.

The categories are:

- *Unique Gene*: The read-pair overlaps with the exonic segments of one and only one gene. For many downstream analyses tools, such as DESeq, DESeq2 [1] and *EdgeR* [2], only read-pairs in this category are used.
- *Ambig Gene*: The read-pair overlaps with the exons of more than one gene.
- *No Gene*: The read-pair does not overlap with the exons of any annotated gene.
- *No Gene, Intronic*: The read-pair does not overlap with the exons of any annotated gene, but appears in a region that is bridged by an annotated splice junction.
- *No Gene, 1kb from gene*: The read-pair does not overlap with the exons of any annotated gene, but is within 1 kilobase from the nearest annotated gene.
- *No Gene, 10kb from gene*: The read-pair does not overlap with the exons of any annotated gene, but is within 10 kilobases from the nearest annotated gene.
- *No Gene, middle of nowhere*: The read-pair does not overlap with the exons of any annotated gene, and is *more* than 10 kilobases from the nearest annotated gene.

This plot can be generated individually with the command:

```
makePlot.gene.assignment.rates(byLane.plotter);
```

What it means and what to look for: Outliers in these plots can indicate biological variations or the presence of large mapping problems. They may also suggest the presence of large, highly-expressed, unannotated transcripts or genes.

7.4.15 Splice Junction Loci

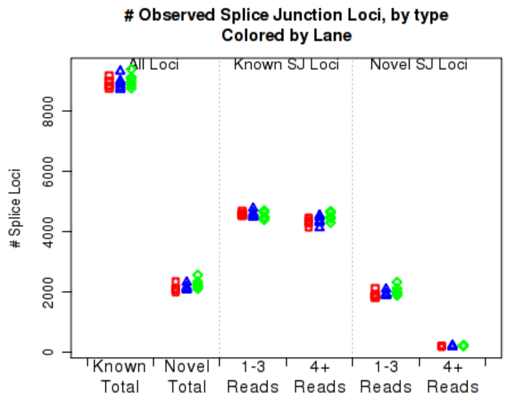

Figure 22: Splice junction loci

For each replicate, Figure 22 displays the number (y-axis) of splice junction *loci* of each type that appear in the replicate's reads. Splice junctions are split into 4 groups, first by whether the splice junction appears in the transcript annotation gtf ("known" vs "novel"), and then by whether the splice junction has 4 or more reads covering it or 1-3 reads.

The six categories of splice junction locus are:

- Known: The splice junction locus is found in the supplied transcript annotation gtf file.
- Novel: The splice junction locus is NOT found in the supplied transcript annotation gtf file.
- Known, 1-3 reads: The locus is known, and is only covered by 1-3 read-pairs.
- Known, 4+ reads: The locus is known, and is covered by 4 or more read-pairs.
- Novel, 1-3 reads: The locus is novel, and is only covered by 1-3 read-pairs.
- Novel, 4+ reads: The locus is novel, and is covered by 4 or more read-pairs.

This plot can be generated individually with the command:

```
makePlot.splice.junction.loci.counts(byLane.plotter);
```

What it means and what to look for: This plot can be used to detect a number of anomalies. For example: whether mapping or sequencing artifacts caused a disproportionate discovery of novel splice junctions in one sample or batch. It can also be used as an indicator of the comprehensiveness the genome annotation. Replicates that are obvious outliers may have sequencing/technical issues causing false detection of splice junctions.

Abnormalities in the splice junction rates are generally a symptom of larger issues which will generally be picked up by other metrics. Numerous factors can reduce the efficacy by which aligners map across splice junctions, and as such these plots become very important if the intended downstream analyses include transcript assembly, transcript deconvolution, differential splicing, or any other form of analysis that in some way involves the splice junctions themselves. These plots can be used to assess whether other minor abnormalities observed in the other plots are of sufficient severity to impact splice junction mapping and thus potentially compromise such analyses.

7.4.16 Number of Splice Junction Events

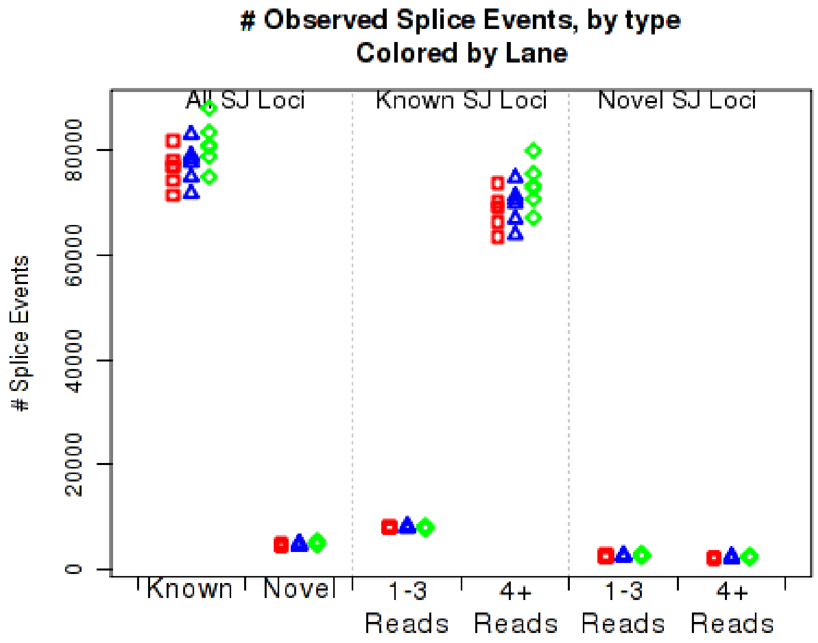

Figure 23: Number of splice junction events

For each replicate, Figure 23 displays the number (y-axis) of all splice junction events falling into each of the six junction categories. A splice junction "event" is one instance of a read-pair bridging a splice junction. Some reads may contain multiple splice junction events, some may contain none. If a splice junction appears on both reads of a read-pair, this is still only counted as a single "event".

Note that because different samples/runs may have different total read counts and/or library sizes, this function is generally not the best for comparing between samples. In general, the event rates per read-pair should be used, see the next section, 7.4.17.

This plot is used to detect whether sample-specific or batch effects have a substantial or biased effect on splice junction appearance, either due to differences in the original RNA, or due to artifacts that alter the rate at which the aligner maps across splice junctions.

This plot can be generated individually with the command:

```
makePlot.splice.junction.event.counts(byLane.plotter);
```

What it means and what to look for: This plot is useful for identifying mapping and/or annotation issues, and can indicate the comprehensiveness the genome annotation. Replicates that are obvious outliers may have sequencing/technical issues causing false detection of splice junctions.

In general, abnormalities in the splice junction rates are generally a symptom of larger issues which will often be picked up by other metrics. See Section 7.4.15.

7.4.17 Splice Junction Event Rates per Read-Pair

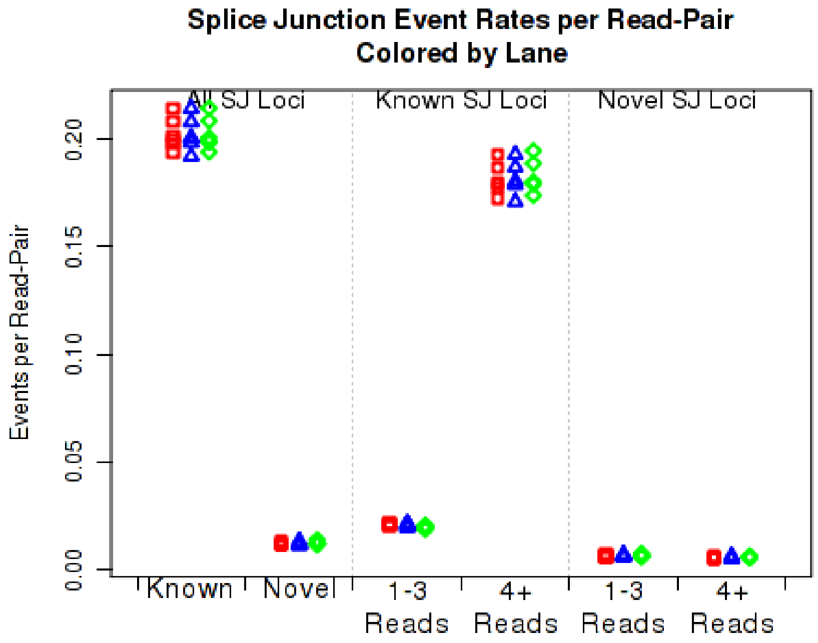

Figure 24: Splice junction events

For each replicate, Figure 24 displays the rate, per read-pair, (y-axis) at which each type of splice junction events appear. This is equivalent to the results seen in 7.4.16, except that each sample is scaled by the number of reads belonging to that sample.

This plot can be generated individually with the command:

```
makePlot.splice.junction.event.ratesPerRead(byLane.plotter);
```

What it means and what to look for: This plot is used to detect whether sample-specific or batch effects have a substantial or biased effect on splice junction appearance, either due to differences in the original RNA, or due to artifacts that alter the rate at which the aligner maps across splice junctions. It can assist in identifying mapping and/or annotation issues, and can indicate the comprehensiveness the genome annotation. Replicates that are obvious outliers may have sequencing/technical issues causing false detection of splice junctions.

In general, abnormalities in the splice junction rates are generally a symptom of larger issues which will often be picked up by other metrics. See Section 7.4.15.

7.4.18 Breakdown of Splice Junction Events

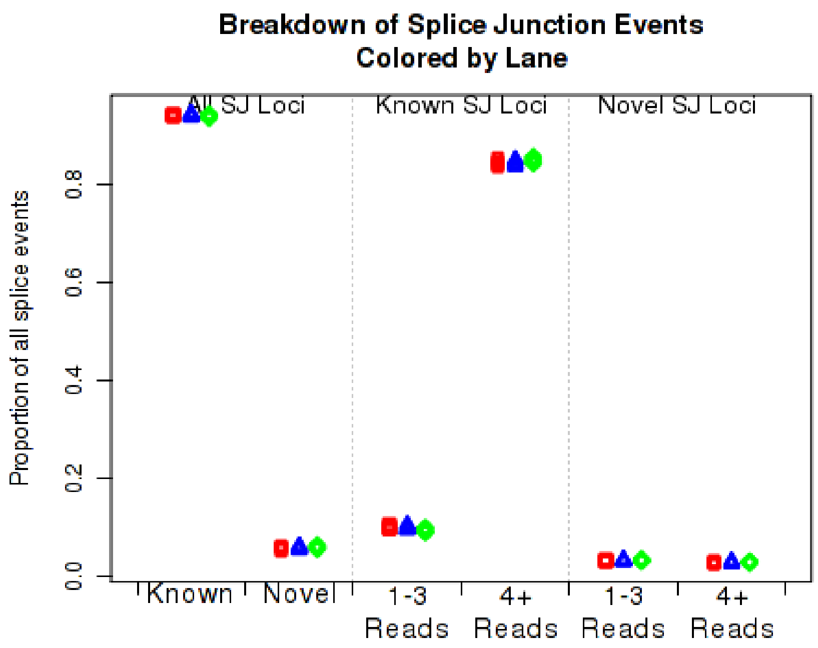

Figure 25: Proportions of splice junction events

For each replicate, Figure 25 displays the proportion of all splice junctions events that bridge splice junctions of each of the six splice junction types.

This plot is used to detect whether sample-specific or batch effects have a substantial or biased effect on splice junction appearance, either due to differences in the original RNA, or due to artifacts that alter the rate at which the aligner maps across splice junctions.

This plot can be generated individually with the command:

```
makePlot.splice.junction.event.proportions(byLane.plotter);
```

What it means and what to look for: This plot is used to detect whether sample-specific or batch effects have a substantial or biased effect on splice junction appearance, either due to differences in the original RNA, or due to artifacts that alter the rate at which the aligner maps across splice junctions. This plot is useful for identifying mapping and/or annotation issues, and can indicate the comprehensiveness the genome annotation. Replicates that are obvious outliers may have sequencing/technical issues causing false detection of splice junctions.

In general, abnormalities in the splice junction rates are generally a symptom of larger issues which will often be picked up by other metrics. See Section 7.4.15.

7.4.19 Breakdown of Splice Junction Events, by locus type

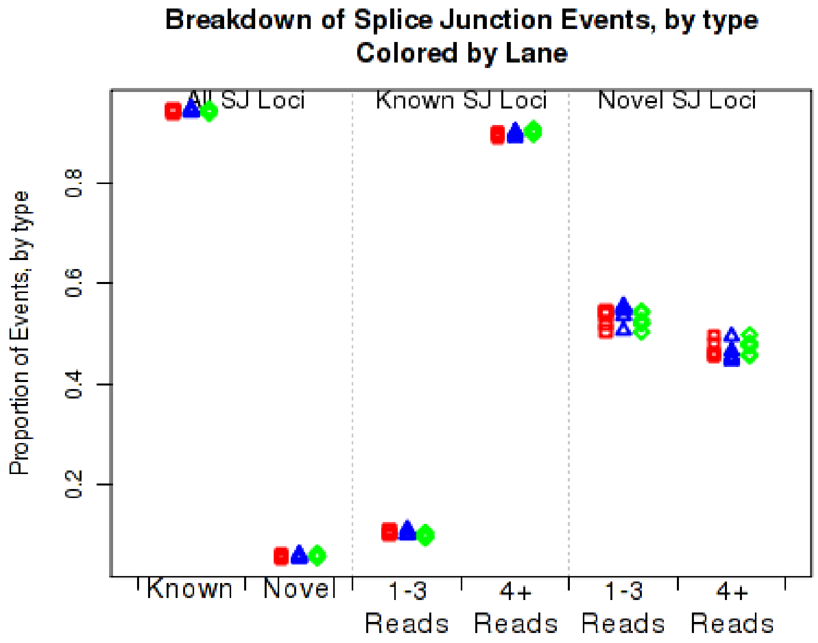

Figure 26: Splice junction events

In Figure 26 the left two columns display the proportion of splice junction events that are known vs novel. The middle columns display the proportion of known splice junction events that bridge junctions that have high (more than 4) vs low (1-3) read-pairs covering them. The right two columns display the proportion of novel splice junction events that bridge junctions that have high (more than 4) vs low (1-3) read-pairs covering them.

This plot can be generated individually with the command:

```
makePlot.splice.junction.event.proportionsByType(byLane.plotter);
```

What it means and what to look for: This plot is useful for identifying mapping and/or annotation issues, and can indicate the comprehensiveness the genome annotation. Replicates that are obvious outliers may have sequencing/technical issues causing false detection of splice junctions.

In general, abnormalities in the splice junction rates are generally a symptom of larger issues which will often be picked up by other metrics. See Section 7.4.15.

7.4.20 Strandedness test

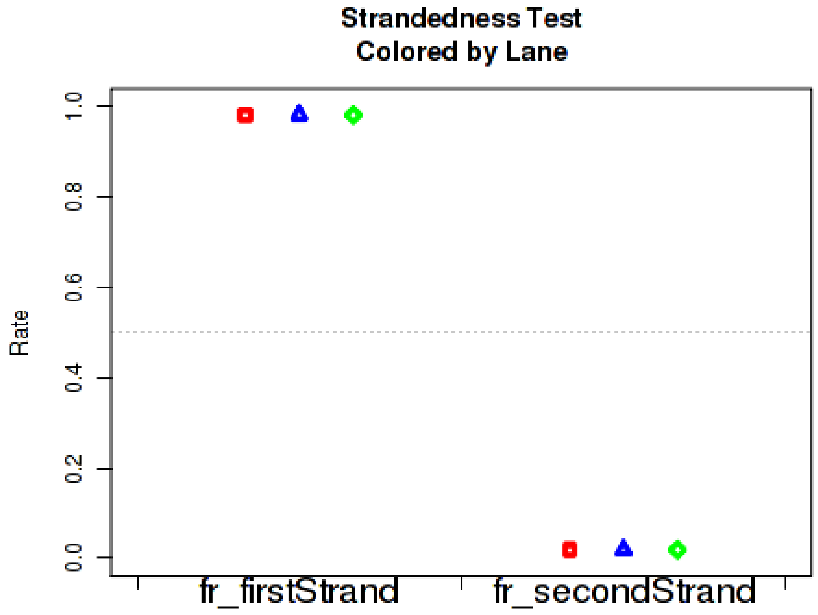

Figure 27: Strandedness

Figure 27 displays the rate at which reads appear to follow the two possible library-type strandedness rules. (See section 6 for more information on stranded library types).

This plot is used to detect whether your data is indeed stranded, and whether you are using the correct stranded data library type option. For unstranded libraries, one would expect all points to fall very close to the 50-50 center line. For stranded libraries, all points should fall closer to 99

This plot can be generated individually with the command:

```
makePlot.strandedness.test(byLane.plotter);
```

> *What it means and what to look for:* This plot can indicate the efficiency of the strand-specific selection protocol, and reveal variations in such efficiency. They can also be used to determine the "strandedness rule", which is required by many downstream analysis tools.

7.4.21 Mapping stats

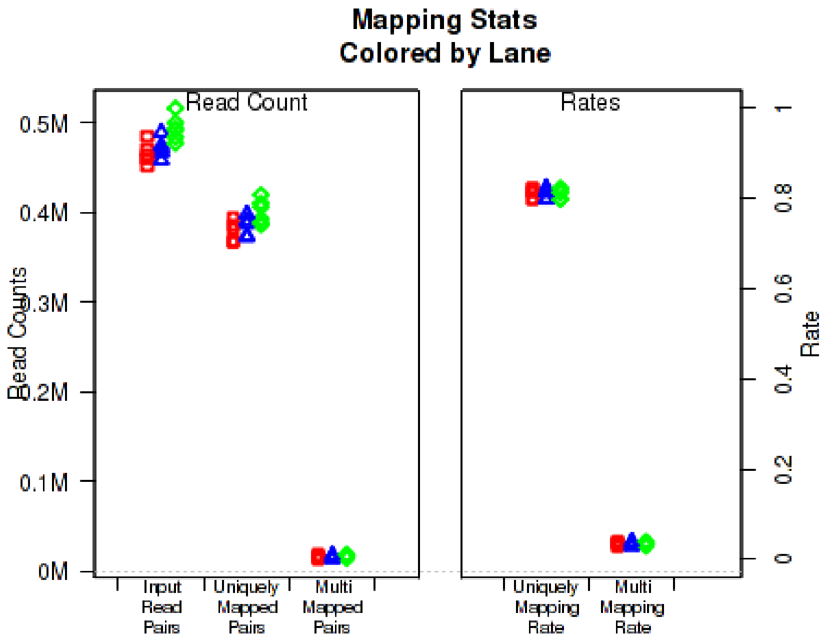

Figure 28: Mapping stats

For each replicate, Figure 28 displays the mapping rates and counts. In order to plot this data, QoRTs must be provided with the pre-alignment read-count for each replicate. There are a number of ways to provide this information to QoRTs. The easiest method is to list it specifically in the replicate decoder (see Section **??**). Alternatively, this information can be provided at the initial processing stage (see Section 6), either by setting the input read count explicitly using the `--seqReadCt` parameter, or by providing one of the unaligned fastq files via the `--rawfastq` parameter, in which case the input read count is calculated simply by dividing the number of lines in the fastq file by 4. For paired-end data, only one of the two fastq files needs to be provided, as both will have the same number of reads.

If the dataset contains multi-mapped reads, then numbers and rates of multi-mapping will be included in this plot. If multi-mapped reads were filtered out of the dataset prior to analysis with QoRTs, the multi-mapping rates can still be specified explicitly using the decoder (see Section **??**).

This plot can be generated individually with the command:

```
makePlot.mapping.rates(byLane.plotter);
```

If the input read count or multi-mapped count information is not found, this will generate a placeholder plot.

What it means and what to look for: Presence of outliers in the mapping rate statistics may be an indicator of large sample-prep, library-prep, or sequencer errors.

7.4.22 Chromosome counts

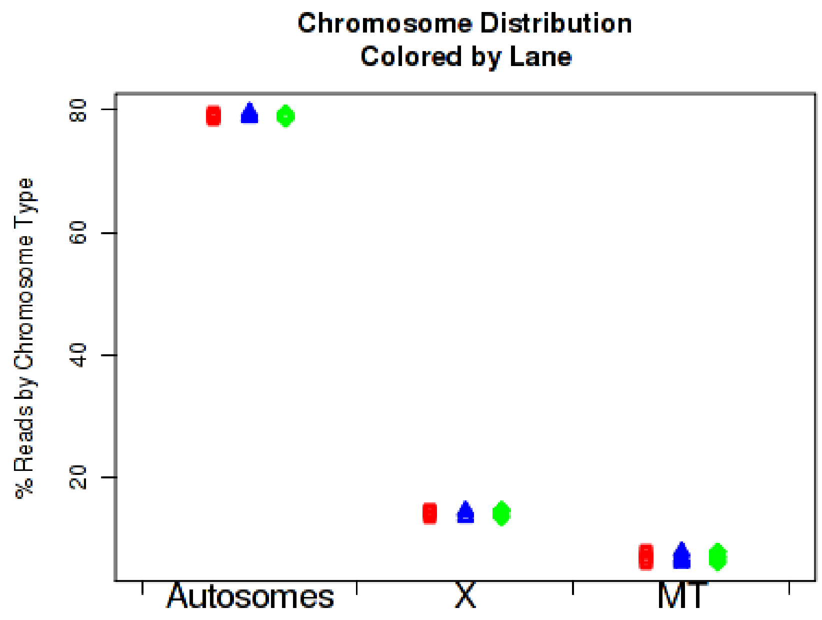

Figure 29: Mapping stats

For each replicate, Figure 29 displays the number of read-pairs mapping to each category of chromosome.

The `chromosome.name.style` must be set to match the style of your chromosome names. By default it assumes the chromosomes are named chr1, chr2, chr3, etc.

For more information, see the help document using the command `help(makePlot.chrom.type.rates)`.

This plot can be generated individually with the command:

```
makePlot.chrom.type.rates(byLane.plotter);
```

What it means and what to look for: Presence of outliers in these plots may point to variable inefficiency in a ribosomal/mitochondrial depletion protocol. In most datasets the Y chromosome counts can be used to determine sample sex (but not in this case, since the Y chromosome is not included in the example dataset's genome assembly). The raw metrics generated by QoRTs can also be used to generate counts for the ERCC spike-ins or similar.

7.4.23 Normalization Factors

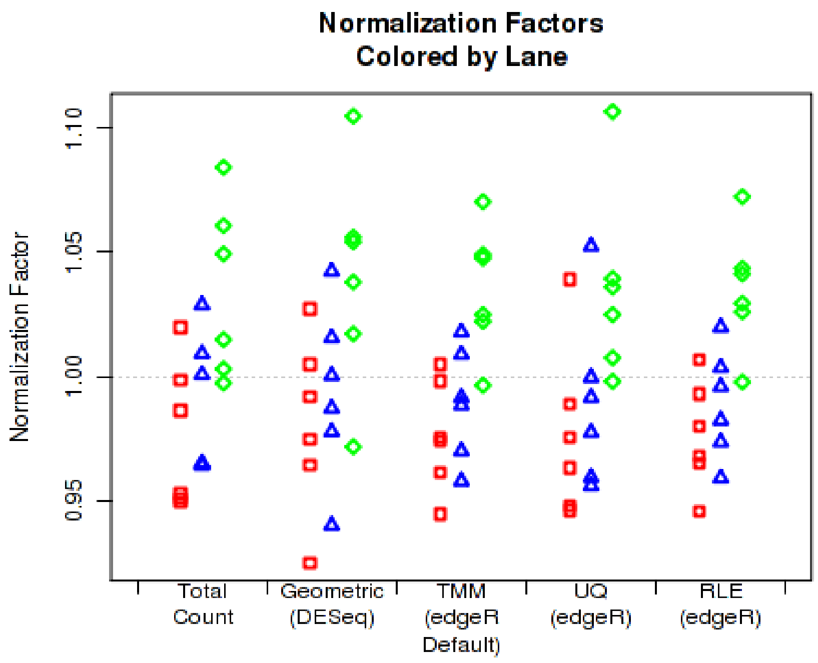

Figure 30: Normalization Factors

For each replicate, Figure 30 displays the normalization factors.

By default, QoRTs will automatically detect whether *DESeq2* and *edgeR* are installed and will use these tools to calculate their respective normalization size factors. If neither package is found, then it will only plot the total count normalization.

This plot can be generated individually with the command:

```
makePlot.norm.factors(byLane.plotter);
```

What it means and what to look for: These normalization factors can be used for a number of downstream analyses, including the generation of summary browser tracks.

7.4.24 Normalization Factor Ratio

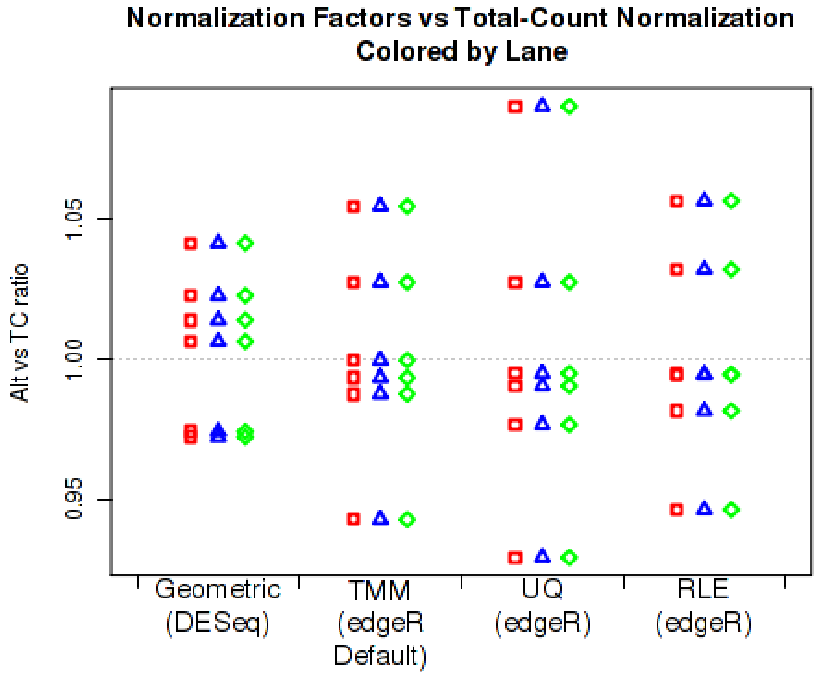

Figure 31: Normalization Factors vs TC

For each replicate, Figure 31 displays the ratio of the alternate normalization factors to the Total Count normalization factors.

By default, QoRTs will automatically detect whether *DESeq2* and *edgeR* are installed and will use these tools to calculate their respective normalization size factors. If neither package is found, then it will only plot the total count normalization.

This plot can be generated individually with the command:

```
makePlot.norm.factors.vs.TC(byLane.plotter);
```

What it means and what to look for: Large variations in these ratios can indicate large-scale differences between the samples.

7.4.25 Read drop rate

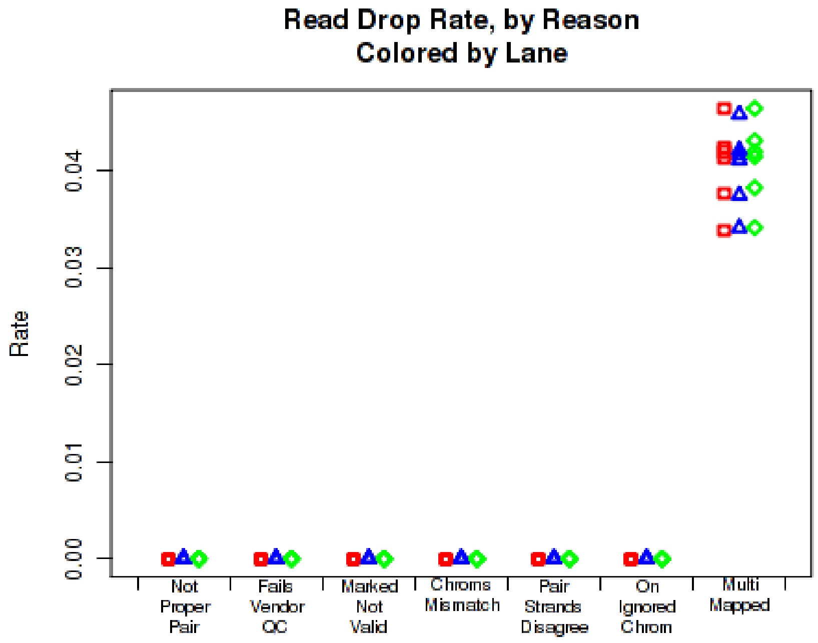

Figure 32: Drop rates

For each replicate, Figure 32 displays the rates and reasons for reads being dropped from QC analysis. Note that in the example dataset reads were never dropped. This is a consequence of the pre-processing steps in the example pipeline.

This plot can be generated individually with the command:

```
makePlot.dropped.rates(byLane.plotter);
```

What it means and what to look for: This can be used to assess the occurrance rates of a number of failure modes.

8 Identifying Problems

QoRTs produces a vast array of output data, and the interpretation of said data can be difficult.

Proper quality control must consider the study design, sequencer technology, study species, read length, library preparation protocol, and numerous other factors that might affect the metrics produced by QoRTs. In some datasets, apparent "abnormalities" may be expected. Similarly, depending on the type of downstream analysis that is being performed, some errors or artifacts may be irrelevant.

When an unexplained abnormality is recognized, one must decide what to do with the data. Unfortunetely this question is nontrivial, and depends on numerous factors. Bioinformaticians must be aware of the statistical assumptions that are being made in the downstream analyses, and must consider the conditions under which such assumptions would be violated.

Some abnormalities will not affect a given analysis and thus can be ignored outright. Some may require that the offending sample(s) be removed from the analysis. Others may necessitate additional steps to normalize the data or adjust for confounding factors. And finally, some artifacts may be so severe that the intended analysis will be impossible using the supplied data. Ultimately bioinformaticians must use their own judgement to determine what action should be taken, should an abnormality be discovered.

Here we present two examples of data-quality issues that were incidentally discovered during the development of the QoRTs software package. Note that the sequencing data presented in these examples are atypical, and were chosen because they illustrated succinctly the abnormalities in the data.

8.1 Example 1: Sequencer Hiccup

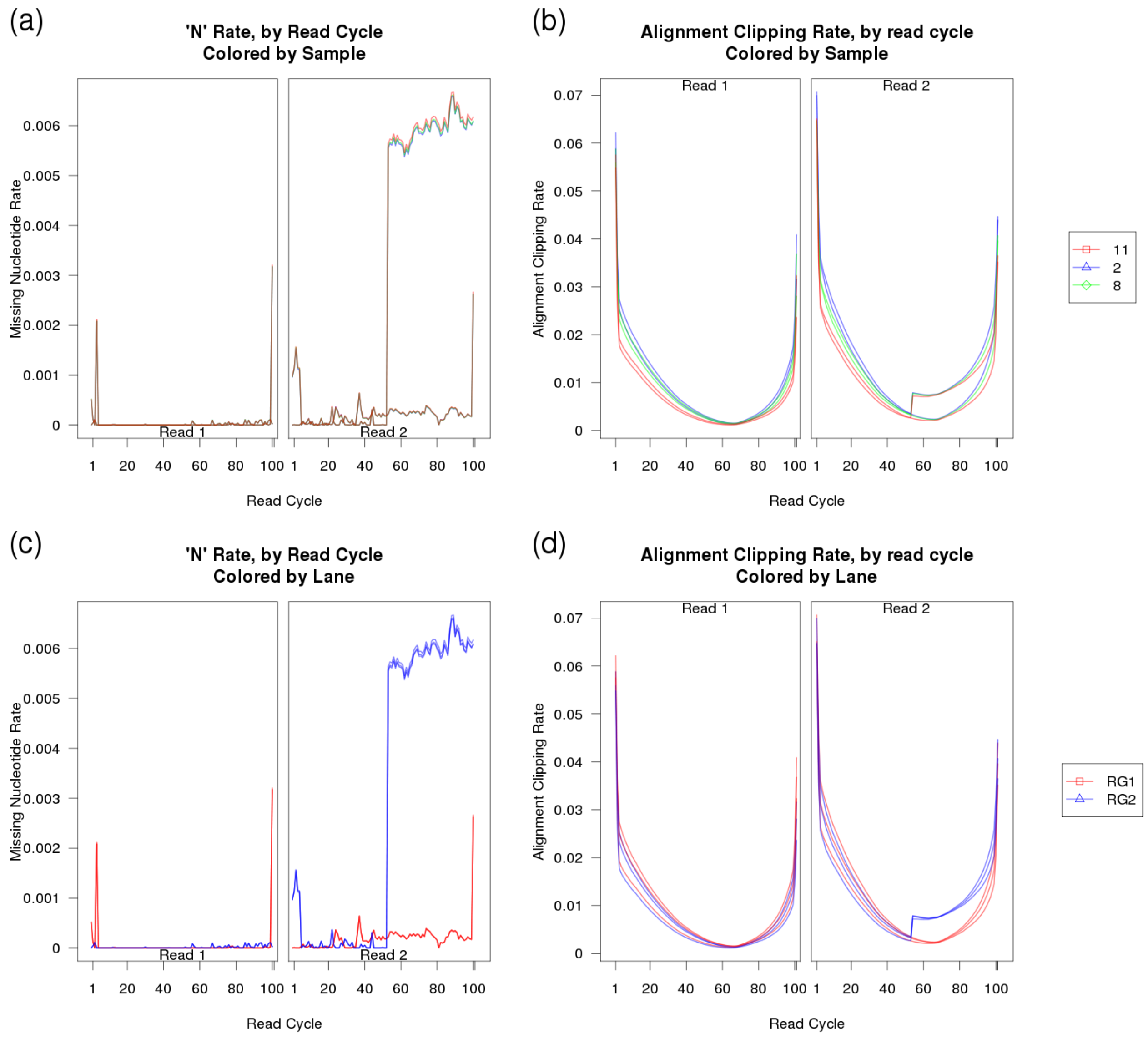

Figure 33: An example of a QC anomaly discovered incidentally during the development of QoRTs.

In our first example, an rare hardware-level fault of unknown origin caused a shift in the sequencing scanner at the 53rd read cycle of read 2. As a result, a small fraction of the reads (around half of a percent) were marked with N's for the remaining 48 bases, as they were now outside the limits of the image frame. This appears to have been a freak occurance: we have not seen this issue before or since. Selected plots from this QC run are displayed in Figure 33.

As you can see in Figure 33a and c, in some of the replicates the rate at which the sequencer assigned N's increased more than 10-fold after cycle 53. Furthermore, in figures 33b and d, the replicates also displayed a small but abrupt increase in the clipping rate. This was due to the fact that aligner (RNA-Star) always clipped reads when they had no remaining "called" nucleotides.

These plots not only point out the existance of a problem, when examined together they can also be used to substantially narrow down the list of possibile underlying causes. In 33a and b it can be clearly seen that the abnormality is not consistent within samples, ruling out errors in sample or library prep, or indeed anything prior to the sequencing itself. If the issue was due to an artifact occurring prior to sequencing, it would be consistent across technical replicates. Examining Figure 33c and d, it becomes obvious that the issue is specific to one of the sequencer lanes. The abnormality only appears in lane RG2, and not in lane RG1.

Finally, based on examination of 33b and d it can be inferred that the excess missingness is most likely NOT uniformly or randomly distributed across all reads in the affected replicates. If the excess N's were evenly distributed then you would not expect a sudden and dramatic increase in the clipping rate, since RNA-STAR would still be able to align the remaining called bases in each read. Instead, we can hypothesize that this effect is specific to a small subset of reads which have "tails" of repeated N's starting at cycle 53.

This breadth of information considerably narrows the possible set of underlying causes.

When "N" rate was examined as a function of lane coordinate (via a custom-built script), we were able to identify problem and correct it. Since the reads affected were solely a function of the location on the physical flowcell, and since only a small percentage of reads were affected at all, we elected to simply drop the truncated reads.

8.2 Example 2: Badly Degraded RNA

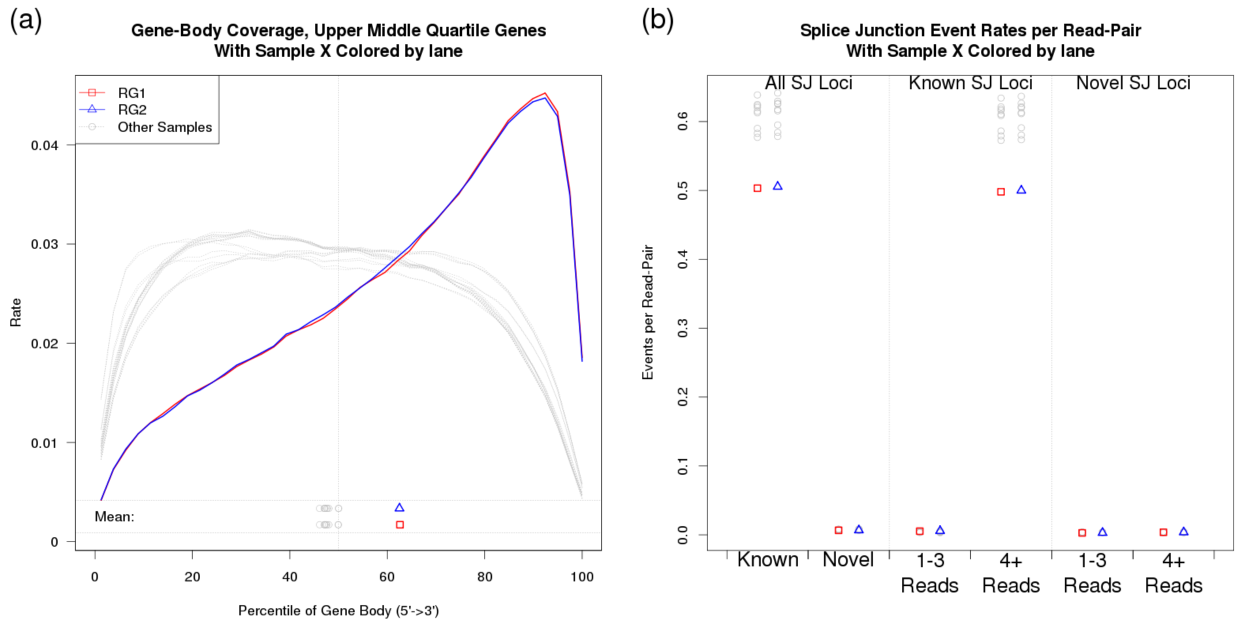

Figure 34: Another example of a QC anomaly discovered incidentally during the development of QoRTs.

In our second case study, one particular RNA sample was substantially more degraded than the others. All samples were poly-A selected, so this heightened degradation resulted in a strong 3' bias.

In Figure 34 the affected sample is plotted in red and blue (indicating the two technical replicates), whereas all the other samples are plotted in grey. The vastly-increased 3' bias can be clearly identified in 34a. Figure 34b shows that this had a substantial effect on the rate at which splice junction events were observed. Excess 3' bias can have broad impact, compromising estimates of gene-level and transcript-level abundance, and consequently compromising differential expression or differential splicing analyses.

As a result, outliers like this should generally be dropped prior to analysis.

9 Secondary Utilities

In addition to the standard quality-control tools described in the previous sections, QoRTs also includes a number of other minor utilities intended to assist in data visualization, cleaning, and preparation for downstream analyses.

9.1 Generating a flattened annotation file

Before counting exons and splice junctions, QoRTs generates a set of non-overlapping exonic fragments out of all the exons in the genome annotation gtf file. It then assigns each exonic fragment a unique identifier. Similarly, it assigns every splice junction its own unique identifier. A gtf file listing all these genomic features and their unique identifiers can be created using the following command:

```
java -jar /path/to/jarfile/QoRTs.jar makeFlatGtf \
                                     input.gtf \
                                     flattened.gff
```

Both the input and output annotation files can be either .zip or .gz compressed. Compression is autodetected from the file extension.

strandedness: You must use the `--stranded` option to create the flattened gff for use with stranded datasets. DO NOT mix stranded flattened gff with unstranded data, or vice versa.

DEXSeq: DEXSeq also requires a flattened annotation file, which is formatted similarly. In order to produce a flattened gff file that DEXSeq can read, include the `--DEXSeqFmt` option.

This gtf file conforms to the UCSC gff file definition, (found here: http://genome.ucsc.edu/FAQ/FAQformat.html). It will contain 4 different feature types (column 3): "aggregate_gene", "exonic_part", "splice_site", and "novel_splice_site".

9.2 Merging Count Data

For the purposes of quality control it is generally preferable to run QoRTs on each sample-run individually, so that potential technical artifacts related to sequencing run or lane can be identified. However, for most downstream purposes these "technical replicates" will be combined and treated as a single sample. Differential expression tools like *DESeq*, *DESeq2* [1], *DEXSeq* [3], and *EdgeR* [7] assume that each set of gene counts (or exon counts, for *DEXSeq*) is derived from a different biological sample.

Thus, the java utility includes a function for quickly and easily calculating merged sample-wise counts:

```
java -jar /path/to/jarfile/QoRTs.jar mergeAllCounts \
                                    decoder.txt  \
                                    /path/to/qc/results/dir/  \
                                    ./merged/
```

This decoder MUST contain the unique.ID and sample.ID columns.

Alternatively, the merger can be performed for a single sample directly, via the command:

```
java -jar /path/to/jarfile/QoRTs.jar mergeCounts
          ./SAMP1_RG1/,./SAMP1_RG2/,./SAMP1_RG3/
          ./merged/SAMP1/
```

The list of QC data directories must be separated by commas and contain no whitespace.

More information and a full accounting of all parameters and options can be found in the online documentation[7], or by using the commands:

`java -jar /path/to/jarfile/QoRTs.jar mergeAllCounts--man`
and
`java -jar /path/to/jarfile/QoRTs.jar mergeCounts --man`

9.3 Generating genome browser tracks

In addition to the standard QC plots, which examine the data as a whole, it is sometimes desirable to be able to query and examine coverage information at specific genetic loci. In particular, when identifying candidate genes via genome-wide analyses, it is often vital to examine the locus for artifacts before carrying out costly and time-consuming validation experiments.

Figure 35 is just one example of the tracks that can be produced with QoRTs. A full description of how this particular splice junction track can be generated can be found in the example dataset walkthrough, which is linked to on the QoRTs github site.

9.3.1 Generating wiggle tracks

QoRTs includes a utility to generate ".wig", or "wiggle plot" files. These wiggle plot files include counts for the mean coverage for each equal-sized window across the whole genome. These files are designed to be used with the UCSC browser or similar interfaces, and allow easy and intuitive visualization of your data.

```
java -jar /path/to/jarfile/QoRTs.jar bamToWiggle
                                     infile.bam
                                     trackName
                                     chromLengthFile
                                     outfilePrefix
```

The chromLengthFile is a simple tab-delimited text file that includes each chromosome in the first column and the chromosome's length (in base-pairs) in the second column. If the wiggle file is intended for use with a standard genome on the UCSC genome browser, then the UCSC utility `fetchChromSizes` should be used to generate this file. (see http://genome.ucsc.edu/goldenPath/help/bigWig.html for more information on `fetchChromSizes`, as well as information on how to compress your wig files into smaller and more efficient bigWig files). It also takes

[7] Found here: http://hartleys.github.io/QoRTs/jarHtml/index.html

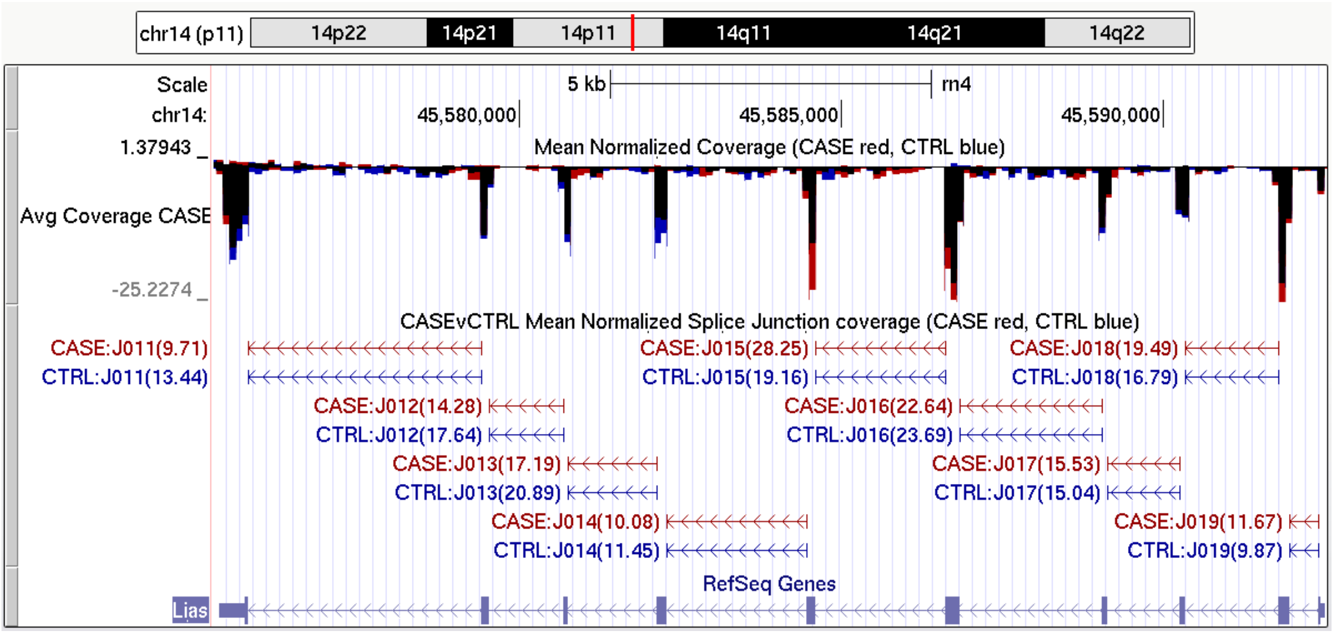

Figure 35: An example of some of the tracks that can be generated via QoRTs. The first track ("CTRL_FWD") shows the mean normalized coverage depth for 100-base-pair windows for the forward (genomic) strand. The second track reveals the coverage depth on the negative strand (plotted as negative values). The third track ("CASEvCTRL Junction Counts") displays the locus ID for each splice junction, along with the mean normalized coverage bridging the splice junction loci in cases and controls. Note that cases and controls are colored distinctly, for ease of use.

Common options and flags for this function include:

`--sizefactor 1.0` : A float value. All the coverage values will be divided by this factor. Useful for comparing two samples that may have different normalization factors.

`--stranded` : Flag to indicate that data should be treated as stranded.

`--fr_secondStrand` : Flag to indicate that the data is of the fr_secondstrand stranded library type. (See section 6 for more information on the two stranded library types).

`--negativeReverseStrand` : If this flag is set, then the negative strand will be counted in negative numbers. This can be useful for plotting both strands in a single multiwig track, via a trackhub. (see `http://genome.ucsc.edu/goldenPath/help/trackDb/trackDbDoc.html`)

`--simpleCountByRead` : If this flag is raised, then each read of each read-pair will be counted separately. Thus the wiggle plot will count simple read coverage depth rather than read-pair coverage depth. This means that when read-pairs overlap they will be counted twice over the overlapping region. This option will have no effect on single-ended data.

Many of the other parameters are identical to those used by the QC tool: `--singleEnded`, `--nameSorted`, etc.

More information and a full accounting of all parameters and options can be found in the online documentation[8], or by using the command:

[8] Found here: `http://hartleys.github.io/QoRTs/jarHtml/index.html`

```
java -jar /path/to/jarfile/QoRTs.jar bamToWiggle --man
```

9.3.2 Merging wiggle tracks

QoRTs includes a utility for summing or averaging multiple wiggle files, either with or without normalization factors. For example, to calculate the normalized mean coverage for each 100-bp window across all CASE samples in the example dataset:

```
java -jar /path/to/jarfile/QoRTs.jar mergeWig \
                --calcMean \
                --filenames outputData/countTables/SAMP1/QC.wiggle.fwd.wig.gz,output\
Data/countTables/SAMP2/QC.wiggle.fwd.wig.gz,outputData/countTables/SAMP3/QC.wiggle.f\
wd.wig.gz \
                --sizeFactors 1.057995,0.999932,1.015372 \
                path/to/output/CASE.fwd.wig.gz
```

There are a number of other alternative parameterizations. The `--sampleList` parameter (which can be either a comma-delimited list or a ".txt" file containing a list) can be used along with the `--infilePrefix` and `--infileSuffix` to specify the file names if all of the wiggle files are in the same parent directory. The size factors can also be provided in a tab-delimited file using the `--sizeFactorFile` parameter. If the `--sizeFactors` and `--sizeFactorFile` parameters are omitted then the non-normalized sums/means will be calculated.

Common options and flags for this function include:

`--calcMean` : If this flag is raised, the utility will calculate the average rather than the total coverage for each window.

`--makeNegative` : If this flag is raised, all output values will be multiplied by -1.

`--sizeFactorFile` : A file containing at least two columns, the sample.ID and the size.factor. This file must include all samples in the sample list, but can include other samples that are not included in the sample list.

More information and a full accounting of all parameters and options can be found in the online documentation[9], or by using the command:

```
java -jar /path/to/jarfile/QoRTs.jar mergeWig --man
```

9.3.3 Generating splice-junction tracks

To visualize splice junction data, QoRTs can produce bed files that show splice junction counts:

```
java -jar /path/to/jarfile/QoRTs.jar makeJunctionTrack  \
                --stranded \
                --filenames outputData/countTables/SAMP1/QC.spliceJunctionAndExonCou\
nts.withNovel.forJunctionSeq.txt.gz \
```

[9] Found here: http://hartleys.github.io/QoRTs/jarHtml/mergeWig.html

```
                flattened.gff \
                path/to/output/CASE.bed.gz
```

Common options and flags for this function include:

`--rgb r,g,b` : The color to use for each bed entry. Three integers, comma-delimited (with no spaces), each between 0 and 255.
`--stranded` : Whether the data is stranded.
`--sizeFactors sf1,sf2,...` : A list of size factors with which to normalize replicates to a common scale. The list must have the same length as the number of replicates provided (ie the filenames parameter).
`--skipNovelJunctions` : If this option is used, novel splice junctions will not be included in the output file.
`--title` : A prefix to append to each splice junction ID.

More information and a full accounting of all parameters and options can be found in the online documentation[10], or by using the command:

```
java -jar /path/to/jarfile/QoRTs.jar makeJunctionTrack --man
```

9.3.4 Merging splice-junction tracks

Merged splice junction tracks can be created using the same utility used to create single-sample splice junction tracks. This uses syntax similar to the syntax used for merging wiggle files:

```
java -jar /path/to/jarfile/QoRTs.jar makeJunctionTrack  \
                --calcMean \
                --stranded \
                --filenames  outputData/countTables/SAMP1/QC.spliceJunctionAndExonCo\
unts.withNovel.forJunctionSeq.txt.gz,outputData/countTables/SAMP2/QC.spliceJunctionA\
ndExonCounts.withNovel.forJunctionSeq.txt.gz,outputData/countTables/SAMP3/QC.spliceJ\
unctionAndExonCounts.withNovel.forJunctionSeq.txt.gz \
                --sizeFactors 1.057995,0.999932,1.015372 \
                flattened.gff \
                path/to/output/CASE.bed.gz
```

As with the wiggle-file merge utility, there are a number of other alternative parameterizations. The `--sampleList` parameter (which can be either a comma-delimited list or a ".txt" file containing a list) can be used along with the `--infilePrefix` and `--infileSuffix` to specify the file names if all of the wiggle files are in the same parent directory. The size factors can also be provided in a tab-delimited file using the `--sizeFactorFile` parameter. If the `--sizeFactors` and `--sizeFactorFile` parameters are omitted then the non-normalized sums/means will be calculated.

More information and a full accounting of all parameters and options can be found in the online documentation[11], or by using the command:

[10] Found here: http://hartleys.github.io/QoRTs/jarHtml/makeJunctionTrack.html
[11] Found here: http://hartleys.github.io/QoRTs/jarHtml/makeJunctionTrack.html

```
java -jar /path/to/jarfile/QoRTs.jar makeJunctionTrack --man
```

9.4 Importing data into other tools

In addition to providing quality control information, QoRTs also provides the requisite input files needed for the DESeq/DESeq2 [1], *DEXSeq* [3], and *EdgeR* [2,7,8] analysis tools. These files will be identical to those that would be generated by HTSeq (using the default "union rule" option).

All the data files can be found in the `qc.data.dir` directory. The files for use with *DESeq*, *DESeq2*, and *EdgeR* will be named `QC.geneCounts.formatted.for.DESeq.txt.gz` and the files for use with *DEXSeq* will be named `QC.exonCounts.formatted.for.DEXSeq.txt.gz`

9.4.1 DEXSeq compatibility

A note on the DEXSeq counts: The DEXSeq counts may not be perfectly identical to those produced by the `dexseq_prepare_annotation.py` and `dexseq_count.py` scripts. There are two reasons for these differences, both relating to the treatment of aggregate genes. The first reason is minor: Aggregate genes will be named slightly differently. When multiple genes overlap with one another, DEXSeq produces "aggregate genes" which include all transcripts for all these overlapping genes. It names the aggregate gene using the set of genes in the aggregate, delimited with + characters. Unfortunately, the genes are drawn from an unordered set and thus not listed in any defined order. Thus, it is not possible for QoRTs to replicate the exact same order in multi-gene aggregates. QoRTs lists the contained genes in lexicographic order.

Secondly, for UNSTRANDED data, the QoRTs and DEXSeq annotation "flattening" step will behave slightly differently under default conditions. DEXSeq's `dexseq_prepare_annotation.py` script always operates in "stranded" mode, and explicitly destinguishes between genes on opposing strands. QoRTs, on the other hand, prepares the flattened annotation in stranded or unstranded modes. If the DEXSeq-style behavior is desired, a stranded flat gff file can be produced by the `makeFlatGff` utility, then passed explicitly to the QoRTs QC utility running in non-stranded mode using the `--flatgff` parameter. This will override the default (and recommended) behavior in which the flattened gff will use the same stranded rule as the counting utility. Warning: if this variation is used, the counting run should be restricted to only the DEXSeq-count utility using the additional parameter: "–runFunctions writeDEXSeq". The behavior of the other QoRTs utilities when run with a nonmatching flattened annotation is not defined.

In general, testing aggregate genes in DEXSeq is not recommended, as the two genes are likely to be independently regulated and will likely produce false positives. For most purposes it is preferable to drop such genes from the count tables prior to DEXSeq analysis. In this case, the counts produced by DEXSeq and QoRTs will be identical.

References

[1] Simon Anders and Wolfgang Huber. Differential expression analysis for sequence count data. *Genome Biology*, 11:R106, 2010. URL: http://genomebiology.com/2010/11/10/R106.

[2] Mark D. Robinson and Gordon K. Smyth. Moderated statistical tests for assessing differences in tag abundance. *Bioinformatics*, 23:2881, 2007. URL: http://bioinformatics.oxfordjournals.org/cgi/content/abstract/23/21/2881, http://arxiv.org/abs/http://bioinformatics.oxfordjournals.org/cgi/reprint/23/21/2881.pdf arXiv:http://bioinformatics.oxfordjournals.org/cgi/reprint/23/21/2881.pdf, doi:10.1093/bioinformatics/btm453.

[3] Simon Anders, Alejandro Reyes, and Wolfgang Huber. Detecting differential usage of exons from RNA-seq data. *Genome Research*, 22:2008, 2012. doi:10.1101/gr.133744.111.

[4] Alexander Dobin, Carrie A. Davis, Felix Schlesinger, Jorg Drenkow, Chris Zaleski, Sonali Jha, Philippe Batut, Mark Chaisson, and Thomas R. Gingeras. STAR: ultrafast universal RNA-seq aligner. *Bioinformatics*, 29(1):15–21, 2013. URL: http://bioinformatics.oxfordjournals.org/content/29/1/15.abstract, http://arxiv.org/abs/http://bioinformatics.oxfordjournals.org/content/29/1/15.full.pdf+html arXiv:http://bioinformatics.oxfordjournals.org/content/29/1/15.full.pdf+html, doi:10.1093/bioinformatics/bts635.

[5] Thomas D. Wu and Serban Nacu. Fast and SNP-tolerant detection of complex variants and splicing in short reads. *Bioinformatics*, 26(7):873–881, 2010. URL: http://bioinformatics.oxfordjournals.org/content/26/7/873.abstract, http://arxiv.org/abs/http://bioinformatics.oxfordjournals.org/content/26/7/873.full.pdf+html arXiv:http://bioinformatics.oxfordjournals.org/content/26/7/873.full.pdf+html, doi:10.1093/bioinformatics/btq057.

[6] Daehwan Kim, Geo Pertea, Cole Trapnell, Harold Pimentel, Ryan Kelley, and Steven Salzberg. Tophat2: accurate alignment of transcriptomes in the presence of insertions, deletions and gene fusions. *Genome Biology*, 14(4):R36, 2013. URL: http://genomebiology.com/2013/14/4/R36, http://dx.doi.org/10.1186/gb-2013-14-4-r36 doi:10.1186/gb-2013-14-4-r36.

[7] McCarthy DJ Robinson MD and Smyth GK. edger: a bioconductor package for differential expression analysis of digital gene expression data. *Bioinformatics*, 26:139–140, 2010.

[8] Davis J. McCarthy, Yunshun Chen, and Gordon K. Smyth. Differential expression analysis of multifactor RNA-Seq experiments with respect to biological variation. *Nucleic Acids Research*, 40:4288–4297, 2012.

10 Session Information

The session information records the versions of all the packages used in the generation of the present document.

```
sessionInfo()

## R version 3.1.1 (2014-07-10)
## Platform: x86_64-unknown-linux-gnu (64-bit)
##
## locale:
## [1] C
##
## attached base packages:
## [1] parallel  stats4    stats     graphics  grDevices utils     datasets
## [8] methods   base
##
## other attached packages:
##  [1] edgeR_3.8.2              limma_3.22.1             DESeq2_1.6.1
##  [4] RcppArmadillo_0.4.500.0 Rcpp_0.11.3              GenomicRanges_1.18.1
##  [7] GenomeInfoDb_1.2.2      IRanges_2.0.0            S4Vectors_0.4.0
## [10] BiocGenerics_0.12.0     QoRTsExampleData_0.2.0   QoRTs_0.3.4
## [13] Cairo_1.5-6             knitr_1.7
##
## loaded via a namespace (and not attached):
##  [1] AnnotationDbi_1.28.1 BBmisc_1.8           BatchJobs_1.5
##  [4] Biobase_2.26.0       BiocParallel_1.0.0   BiocStyle_1.4.1
##  [7] DBI_0.3.1            Formula_1.1-2        Hmisc_3.14-5
## [10] MASS_7.3-35          RColorBrewer_1.0-5   RSQLite_1.0.0
## [13] XML_3.98-1.1         XVector_0.6.0        acepack_1.3-3.3
## [16] annotate_1.44.0      base64enc_0.1-2      brew_1.0-6
## [19] checkmate_1.5.0      cluster_1.15.3       codetools_0.2-9
## [22] colorspace_1.2-4     digest_0.6.4         evaluate_0.5.5
## [25] fail_1.2             foreach_1.4.2        foreign_0.8-61
## [28] formatR_1.0          genefilter_1.48.1    geneplotter_1.44.0
## [31] ggplot2_1.0.0        grid_3.1.1           gtable_0.1.2
## [34] highr_0.4            iterators_1.0.7      lattice_0.20-29
## [37] latticeExtra_0.6-26  locfit_1.5-9.1       munsell_0.4.2
## [40] nnet_7.3-8           plyr_1.8.1           proto_0.3-10
## [43] reshape2_1.4         rpart_4.1-8          scales_0.2.4
## [46] sendmailR_1.2-1      splines_3.1.1        stringr_0.6.2
## [49] survival_2.37-7      tools_3.1.1          xtable_1.7-4
```

11 Legal

This software is "United States Government Work" under the terms of the United States Copyright Act. It was written as part of the authors' official duties for the United States Government and thus cannot be copyrighted. This software is freely available to the public for use without a copyright notice.

Restrictions cannot be placed on its present or future use.

Although all reasonable efforts have been taken to ensure the accuracy and reliability of the software and data, the National Human Genome Research Institute (NHGRI) and the U.S. Government does not and cannot warrant the performance or results that may be obtained by using this software or data. NHGRI and the U.S. Government disclaims all warranties as to performance, merchantability or fitness for any particular purpose.

In any work or product derived from this material, proper attribution of the authors as the source of the software or data should be made, using "NHGRI Genome Technology Branch" as the citation.

The QoRTs Scala package includes (internally) the sam-JDK library (sam-1.113.jar) from picard tools, which is licensed under the MIT license: The MIT License Copyright (c) 2009 The Broad Institute Permission is hereby granted, free of charge, to any person obtaining a copy of this software and associated documentation files (the "Software"), to deal in the Software without restriction, including without limitation the rights to use, copy, modify, merge, publish, distribute, sublicense, and/or sell copies of the Software, and to permit persons to whom the Software is furnished to do so, subject to the following conditions: The above copyright notice and this permission notice shall be included in all copies or substantial portions of the Software. THE SOFTWARE IS PROVIDED "AS IS", WITHOUT WARRANTY OF ANY KIND, EXPRESS OR IMPLIED, INCLUDING BUT NOT LIMITED TO THE WARRANTIES OF MERCHANTABILITY, FITNESS FOR A PARTICULAR PURPOSE AND NONINFRINGEMENT. IN NO EVENT SHALL THE AUTHORS OR COPYRIGHT HOLDERS BE LIABLE FOR ANY CLAIM, DAMAGES OR OTHER LIABILITY, WHETHER IN AN ACTION OF CONTRACT, TORT OR OTHERWISE, ARISING FROM, OUT OF OR IN CONNECTION WITH THE SOFTWARE OR THE USE OR OTHER DEALINGS IN THE SOFTWARE.

The MIT license and copyright information can also be accessed using the command:

```
java -jar /path/to/jarfile/QoRTs.jar "?" samjdkinfo
```
