# Supplementary material for: QoRTs: a comprehensive toolset for quality control and data processing of RNA-Seq experiments
Source: BMC Bioinformatics. 2015 Jul 19;16(1):224. doi: 10.1186/s12859-015-0670-5 (PMC4506620; doi:10.1186/s12859-015-0670-5)
Supplement: Additional file 2: — Documentation of some of the errors and flaws found with the RSeQC package. [file 12859_2015_670_MOESM2_ESM.pdf]

Documentation of Issues with the RSeQC Package

Stephen Hartley
National Human Genome Research Institute
National Institutes of Health

March 12, 2015

Contents

1 Overview **2**

2 Utilities that return incorrect results **3**
- 2.1 Clipping Profile . . . 3
  - 2.1.1 Example Data . . . 3
  - 2.1.2 Results . . . 5
- 2.2 Insertion Profile . . . 6
  - 2.2.1 Example Data . . . 6
  - 2.2.2 Results . . . 8
- 2.3 Insert Size . . . 9
  - 2.3.1 Example Data . . . 9
  - 2.3.2 Results . . . 11
- 2.4 Read Counts . . . 12
  - 2.4.1 Example Data . . . 12
  - 2.4.2 Results . . . 14
- 2.5 NVC . . . 16
  - 2.5.1 Example Data . . . 16
  - 2.5.2 Results . . . 17

3 Utilities that return no results **18**
- 3.1 Quality score distribution . . . 18
- 3.2 Gene-Body Coverage . . . 20

4 Appendix: Example Annotation **21**

1 Overview

While RSeQC ostensibly offers much of the same functionality as QoRTs, the results from the two tools consistently disagree on a number of metrics. In this supplement we demonstrate several of these inconsistencies, and show that in all of these cases the results provided by QoRTs are accurate while the RSeQC results are not. This supplement is not comprehensive: the reader should not assume that the RSeQC functions not listed here are accurate. The specific flaws demonstrated here are only a subset of the inconsistencies that we have detected between QoRTs and RSeQC. Isolating, demonstrating, and documenting every individual conflict would be arduous and excessive.

We have generated a set of extremely small and simple example sam/bam files. Each file contains 1-14 reads, and each simulated read is only 10 base-pairs long. We have also created a simulated genome annotation containing a single 10000-bp chromosome and few small ”genes” (see the appendix in Section 4). These simplistic example files are designed such that the ”true” values for the various quality control metrics can easily be confirmed simply by viewing the files manually as text or by loading them into viewing software. We recommend either the UCSC genome browser or the IGV utility.

These tests do not employ unusual edge-cases; the errors reproduced here were first identified in real datasets before being isolated via simulated data.

Our goal in this supplement is to demonstrate that in numerous test cases QoRTs produces results that are demonstrably accurate, and RSeQC produces results that are clearly erroneous. The errors outlined in this document reveal that numerous systematic flaws exist in the RSeQC package, and that the package has not undergone sufficient testing during development. Because these errors may conceal actual quality control issues, or may make nonexistent quality control issues appear where no such problems exist, we recommend that RSeQC not be used for quality control of RNA-Seq data.

All the tests included in this supplement were run using QoRTs v0.2.5 and RSeQC 2.6.1, which were both current as of March 12th, 2015. All the data used in this supplement including example sam/bam files, scripts, and raw plots, are available as a separate supplement, currently available online (`https://dl.dropboxusercontent.com/u/103621176/QoRTs/supp/suppTestData.zip`).

2 Utilities that return incorrect results

Several RSeQC utilities run to completion without throwing any errors, but return results that are misleading and/or erroneous. This section contains a subset of these inconsistencies.

2.1 Clipping Profile

The RSeQC appears to uniformly return incorrect results for the ”clipping profile”. The bug appears to be related to which strand the reads are aligned to. Note that the bug appears whether the data is strand-specific or not.

2.1.1 Example Data

For this test we use example sam file test.clip.sam, a single-ended sam file containing 10 reads.

- read 1: fwd strand, no clipping.
- read 2: fwd strand, 1-base clip at the 5’ end.
- read 3: fwd strand, 2-base clip at the 5’ end.
- read 4: fwd strand, 3-base clip at the 5’ end.
- read 5: fwd strand, 4-base clip at the 5’ end.
- read 6: rev strand, no clipping.
- read 7: rev strand, 1-base clip at the 5’ end.
- read 8: rev strand, 2-base clip at the 5’ end.
- read 9: rev strand, 3-base clip at the 5’ end.
- read 10: rev strand, 4-base clip at the 5’ end.

The complete sam file (`test.clip.sam`) follows:

```
@HD VN:1.3 SO:coordinate
@SQ SN:chr1 LN:100000
read:01 0 chr1 10 255 10M * 0 0 AAAAAAAAAA CCCFFFFFHH
read:02 0 chr1 10 255 1S9M * 0 0 GTAAAAAAAA BBBDDDEDCD
read:03 0 chr1 10 255 2S8M * 0 0 GGTAAAAAAA @CCFFFFDHD
read:04 0 chr1 10 255 3S7M * 0 0 GGGTAAAAAA @@@FDFDFFF
read:05 0 chr1 10 255 4S6M * 0 0 GGGGTAAAAA @@CFFFFFHH
read:06 16 chr1 20 255 10M * 0 0 AAAAAAAAAA C@BFFFFFHH
read:07 16 chr1 20 255 9M1S * 0 0 AAAAAAAATG CDDDDDDDDD
read:08 16 chr1 20 255 8M2S * 0 0 AAAAAAATGG +51DBDDDCC
read:09 16 chr1 20 255 7M3S * 0 0 AAAAAATGGG BCCFFDDFHH
read:10 16 chr1 20 255 6M4S * 0 0 AAAAATGGGG BDDDEDDCAD
```

See Figure 1 for a visualization of this sam file:

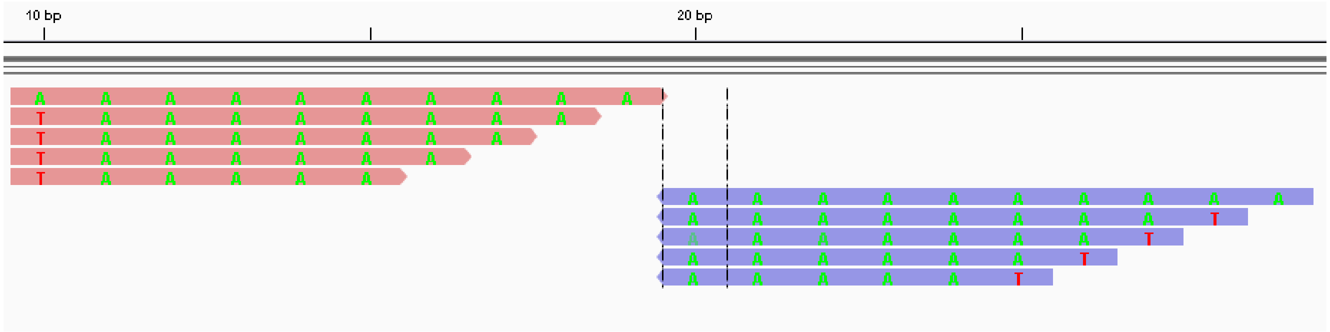

Figure 1: The file test.clip.bam, viewed via IGV.

To run the RSeQC `clipping_profile` script, we used the command:

```
clipping_profile.py -i test.clip.sam -o out
```

And for QoRTs:

```
java -jar /path/to/jar/QoRTs.jar QC \
          --singleEnded \
          --runFunctions CigarOpDistribution --generateSeparatePlots \
          test.clip.sam anno.gtf ./clip/
```

2.1.2 Results

RSeQC appears to have a bug where it does not properly account for the read being reversed when it is aligned to the reverse (genomic) strand (See Figure 2). This is why clipping profiles from RSeQC always appear uniformly symmetric, despite the fact that for most datasets and aligners soft clipping can be expected to be very asymmetric.

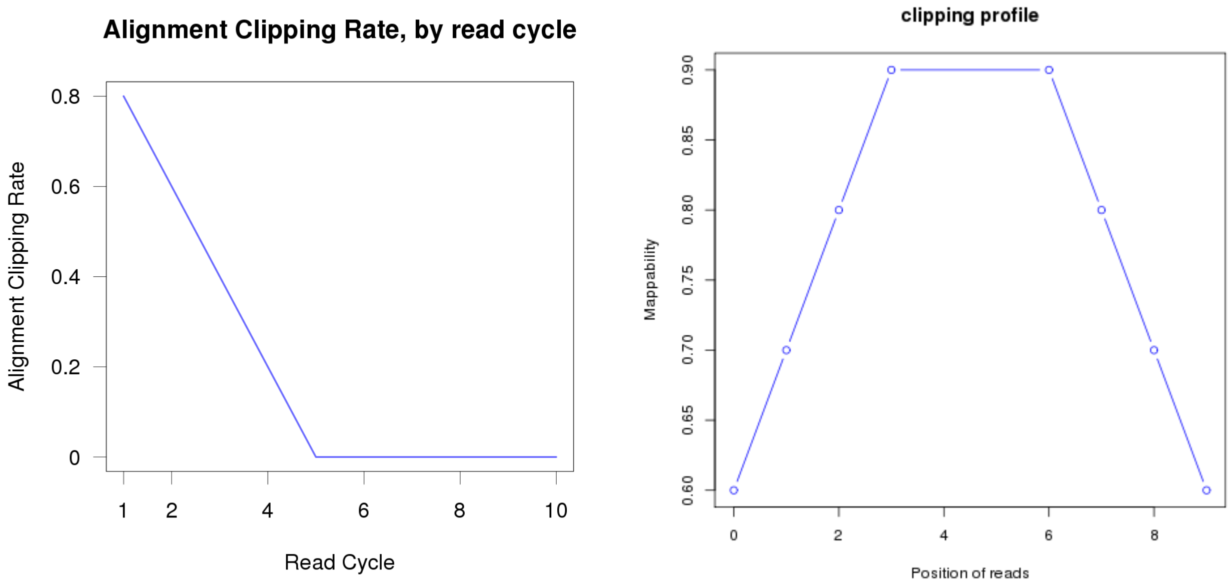

Figure 2: Clipping profile from QoRTs (left) and RSeQC (right). Note that QoRTs plots the clipping rate whereas RSeQC plots the inverse, thus the two plots should be identical when inverted.

As a minor aside: RSeQC does not have a paired-end mode that separates the 1st and 2nd read of each pair separately. Instead it always counts each read independently. This would only affect paired-end data, and is not demonstrated here.

2.2 Insertion Profile

RSeQC returns different results for the insertion profile. Unlike in the Section 2.1, it is very unclear exactly what RSeQC is doing wrong. However it is clear that the results are in fact incorrect.

2.2.1 Example Data

For this test we use example sam file test.ins.sam, a single-ended sam file containing 10 reads.

- reads 1 to 5: fwd strand, No insertions.
- read 6: fwd strand, 3-base insertion over read positions 1-3.
- read 7: fwd strand, 2-base insertion over read positions 2-3.
- read 8: rev strand, 2-base insertion over read positions 4-5.
- read 9: fwd strand, 3-base insertion over read positions 6-8.
- read 10: rev strand, 3-base insertion over read positions 6-8.

The complete sam file (`test.ins.sam`) follows:

```
@HD VN:1.3 SO:coordinate
@SQ SN:chr1 LN:100000
read:01 0 chr1 10 255 10M * 0 0 AAAAAAAAAA CCCFFFFFHH
read:02 0 chr1 10 255 10M * 0 0 AAAAAAAAAA C@BFFFFFHH
read:03 0 chr1 10 255 10M * 0 0 AAAAAAAAAA CCCFFFFFHH
read:04 0 chr1 10 255 10M * 0 0 AAAAAAAAAA C@BFFFFFHH
read:05 0 chr1 10 255 10M * 0 0 AAAAAAAAAA CCCFFFFFHH
read:06 0 chr1 20 255 3I7M * 0 0 GGGTAAAAAA C@BFFFFFHH
read:07 0 chr1 20 255 1M2I7M * 0 0 TGGTAAAAAA @@@FDFDFFF
read:08 16 chr1 20 255 5M2I3M * 0 0 AAAATGGTAA +51DBDDDCC
read:09 0 chr1 20 255 5M3I2M * 0 0 AAAATGGGTA @@CFFFFFHH
read:10 16 chr1 20 255 2M3I5M * 0 0 ATGGGTAAAA CDDDDDDDDD
```

See Figure 3 for a visualization of this sam file:

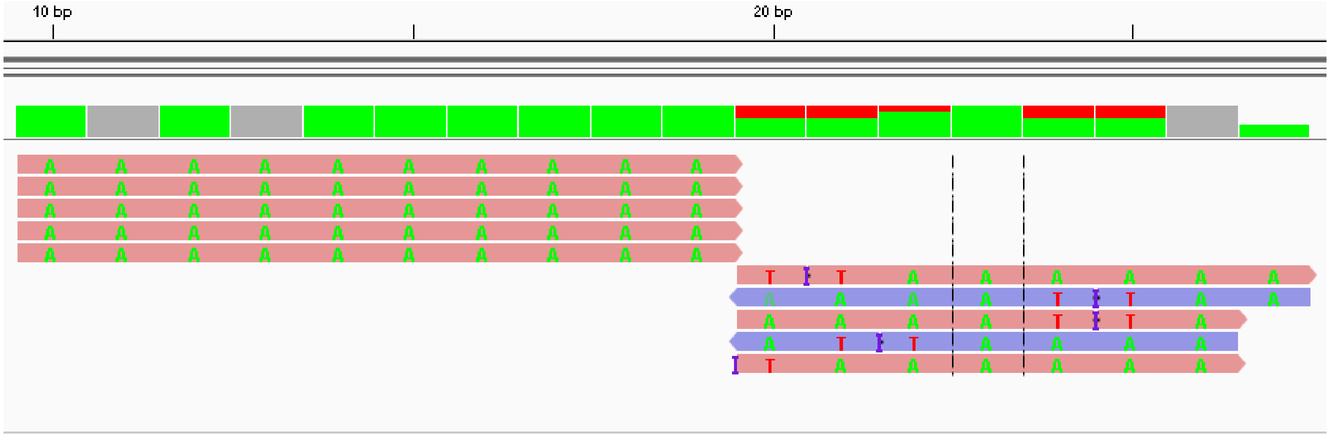

Figure 3: The file test.ins.bam, viewed via IGV.

Note: The correct insertion profile is calculated by hand in the table below. Insertion bases are marked with X's, and the bottom row lists the total number of insertions found at the given read position.

Insertion Profile

Read Position:	1	2	3	4	5	6	7	8	9	10
read 06:	X	X	X							
read 07:		X	X							
read 08: (rev)				X	X					
read 09:						X	X	X		
read 10: (rev)						X	X	X		
TOTAL:	1	2	2	1	1	2	2	2	0	0

Note that this table matches the plot produced by QoRTs (see Figure 4).

To run the RSeQC `insertion_profile` script, we used the command:

```
insertion_profile.py -i test.ins.sam -o out -l 10 -n 10
```

And for QoRTs:

```
java -jar /path/to/jar/QoRTs.jar QC
          --singleEnded \
          --runFunctions CigarOpDistribution --generateSeparatePlots \
          test.ins.sam anno.gtf ./ins/
```

2.2.2 Results

Unlike in Section 2.1, it is not at all obvious what RSeQC is doing wrong when it calculates the insertion profile.

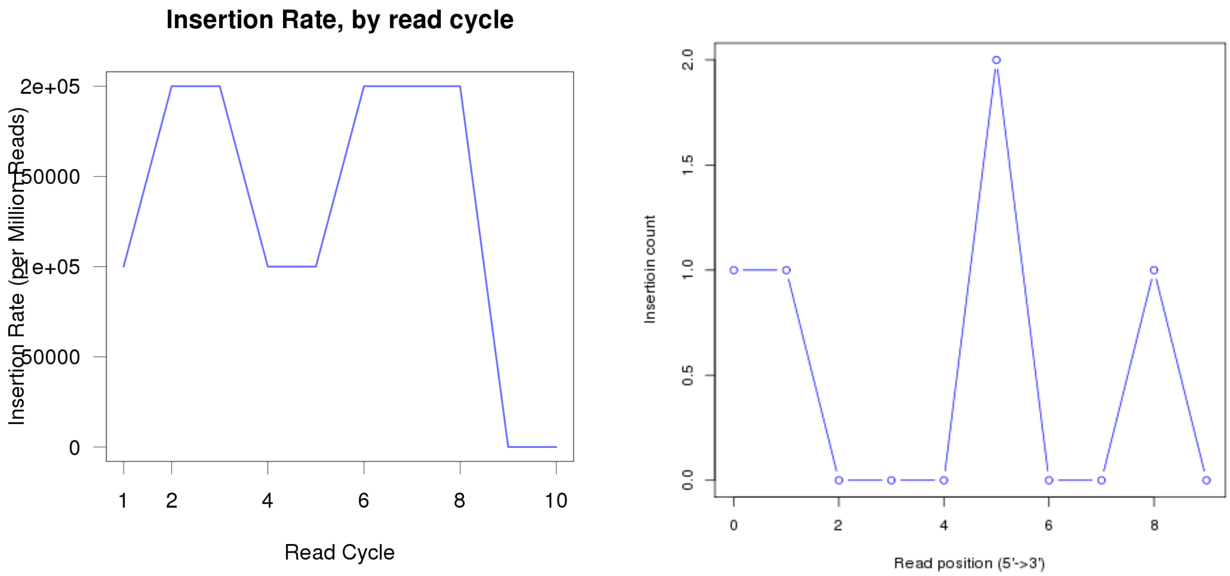

Figure 4: Insertion profile from QoRTs (left) and RSeQC (right)

See Figure 4. At first it appeared that (as in the previous section) RSeQC was not correctly reversing reads aligned to the reverse (genomic) strand. However this still does not fully explain the incongruity.

While it's not clear precisely what is going wrong with the RSeQC utility, it is clear that the supplied answer is incorrect.

2.3 Insert Size

RSeQC calculates "inner distance" instead of insert size. The inner distance is the distance between the inner endpoints of the two paired reads. The insert size (ie, the distance between the outer endpoints of the two paired reads) will be equal to the inner distance plus the length of both reads. For the example dataset, this means the insert size is equal to the inner distance plus 20. Note that this test uses the simulated annotation data (see the appendix in Section 4).

RSeQC appears to mis-calculate the inner distance/insert size under certain common conditions. It's not entirely clear what specifically those conditions are.

2.3.1 Example Data

For this test we use example sam file test.paired.7.sam, a paired-ended sam file containing 7 read-pairs (14 reads total).

- read-pair 1: Insert size 13, read 1 bridges an 17-bp (novel) junction.
- read-pair 2: Insert size 11, no splices.
- read-pair 3: Insert size 12, both reads have a 4-bp splice
- read-pair 4: Insert size 26, the reads flank a 4-bp annotated splice junction, but are on the wrong strand
- read-pair 5: Insert size 22, the reads flank a 4-bp annotated splice junction.
- read-pair 6: Insert size 10, the reads overlap completely, and both bridge the same 9bp splice junction
- read-pair 7: Insert size 15, both reads bridge two 3-bp splice junctions.

The complete sam file (`test.paired.7.sam`) follows:

```
@HD VN:1.3 SO:coordinate
@SQ SN:chr1 LN:100000
read:01 163 chr1 7   255 3M17N7M    = 27  30 CTCCTCGGAA 85BC?=@ACC
read:01 83  chr1 27  255 10M        = 7   30 GGGGTGAGGC CCCFFFFFGG
read:02 99  chr1 40  255 10M        = 41  11 CTCTGTTTAT CCCFFFFFHH
read:02 147 chr1 41  255 10M        = 40  11 CCGATCTCTC BBBDDDEDCD
read:03 99  chr1 45  255 6M4N4M     = 47  16 GTTGAAACTT @CCFFFFDHD
read:03 147 chr1 47  255 4M4N6M     = 45  16 ACTGCCCTCT @@@FDFDFFF
read:04 99  chr1 55  255 10M        = 71  30 GATCTGTCCA @@CFFFFFHH
read:05 163 chr1 55  255 10M        = 71  30 ATAGCACCAT @@CFFFFFHH
read:04 147 chr1 71  255 5M4N5M     = 55  30 TCATCGCAGA CC@FDFFFHH
read:05 83  chr1 71  255 5M4N5M     = 55  30 NTCCAGACAG CC@FDFFFHH
read:06 163 chr1 85  255 6M9N4M     = 85  19 GCCACCTTTT 955(>5CA?9
read:06 83  chr1 85  255 6M9N4M     = 85  19 CACCTTTTCT <<>5>:>59,
read:07 163 chr1 100 255 6M3N2M3N2M = 105 21 CTCCTCGGAA 85BC?=@ACC
read:07 83  chr1 105 255 1M3N2M3N7M = 100 21 GGGGTGAGGC CCCFFFFFGG
```

See Figure 5 for a visualization of this sam file:

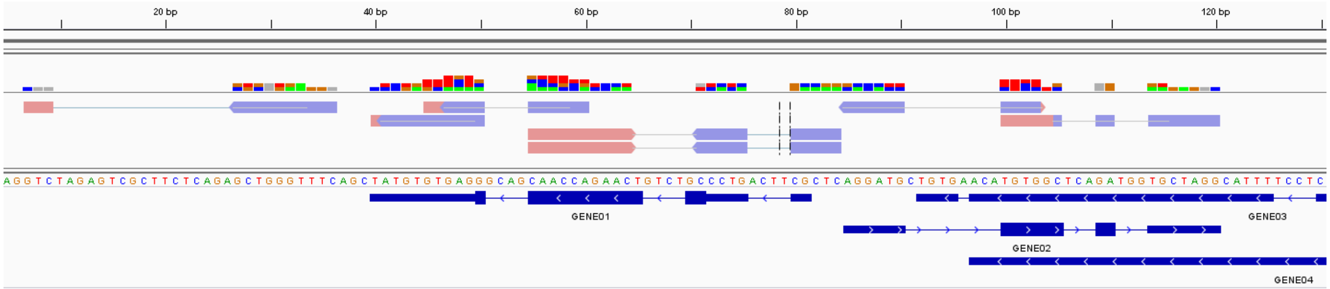

Figure 5: The file test.paired.7.bam, viewed via IGV.

To run the RSeQC `insertion_profile` script, we used the command:

```
inner_distance.py -i test.paired.7.sam -o out -r anno.bed
```

And for QoRTs:

```
java -jar /path/to/jar/QoRTs.jar QC \
          --runFunctions InsertSize \
          --coordSorted --generateSeparatePlots \
          test.paired.7.sam anno.gtf ./insertSize/
```

2.3.2 Results

RSeQC systematically returns erroneous results under a variety of different circumstances (see Figure 6). There's no clear pattern to these errors, and the discrepancies may be caused by a number of independent bugs.

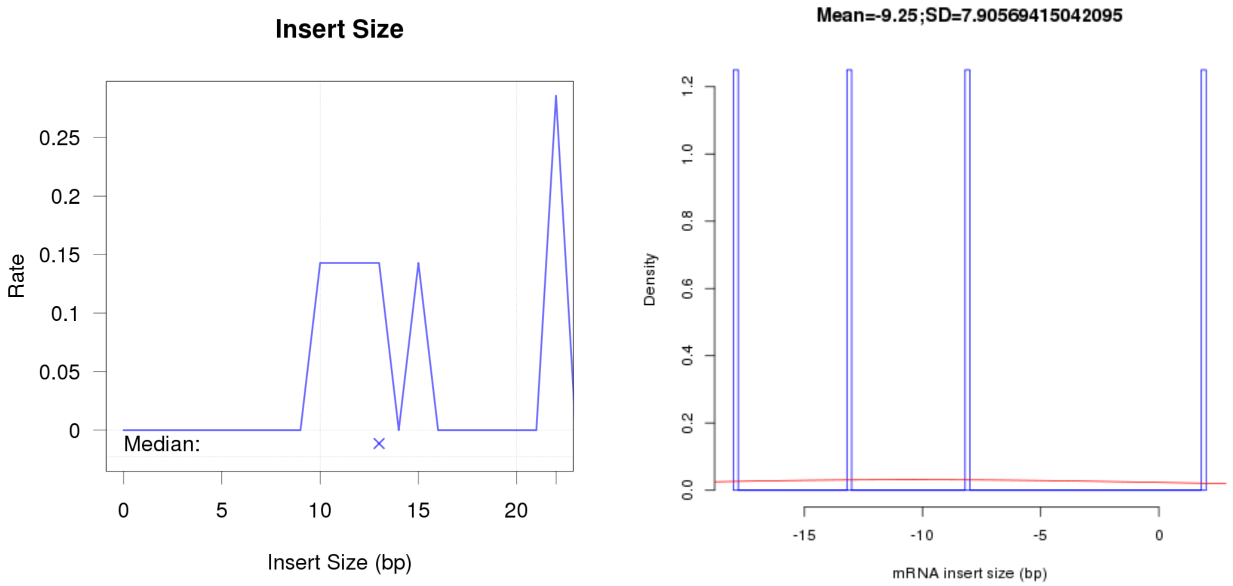

Figure 6: Insert size plot from QoRTs (left), compared with inner distance plot from RSeQC (right). Note that RSeQC bins the frequencies into 5-bp bins so the plots are not directly comparable.

RSeQC also produces a table containing each read and the calculated inner distance. This makes it easy to discern specifically which inner distances were mis-calculated:

Results				
Read ID	True	RSeQC		QoRTs
	Insert Size	Inner Distance	Insert Size	Insert Size
read 1	13	-7	13	13
read 2	11	-9	11	11
read 3	12	-12	8	12
read 4	22	2	22	22
read 5	22	2	22	22
read 6	10	-19	1	10
read 7	15	-11	9	15

Note that read-pairs 3, 6, and 7 are incorrect. In addition to calculating the inner distance incorrectly, RSeQC appears to count read-pair 6 twice, and the read-pair appears twice in the `inner_distance.txt` file. It is not immediately clear why this occurred, or why specifically read 6 was repeated.

Also note that RSeQC does not have a strand-specific mode for calculating inner distances. Thus, RSeQC would incorrectly assume that read-pair 4 flanks an annotated splice junction that lies on the wrong strand. QoRTs, on the other hand, has a strand-specific mode and would correctly assign read-pair 4 an insert size of 26 when run in this mode.

2.4 Read Counts

Direct comparison of QoRTs and RSeQC's read counts is difficult, as the two utilities (by design) use fundamentally different methods. In particular, RSeQC cannot read gtf annotations, which allow the specification of multiple transcripts belonging to the same gene.

HTSeq, QoRTs, and the bioconductor GenomicRanges package all use identical methods to calculate gene-level read counts. Reads that intersect with any section of any known transcript of a gene are counted towards that gene. This is the method recommended for use with differential expression tools like edgeR and DESeq/DESeq2. RSeQC instead treats each transcript as a separate gene and counts reads intersecting with each transcript separately.

Even though the counts are not directly comparable, we can still visually identify cases in which the RSeQC counts are incorrect.

2.4.1 Example Data

For this test we use example sam file test.gene.sam, a single-ended sam file containing 5 reads, all on the forward strand on GENE 5.

- read 1: Covers exon 1. 5 bases clipped from the 5' end.
- read 2: Covers exon 1. 5 bases clipped from the 5' end.
- read 4: Covers exons 1 and 2, bridges a known splice site.
- read 4: Covers exons 1 and 2, bridges a known splice site. 1 base clipped from the 5' end.
- read 5: Covers exons 2 and 3, bridges a known splice site. 4 bases clipped from the 5' end, 2 bases from the 3' end.

The complete sam file (`test.gene.sam`) follows:

```
@HD VN:1.3 SO:coordinate
@SQ SN:chr1 LN:100000
read:01 0 chr1 506 255 5S5M * 0 0 CTCCTCGGAA 85BC?=@ACC
read:02 0 chr1 507 255 5S5M * 0 0 CTCTGTTTAT CCCFFFFFHH
read:03 0 chr1 508 255 3M59N7M * 0 0 CTCCTCGGAA 85BC?=@ACC
read:04 0 chr1 509 255 1S2M59N7M * 0 0 CTCCTCGGAA 85BC?=@ACC
read:05 0 chr1 579 255 4S2M69N2M2S * 0 0 GTTGAAACTT @CCFFFFDHD
```

See Figure 7 for a visualization of this sam file:

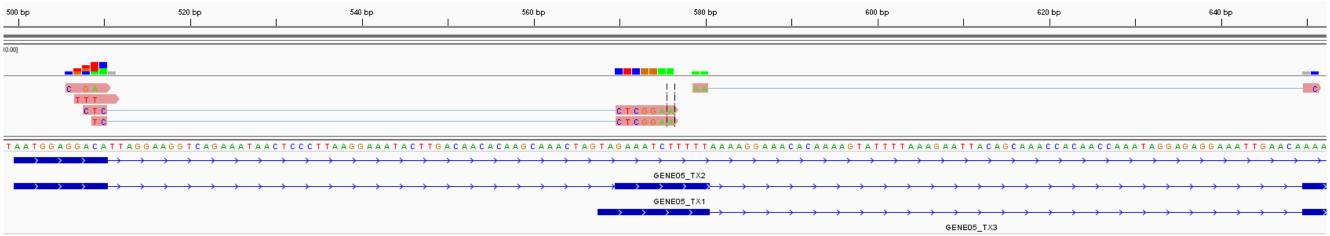

Figure 7: The file test.gene.bam, viewed via IGV.

To run the RSeQC `RPKM_count` script, we used the command:

```
RPKM_count.py -i test.gene.bam -o out -r anno.bed -e
```

And for QoRTs:

```
java -jar ~/UTILS/SCALA/QoRTs.jar QC \
   --runFunctions FPKM,writeSpliceExon,writeGeneCounts,annotatedSpliceExonCounts \
   --generateSeparatePlots --singleEnded \
   test.gene.sam  anno.gtf ./geneCounts/
```

2.4.2 Results

The results for RSeQC are shown below.

chrom	st	end	accession	score	gene_strand	tag_count	RPKM
chr1	510	569	GENE05_TX1_intron_1	0	+	3	12711864.41
chr1	580	649	GENE05_TX1_intron_2	0	+	1	3623188.406
chr1	655	724	GENE05_TX1_intron_3	0	+	0	0
chr1	728	819	GENE05_TX1_intron_4	0	+	0	0
chr1	499	510	GENE05_TX1_exon_1	0	+	1	22727272.73
chr1	569	580	GENE05_TX1_exon_2	0	+	2	45454545.46
chr1	649	655	GENE05_TX1_exon_3	0	+	1	41666666.67
chr1	724	728	GENE05_TX1_exon_4	0	+	0	0
chr1	819	840	GENE05_TX1_exon_5	0	+	0	0
chr1	499	840	GENE05_TX1_mRNA	0	+	4	18867924.53
chr1	510	724	GENE05_TX2_intron_1	0	+	7	8177570.093
chr1	728	819	GENE05_TX2_intron_2	0	+	0	0
chr1	499	510	GENE05_TX2_exon_1	0	+	1	22727272.73
chr1	724	728	GENE05_TX2_exon_2	0	+	0	0
chr1	819	840	GENE05_TX2_exon_3	0	+	0	0
chr1	499	840	GENE05_TX2_mRNA	0	+	1	6944444.444
chr1	580	649	GENE05_TX3_intron_1	0	+	1	3623188.406
chr1	657	809	GENE05_TX3_intron_2	0	+	0	0
chr1	567	580	GENE05_TX3_exon_1	0	+	2	38461538.46
chr1	649	657	GENE05_TX3_exon_2	0	+	1	31250000
chr1	809	837	GENE05_TX3_exon_3	0	+	0	0
chr1	567	837	GENE05_TX3_mRNA	0	+	3	15306122.45

There are several unexplained errors here:

- The first exon (exon 1 of TX1 and TX2) shows 1 read rather than 4.
- The second exon (exon 2 of TX1 and TX2, exon 1 of TX3) shows 2 reads rather than 3.
- Feature `"GENE05_TX2_intron_1"` has a read-count of 7, despite there being only 5 reads in the file.
- The RPKM values appear to be calculated based on a total read count of 4 rather than 5. Note: this does not appear to be a simple off-by-one error. In other datasets it appears to be off by more.

Note that while QoRTs does not calculate the exact same metrics as RSeQC, it does produce some similar metrics. QoRTs internally generates a ”flattened” exon annotation composed of the set mutually-exclusive exon segments for all transcripts belonging to each gene. Each exon-segment is then assigned a unique identifier. This uses the same methods used by DEXSeq. QoRTs also generates coverage counts for all splice junctions (both known and novel).

The following is an excerpt from QC.annoSpliceJunctionAndExonCounts.txt.gz:

featureID	featureType	chrom	start	end	strand	geneID	binID	readCount
GENE05:A000	aggregate_gene	chr1	500	840	.	GENE05	0	5
GENE05:E001	exonic_part	chr1	500	510	.	GENE05	1	4
GENE05:E002	exonic_part	chr1	568	569	.	GENE05	2	0
GENE05:E003	exonic_part	chr1	570	580	.	GENE05	3	3
GENE05:E004	exonic_part	chr1	650	655	.	GENE05	4	1
GENE05:E005	exonic_part	chr1	656	657	.	GENE05	5	0
GENE05:E006	exonic_part	chr1	725	728	.	GENE05	6	0
GENE05:E007	exonic_part	chr1	810	819	.	GENE05	7	0
GENE05:E008	exonic_part	chr1	820	837	.	GENE05	8	0
GENE05:E009	exonic_part	chr1	838	840	.	GENE05	9	0
GENE05:J010	splice_site	chr1	511	569	.	GENE05	10	2
GENE05:J011	splice_site	chr1	511	724	.	GENE05	11	0
GENE05:J012	splice_site	chr1	581	649	.	GENE05	12	1
GENE05:J013	splice_site	chr1	656	724	.	GENE05	13	0
GENE05:J014	splice_site	chr1	658	809	.	GENE05	14	0
GENE05:J015	splice_site	chr1	729	819	.	GENE05	15	0

This file compiles coverage counts for 3 types of features: aggregate genes, exonic segments, and (known) splice sites. Coverage of unannotated splice sites are listed in a separate file.

Exon segment E001 corresponds to exon 1 of TX1 and TX2. Exon segment E002 corresponds to the small section of the first exon of TX3 that does not also belong to exon 2 of TX1 and TX2. Exon segment E003 corresponds to the area shared by exon 2 of TX1 and TX2 and exon 1 of TX3. Finally, exon segment E004 corresponds to the overlapping sections of exon 3 for TX1/TX2 and exon 2 of TX3. Comparison of this table to the original bam file reveals that all the counts are accurate.

2.5 NVC

Both RSeQC and QoRTs produce a nucleotide-vs-cycle plot, which is supposed to plot the nucleotide-base composition as a function of sequencer cycle. However, RSeQC's NVC plot instead plots nucleotide vs read position. For single-ended read data this is equivalent, but for paired-end data the same position on two paired reads would come from completely different sequencer cycles. QoRTs counts the two reads separately thus generating separate base rates for each sequencer cycle.

Note that this issue is less a bug, and arguably nothing more than a difference in methodology. However: as many quality issues will be specific to sequencer cycles, merging the two reads may obscure artifacts or errors.

This problem only exists for paired-end data. For single-ended data the two utilities appear to consistently return identical results.

2.5.1 Example Data

The example dataset is the same sam file used for the inner distance test in Section 2.3.

To run RSeQC's `read_NVC` script, we used the command:

```
read_NVC.py -i test.paired.7.sam -o out
```

And for QoRTs:

```
java -jar ~/UTILS/SCALA/QoRTs.jar QC --runFunctions NVC \
                --coordSorted --generateSeparatePlots \
                test.paired.7.sam anno.gtf ./NVC/
```

2.5.2 Results

The results are shown below, see Figure 8.

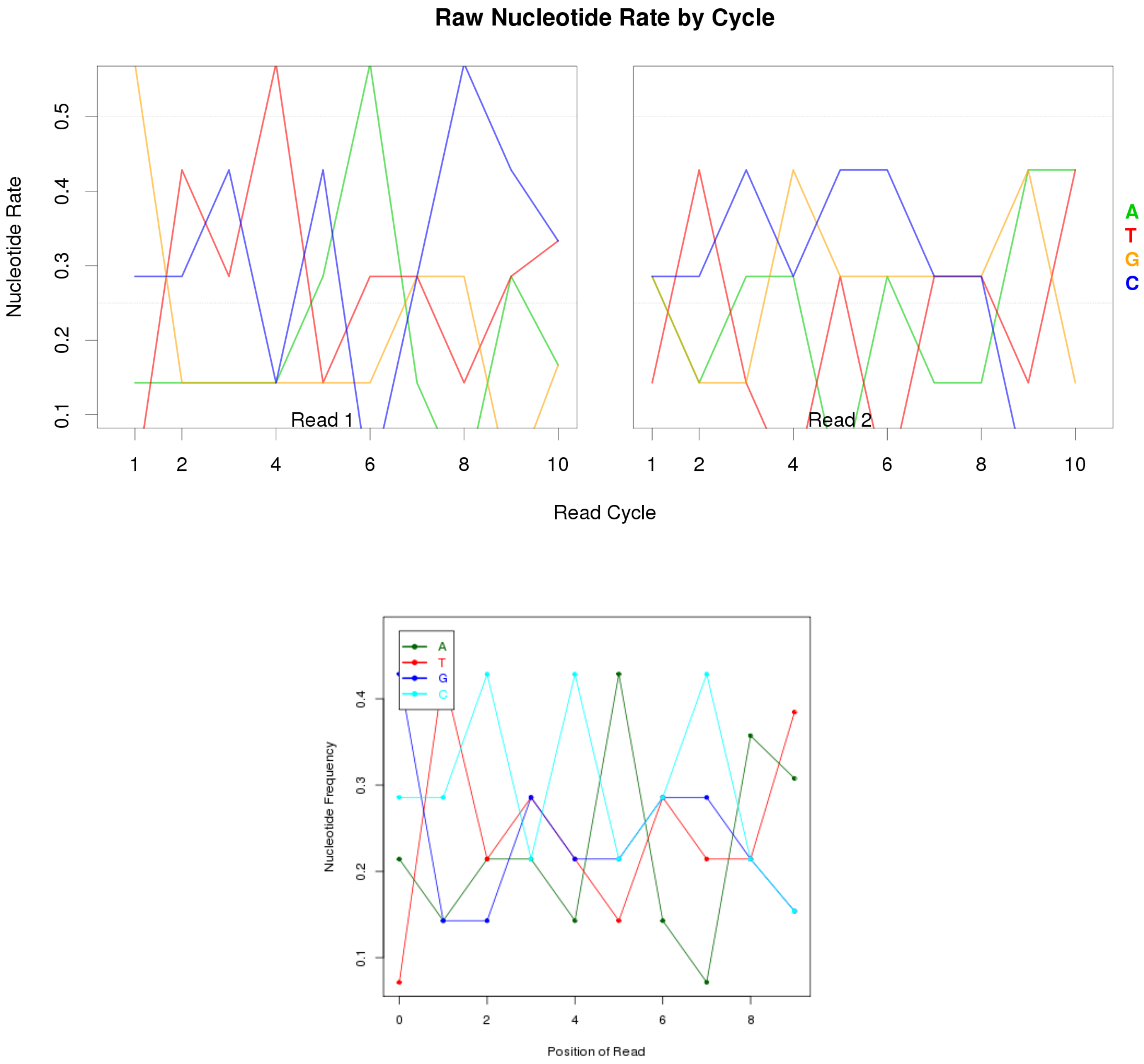

Figure 8: NVC plots from QoRTs (top) and RSeQC (bottom)

3 Utilities that return no results

Some of the RSeQC utilities could not be accurately assessed under controlled conditions, because when given various test sets they simply crashed and/or returned no results.

Some of these errors may be related to the simplicity and small size of the dataset. Nevertheless, these flaws make it much more difficult to definitively assess the accuracy of these utilities. Numerous simulated datasets were created and tested, but we were unable to find test sets that were simple enough to verify results visually but that did not also cause RSeQC to fail.

3.1 Quality score distribution

To run the RSeQC `read_quality` script, we used the command:

```
read_quality.py -i test.paired.7.sam -o out
```

This produced the following output:

```
========================================================
Starting read_quality.py
Read SAM file ...  Done
Error in plot.window(xlim = xlim, ylim = ylim, log = log, yaxs = pars$yaxs) :
  need finite 'ylim' values
Calls: boxplot -> boxplot.default -> do.call -> bxp -> plot.window
In addition: Warning messages:
1: In min(x) : no non-missing arguments to min; returning Inf
2: In max(x) : no non-missing arguments to max; returning -Inf
Execution halted
========================================================
```

In contrast, the QoRTs utility can be run using the command:

```
java -jar ~/UTILS/SCALA/QoRTs.jar QC \
               --runFunctions QualityScoreDistribution \
               --coordSorted --generateSeparatePlots \
               test.paired.7.sam  anno.gtf ./qual/
```

This produces the full set of quality plots. See Figure 9.

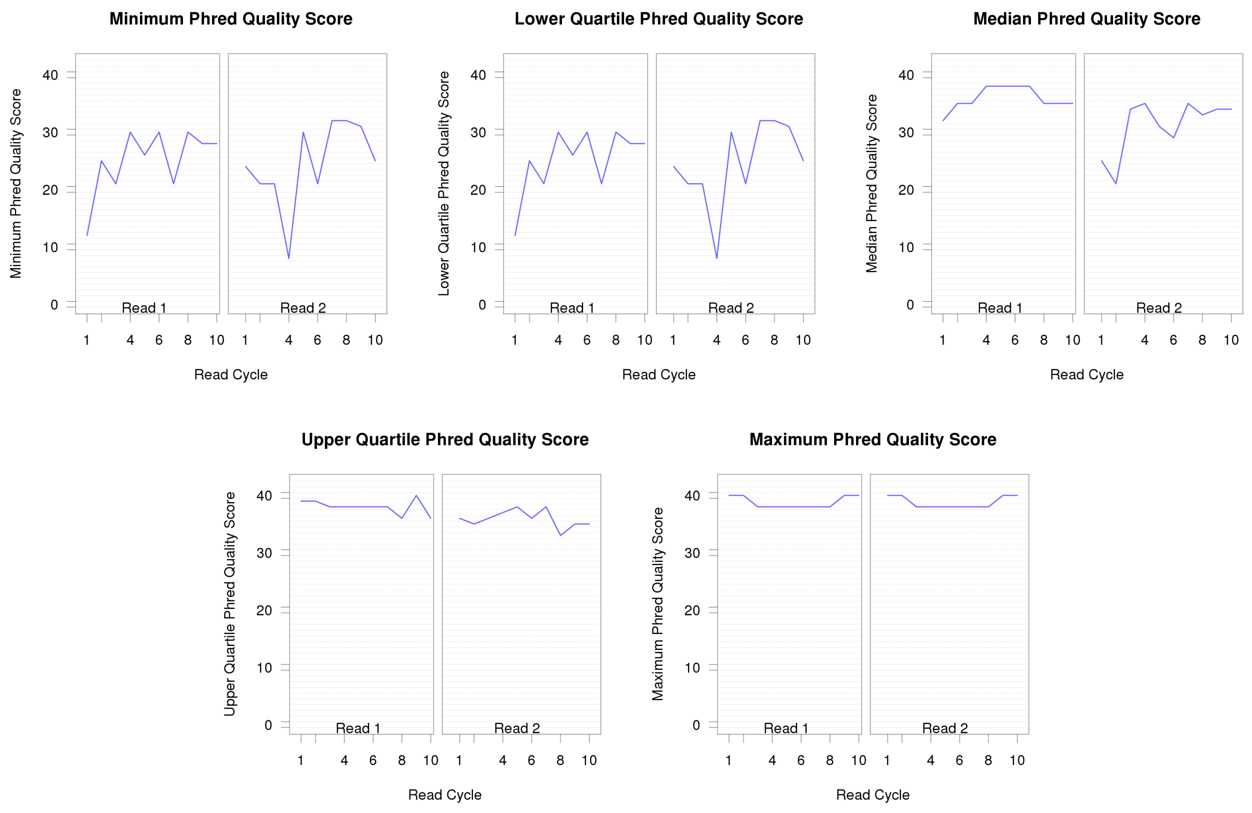

Figure 9: Phred quality score plots from QoRTs.

3.2 Gene-Body Coverage

To run the RSeQC `geneBody_coverage` script, we use the command:

```
geneBody_coverage.py -i test.paired.7.sam -o out -r anno.bed
```

While this did not print any errors, it also did not return any results. The output file "out.geneBodyCoverage.tx only had a title row, and the file "out.geneBodyCoverage.curves.pdf" would not open. Examining the R script file "out.geneBodyCoverage.r", it appears that no plots were generated.

In contrast, the QoRTs utility can be run using the command:

```
java -jar ~/UTILS/SCALA/QoRTs.jar QC \
               --runFunctions writeGeneBody \
               --coordSorted --generateSeparatePlots \
               test.paired.7.sam  anno.gtf ./geneBody/
```

For the purposes of calculating gene body coverage, QoRTs ignores any genes that overlap with other genes, as in these cases it will be impossible to determine the position in the gene body. Thus, in this particular example file the gene-body coverage plot will be based entirely on the coverage of GENE01. See Figure 10.

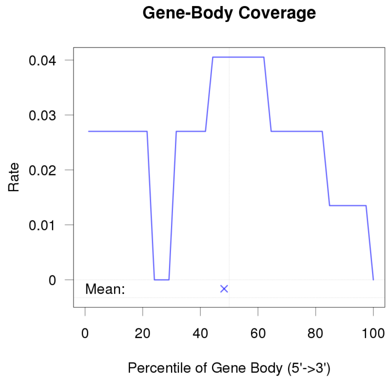

Figure 10: Gene Body plot from QoRTs.

4 Appendix: Example Annotation

In additon to the example sam/bam files, we generated a set of simple simulated annotation files. These annotation files include 5 ”genes”. All genes except for GENE05 have only one single isoform. Both a ”.gtf” file and a ”.bed” file were generated, for use with QoRTs and RSeQC, respectively. For more information on these formats, see the UCSC documentation [1]

See Figure 11 for a visualization of these annotation files.

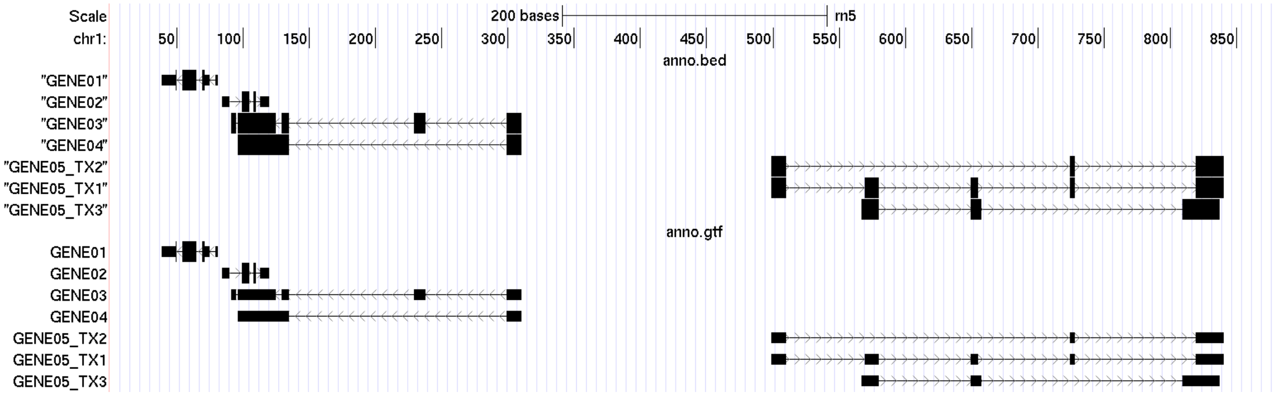

Figure 11: The file anno.gtf and anno.bed, viewed via the UCSC genome browser.

[1] http://genome.ucsc.edu/FAQ/FAQformat.html

The simulated gtf file anno.gtf is:

```
chr1 simulatedData exon 40 50 . -. gene_id "GENE01"; transcript_id "GENE01";
chr1 simulatedData CDS 50 50 . -. gene_id "GENE01"; transcript_id "GENE01";
chr1 simulatedData CDS 55 65 . -. gene_id "GENE01"; transcript_id "GENE01";
chr1 simulatedData exon 55 65 . -. gene_id "GENE01"; transcript_id "GENE01";
chr1 simulatedData CDS 70 71 . -. gene_id "GENE01"; transcript_id "GENE01";
chr1 simulatedData exon 70 75 . -. gene_id "GENE01"; transcript_id "GENE01";
chr1 simulatedData exon 80 81 . -. gene_id "GENE01"; transcript_id "GENE01";
chr1 simulatedData exon 85 90 . + . gene_id "GENE02"; transcript_id "GENE02";
chr1 simulatedData CDS 100 105 . + . gene_id "GENE02"; transcript_id "GENE02";
chr1 simulatedData exon 100 105 . + . gene_id "GENE02"; transcript_id "GENE02";
chr1 simulatedData CDS 109 110 . + . gene_id "GENE02"; transcript_id "GENE02";
chr1 simulatedData exon 109 110 . + . gene_id "GENE02"; transcript_id "GENE02";
chr1 simulatedData exon 114 120 . + . gene_id "GENE02"; transcript_id "GENE02";
chr1 simulatedData exon 92 95 . -. gene_id "GENE03"; transcript_id "GENE03";
chr1 simulatedData exon 97 125 . -. gene_id "GENE03"; transcript_id "GENE03";
chr1 simulatedData exon 130 135 . -. gene_id "GENE03"; transcript_id "GENE03";
chr1 simulatedData exon 230 238 . -. gene_id "GENE03"; transcript_id "GENE03";
chr1 simulatedData exon 300 310 . -. gene_id "GENE03"; transcript_id "GENE03";
chr1 simulatedData exon 97 135 . -. gene_id "GENE04"; transcript_id "GENE04";
chr1 simulatedData exon 300 310 . -. gene_id "GENE04"; transcript_id "GENE04";
chr1 simulatedData exon 500 510 . + . gene_id "GENE05"; transcript_id "GENE05_TX1";
chr1 simulatedData exon 570 580 . + . gene_id "GENE05"; transcript_id "GENE05_TX1";
chr1 simulatedData exon 650 655 . + . gene_id "GENE05"; transcript_id "GENE05_TX1";
chr1 simulatedData exon 725 728 . + . gene_id "GENE05"; transcript_id "GENE05_TX1";
chr1 simulatedData exon 820 840 . + . gene_id "GENE05"; transcript_id "GENE05_TX1";
chr1 simulatedData exon 500 510 . + . gene_id "GENE05"; transcript_id "GENE05_TX2";
chr1 simulatedData exon 725 728 . + . gene_id "GENE05"; transcript_id "GENE05_TX2";
chr1 simulatedData exon 820 840 . + . gene_id "GENE05"; transcript_id "GENE05_TX2";
chr1 simulatedData exon 568 580 . + . gene_id "GENE05"; transcript_id "GENE05_TX3";
chr1 simulatedData exon 650 657 . + . gene_id "GENE05"; transcript_id "GENE05_TX3";
chr1 simulatedData exon 810 837 . + . gene_id "GENE05"; transcript_id "GENE05_TX3";
```

An equivalent bed file, for use with RSeQC was also created: anno.bed:

```
chr1 91 310 "GENE03" 0 -91 310 0 5 4,29,6,9,11 0,5,38,138,208
chr1 96 310 "GENE04" 0 -96 310 0 2 39,11 0,203
chr1 499 840 "GENE05_TX1" 0 + 499 840 0 6 11,11,6,4,4,21 0,70,150,225,225,320
chr1 39 81 "GENE01" 0 -49 71 0 4 11,11,6,2 0,15,30,40
chr1 499 840 "GENE05_TX2" 0 + 499 840 0 3 11,4,21 0,225,320
chr1 84 120 "GENE02" 0 + 99 110 0 4 6,6,2,7 0,15,24,29
chr1 567 837 "GENE05_TX3" 0 + 567 837 0 3 13,8,28 0,82,242
```
